# The motivating effect of monetary over psychological incentives is stronger in WEIRD cultures

**DOI:** 10.1038/s41562-023-01769-5

**Published:** 2024-01-08

**Authors:** Danila Medvedev, Diag Davenport, Thomas Talhelm, Yin Li

**Affiliations:** 1 University of Chicago, Booth School of Business, Chicago, IL USA; 2 Princeton University, School of Public and International Affairs, Princeton, NJ USA; 3 Yale University, Yale School of Management, New Haven, CT USA

**Keywords:** Human behaviour, Business and management

## Abstract

Motivating effortful behaviour is a problem employers, governments and nonprofits face globally. However, most studies on motivation are done in Western, educated, industrialized, rich and democratic (WEIRD) cultures. We compared how hard people in six countries worked in response to monetary incentives versus psychological motivators, such as competing with or helping others. The advantage money had over psychological interventions was larger in the United States and the United Kingdom than in China, India, Mexico and South Africa (*N* = 8,133). In our last study, we randomly assigned cultural frames through language in bilingual Facebook users in India (*N* = 2,065). Money increased effort over a psychological treatment by 27% in Hindi and 52% in English. These findings contradict the standard economic intuition that people from poorer countries should be more driven by money. Instead, they suggest that the market mentality of exchanging time and effort for material benefits is most prominent in WEIRD cultures.

## Abstract (web summary)

Monetary incentives were found to be more motivating than psychological interventions for individuals in the United States and the United Kingdom, compared with individuals in China, India, Mexico and South Africa. Among bilinguals on Facebook, money was more motivating in English compared with in Hindi.

## Main

What motivates people to work harder? Money is a logical starting place. Many modern jobs pay people for their time and effort. Some workers earn money contingent on how many cars they sell, how much data they enter or how many apples they pick. In such jobs, the implicit belief is that the more the employer pays, the more the employee works. The idea is an old one. Max Weber wrote that piece-rates are “one of the technical means which the modern employer uses to secure the greatest amount of work from his men”^1^.

Yet money is not the only source of motivation^2^. In their work, people also respond to psychological motivators, such as social norms, praise, self-actualization, desire to reciprocate and fear of social rejection^3^. A large body of research has shown that both monetary and non-monetary incentives can motivate people in a wide variety of settings^4–7^.

Understanding the (cost-) effectiveness of different incentives has important practical implications. Employers are constantly searching for ways to motivate their workers and improve their performance. Governments and nonprofits face a similar problem. They spend billions of dollars on public campaigns to encourage socially beneficial behaviours such as getting vaccinated or wearing seatbelts. The better we can motivate effortful behaviours, the better our interventions, workplaces and economies will be.

Researchers have been working on this problem recently. Large-scale experiments directly contrasted the effectiveness of monetary and non-monetary incentives in motivating effort^8–10^. While both monetary and non-monetary incentives were found to be motivating, money had a large advantage over psychological motivators.

This line of work is important because it uses strong methods. The studies clearly compared the effectiveness of different incentives, with tight controls and random assignment. However, the samples in both experiments consisted almost entirely of people from the United States and did not consider cultural differences. Can managers and policymakers in India, Indonesia or Nigeria apply scientific insights from these studies to issues facing their own countries?

Researchers have warned against indiscriminately exporting interventions that have demonstrated effectiveness in North America or Western Europe to other world regions^11–17^. Yet so far, most interventions have rarely been tested outside of Western, educated, industrialized, rich and democratic (WEIRD)^18^ cultures^19–21^. For example, a 2018 meta-review looked at the effectiveness of ‘green nudges’— non-monetary incentives to use less electricity and switch to more environmentally friendly energy sources^21^. All 40 studies were conducted in high-income counties^22^, and 35 were in the United States or Western Europe.

This extreme sampling bias is not unique to intervention studies. Studies asking general questions about human nature face a similar problem^18^. Over 90% of all papers published in top psychological journals use samples drawn exclusively from WEIRD cultures^23,24^. Despite this sampling bias, researchers then use these basic insights to develop interventions assuming that the findings are universal^24,25^.

Although rare, psychological interventions designed with a particular cultural context in mind have shown promise in addressing a range of pressing issues^26–31^. For example, a study in China tried to encourage workers in a textile factory to throw their waste in trash cans instead of on the production floor^28^. The researchers found that placing printed images of golden coins (symbols of luck and good fortune in China) on the floor reduced littering, while fining people real money failed. Similarly, an intervention in Niger tried to encourage women to start their own businesses by paying them money or by changing community norms around female entrepreneurship^26^. The norms worked better per dollar spent than the cash transfers.

These studies highlight that economic activities are embedded within systems of local norms, prevalent morality and social networks^32–35^. Numerous studies across disciplines have demonstrated how socio-cultural factors interact with formal institutions to shape economic judgements and behaviour^36–46^.

In work contexts, cultural embeddedness is crucial for understanding the nature of obligations that exist between employers and their employees^47–49^, particularly because employment is both an economic and a social relationship^50^. Labour participation in the market economies of WEIRD cultures is said to be enacted in an impersonal and transactional manner, in accordance with the norms of market exchange^51–54^. Employees must work a specified amount of time or produce a specified amount of output to receive a specified amount of money from the employer. The scope of the mutual obligations is explicitly outlined in a formal contract^55,56^.

Outside of WEIRD cultures, work usually operates under norms of reciprocity exchange, which are marked by ‘contractual incompleteness’^51,57^. In such ‘relational contracts’^55,58^, people usually do not rely on the strict meanings of sentences specified in contracts. Instead, they interpret obligations on the basis of existing relationships and norms^59^. Even when there is a formal agreement, people express some expectations tacitly and obligations often go beyond the terms specified in the contract^58^.

Why might certain exchange norms and psychological contracts become more widespread in a society? According to some scholars, their prevalence is best explained by institutional factors^60^. For instance, in non-WEIRD cultures, legal mechanisms such as courts tend to be less effective. This makes formal enforcement of contracts difficult and encourages people to prioritize established and trusted economic partners^61–63^.

Other researchers emphasize the role of cultural dimensions, such as the degree of risk aversion^64^, interpersonal trust^65^ and particularly collectivism^66–68^. Collectivism is more common in non-WEIRD cultures and refers to the tendency to value duties and responsibilities to one’s in-group^41,69,70^. In collectivist cultures, people might by default assume reciprocal obligations and see formal contracts as at best nuisances and at worst threats to social harmony and harbingers of conflict^54,71^.

Of course, not all employment relations are governed solely by norms of market exchange in WEIRD cultures and norms of reciprocity exchange in non-WEIRD cultures. Rather, people from all cultures can apply different mental models of exchange norms and psychological contracts on the basis of the features of a situation, including in economic transactions^60,72–76^. Nevertheless, cultural and institutional influences can make people more likely to interpret a situation as either transactional or relational by increasing the salience of a mental frame that is ‘consistent with what is likely or what ought to happen between employee and employer’^56^.

We argue that different exchange norms and psychological contracts influence the marginal advantage of monetary relative to non-monetary incentives, which we call the ‘money advantage.’ Workers from WEIRD cultures, operating under market exchange norms, should prioritize explicit quid pro quo arrangements—establishing what needs to be done to receive a tangible benefit^56,77^—and feel less obligated to reciprocate beyond what is contractually prescribed and formally enforced^78,79^.

In contrast, workers from non-WEIRD cultures, guided by reciprocity exchange norms, might not stop at the contractual minimum, even if they receive no extra money for doing so. They might intuit that this minimum does not represent their employer’s real expectation^80^. Or they might reciprocate above the minimum prescribed by the contract to establish trust and earn loyalty for future interactions^77,81–83^. Therefore, monetary incentives might have a smaller marginal effect on workers using reciprocity exchange norms compared with those using market exchange norms.

There is little direct evidence about the relative influence of monetary and non-monetary incentives across cultures. Surveys have found that people from WEIRD cultures report valuing money less as a source of job motivation^84–87^. For instance, responding to a questionnaire about the desirability of different job characteristics, new IT recruits from the United States placed less emphasis on receiving monetary bonuses for completing projects than did their Chinese counterparts^85^.

However, these studies did not actually test the effectiveness of money. Explicit attitudes predict economic behaviour in some domains but not in others, and this attitude–behaviour link often depends on whether personal attitudes correspond to prevalent cultural norms and shared belief systems^88–90^. Norms shared by a network, culture or institution can direct individual economic behaviour regardless of personal attitudes through mechanisms such as internalization or sanctioning^91,92^.

People in the United States and other WEIRD cultures overemphasize the importance of extrinsic rewards, such as money, in their lay theories of what motivates others^93–97^. Some evidence suggests that people from WEIRD cultures agree more that self-interest is the primary determinant of others’ behaviour^67,98,99^.

Institutional designs often reflect such lay beliefs about what motivates others^85,91^. For example, a survey asked Citibank managers around the world about their employees’ primary motivation^99^. Compared with their colleagues in Asia and Latin America, managers in the United States were more likely to think that money and other extrinsic motivators were their employees’ primary motive. Given this belief among managers, it is perhaps unsurprising that across industries, incentive pay is more prevalent in WEIRD cultures^100–102^.

Similarly, the few experiments that directly compared the effectiveness of incentives across countries seem to suggest that money advantage is higher in WEIRD cultures^101,103–105^. For instance, in an experiment where the researchers tried paying students in the United States and China to get better test scores, money improved performance in the United States, but not in China^104^. Another study hired data-entry workers in three developing countries using contracts with a fixed salary or an incentive contingent on performance^101^. Performance pay increased effort the least in the country highest on collectivism.

In this paper, we systematically compared the effectiveness of money in the United States and the United Kingdom (two WEIRD cultures) vs China, India, Mexico and South Africa (four non-WEIRD cultures). In our experiments, workers received a fixed salary, a fixed salary plus a psychological intervention or a fixed salary plus an additional monetary incentive. We tested whether money is equally effective in WEIRD and non-WEIRD cultures. We measured effectiveness as effort and as cost-effectiveness of different incentives. In our last study, we randomly assigned people in India to take the study in English or Hindi to see whether this shifted people’s use of exchange norms and the motivational advantage of money.

## Study 1. Re-analysis of a large-scale experiment

In our first study, we re-analysed data from ref. ^8^, which gave Amazon Mechanical Turk (MTurk) workers a variety of incentives to perform a simple task. The task required participants to interchangeably click buttons ‘a’ and ‘b’ on their keyboards as many times as possible for 10 min. The researchers tested how much different incentives would increase the number of button presses.

All participants received a US$1.00 fee for participating in the experiment (all $ amounts henceforth are in US$). On top of that, some people received monetary incentives, such as an extra cent for every 100 clicks. The researchers compared money to many incentives from social psychology. For example, in one condition, the researchers gave participants a social reference point: “most participants perform well on the task, pressing over 2,000 times”. The results showed that (1) both pay-for-effort (monetary) and non-monetary incentives improved effort over the flat-fee condition, which included no additional incentives and (2) money outperformed non-monetary interventions by a large margin.

Fortunately for our purposes, many of the recruited MTurk workers were in India. This gave us a convenient opportunity to test cultural differences, namely, whether the money advantage was higher in the United States than in India. We excluded conditions with both a monetary and a psychological component (for example, loss aversion; more information is available in Methods, and Supplementary Table 9a,b). This left us with a sample of 5,526 participants from the United States (3,196 female, Mean_AgeCategory_ = 2.62, s.d._AgeCategory_ = 1.29) and 768 participants in India (247 female, Mean_AgeCategory_ = 2.41, s.d._AgeCategory_ = 1.02). Using this combined sample, we compared the effectiveness of pooled monetary incentives vs pooled psychological interventions.

### Results

**Fig. 1:**
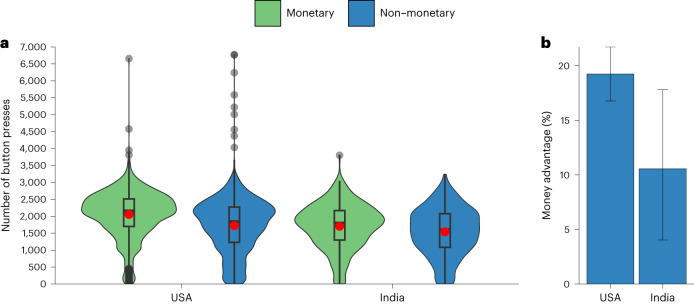
Study 1, pooled monetary vs pooled non-monetary conditions in the United States and India. Effects of pooled monetary incentives (green) and pooled non-monetary treatments (blue) in the United States (*N* = 5,526 participants on MTurk) and India (*N* = 768 participants on MTurk) from a previous study^8^. **a**, The central tendency and distribution of effort by incentive type and country. The black line within each box represents the median; the red dot shows the mean; upper and lower bounds show the third and first quartiles, respectively; whiskers represent 1.5× the interquartile range, with black dots showing observations outside of this range. The width of each violin corresponds to the frequency of observations at any given number of images rated on the *y* axis. The interaction between country and incentive type in a multiple linear regression model is statistically significant (*b* = 170.56, *t*(6,287) = 2.92, *P* = 0.004, 95% CI 55.91–285.21). **b**, The money advantage, that is, how much more effective money is than the pooled non-monetary treatments in each country. In **b**, error bars are bootstrapped 95% CIs for the mean relative difference in the number of button presses in the pooled monetary vs non-monetary treatments.

**Extended Data Fig. 1:**
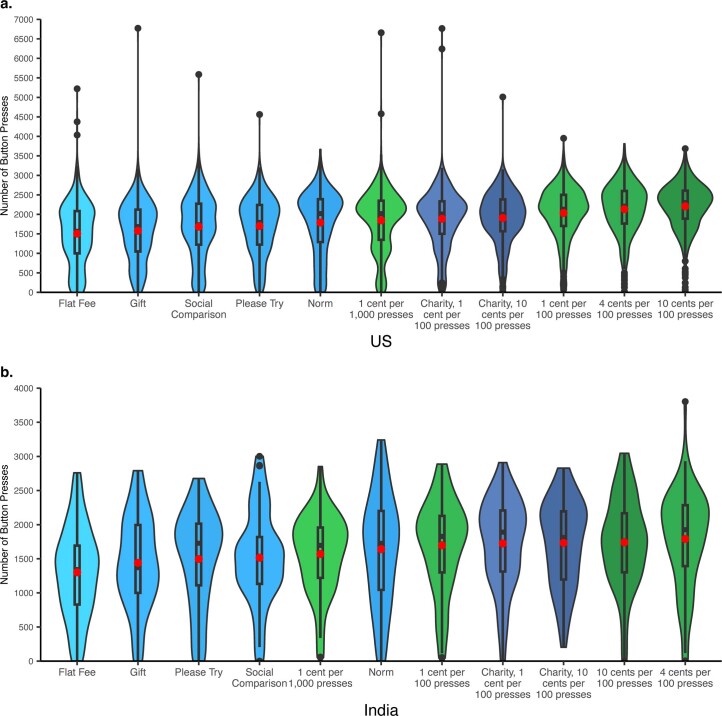
Study 1, Individual Conditions by Country. Effects of individual monetary incentives (green) and non-monetary treatments (blue) amongst participants in the US (*N =* 5,526 on MTurk) and India (*N* = 768 on MTurk) from a prior study^8^. Panels A (US) and B (India) show the central tendency and distribution of effort by incentive. Conditions within each panel (x-axis) are ordered from the lowest to highest mean effort in each country. The black line within each box represents a median, and the red dot shows a mean. Upper and lower bounds show the third and the first quartile, respectively. The whiskers represent the 1.5 times the interquartile range, with black points showing observations outside of this range. The width of each violin corresponds to the frequency of observations within each panel at any given number of button presses on the y-axis.

The difference in effort between monetary incentives and psychological interventions was larger in the United States than in India, as evidenced by an interaction between country and incentive type (unstandardized regression coefficient *b* = 170.56, *t*(6,287) = 2.92, *P* = 0.004, 95% confidence interval (CI) 55.91–285.21) (Fig. 1 and Supplementary Table 1). In these analyses, we controlled for age, gender and education. Main effects of country and incentive type for this and the following studies are reported in section ‘Main effects for reported regressions’ in Supplementary Information. Extended Data Fig. 1 shows effort in the individual incentive treatments by country.

The results from Study 1 suggest that the money advantage differs across cultures. Specifically, monetary incentives outperformed psychological interventions more in the United States than in India.

However, differences in technology could explain the results. For example, MTurk participants from the two countries could have understood the instructions differently. Furthermore, more participants in India could have completed the study on their phones or had slower internet connections, which could have limited how much they could ramp up their button-pressing, particularly in response to the monetary incentives.

## Studies 2a–c. The effectiveness of incentives in 5 cultures

In Study 2, we improved on the weaknesses of Study 1 by designing a new task. In our new task, participants saw images and assessed whether each image showed a building. Participants rated images one by one for a maximum of 10 min. In the monetary conditions, we paid participants more for rating more images. The bonus across Studies 2a–c ranged from 5 cents to 9 cents for every 10 images. In the non-monetary conditions, participants received the same pay regardless of how many images they rated. We asked comprehension questions to ensure that participants understood whether they would receive extra pay for rating more images.

We explicitly informed participants that they could quit the task without losing their base pay after rating 10 images. This gave a clear contractual minimal.

We also wanted to change the explicitly meaningless button-pressing task in Study 1 to be more like real-world work, which serves a purpose^5,106^. To this end, we told participants that we needed their help in training a machine-learning image-classification algorithm.

Since the non-monetary conditions had the same payout structure, in what follows, we first report the results comparing monetary vs pooled non-monetary conditions, including the fixed-salary (or flat-fee) condition. Then we compare the monetary incentive with each non-monetary treatment individually. In all regressions in Studies 2a–c, we control for gender, age and education. Regressions with additional controls, including Internet connection, are reported in ‘Additional controls’ in Supplementary Information.

### Study 2a. The United Kingdom and China

To test whether the results are generalizable to other cultures, we ran Study 2a in two new countries, the United Kingdom and China. To this end, we recruited 1,067 participants on Prolific in the United Kingdom (544 female, 12 non-binary, Mean_Age_ = 40.04, s.d._Age_ = 13.60) and 1,086 participants on social media through student networks at Hubei University in Wuhan, China (626 female, Mean_Age_ = 23.31, s.d._Age_ = 5.78). We compared the monetary condition (5 cents per 10 images) with the social norm and flat-fee conditions.

### Results

**Fig. 2:**
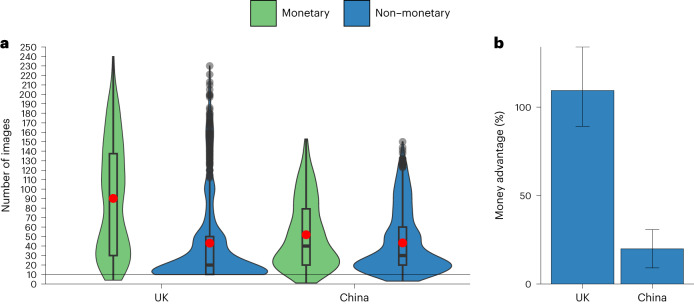
Study 2a, monetary vs pooled non-monetary conditions in the United Kingdom and China. Effects of a monetary incentive (green) and pooled non-monetary treatments (flat fee and social norm; blue) in the United Kingdom (*N* = 1,067 participants recruited on Prolific) and China (*N* = 1,086 participants recruited on social media). **a**, The central tendency and distribution of effort by incentive type and country. The black line within each box represents the median and the red dot shows the mean; upper and lower bounds show the third and first quartiles, respectively; whiskers represent 1.5× the interquartile range, with black dots showing observations outside of this range. The width of each violin corresponds to the frequency of observations at any given number of images rated on the *y* axis. The interaction between country and incentive type in a multiple linear regression model is statistically significant (*b* = 38.40*, t*(2,145) = 9.65, *P* < 0.001, 95% CI 30.59–46.20). **b**, The money advantage, that is, how much more effective money is than the pooled non-monetary treatments in each country. In **b**, error bars are bootstrapped 95% CIs for the mean relative difference in the number of images rated in the monetary vs pooled non-monetary conditions.

The difference in the effectiveness between monetary and non-monetary incentives was larger in the United Kingdom than in China, as evidenced by the interaction between incentive and culture (*b* = 38.40*, t*(2,145) = 9.65, *P* < 0.001, 95% CI 30.59–46.20) (Fig. 2a and Supplementary Table 2). In China, extra pay increased effort by 19.9% over the two non-monetary conditions. In the United Kingdom, money increased effort by 109.5% (Fig. 2b).

**Extended Data Fig. 2:**
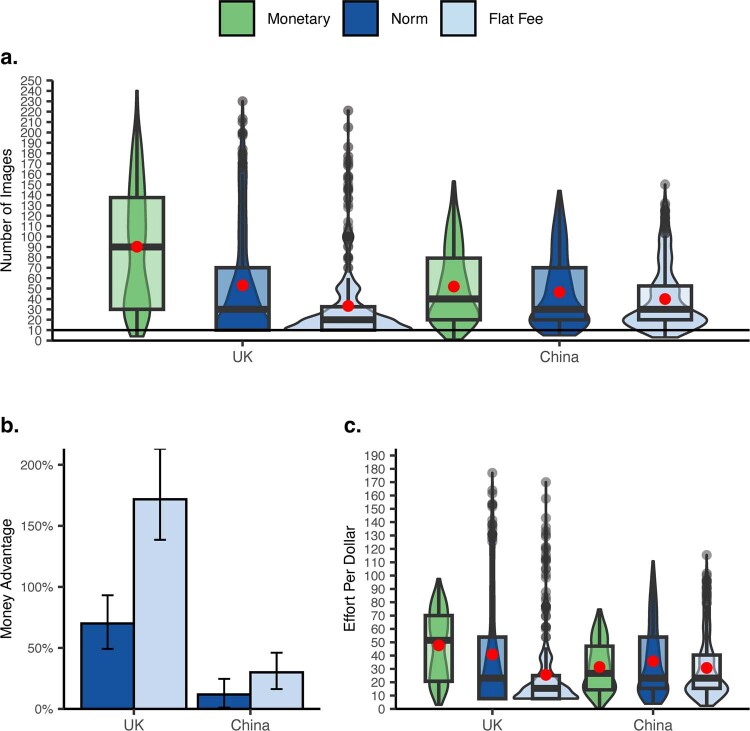
Study 2a, Monetary Versus Individual Non-Monetary Conditions in the UK and China. Effects of the three individual conditions: monetary (green), social norm (dark blue) and flat fee (light blue) in the UK (*N* = 1,067 recruited on Prolific) and China (*N* = 1,086 recruited on social media). Panel A shows the central tendency and distribution of effort by incentive type and country. The black line within each box represents a median, and the red dot shows a mean. Upper and lower bounds show the third and the first quartile, respectively. The whiskers represent the 1.5 times the interquartile range, with black points showing observations outside of this range. The width of each violin corresponds to the frequency of observations at any given number of images rated on the y-axis. Panel B shows the money advantage—that is, how much more effective money is than each of the two non-monetary treatments in each country. Panel C shows the central tendency and distribution of cost-effectiveness (effort per dollar spent) by incentive type and country. Graph elements are analogous to those in Panel A, with the width of each violin corresponding to the frequency of observations at any given level of cost-effectiveness (effort per dollar spent) rated on the y-axis. The results for cost-effectiveness are summarized in the main text. In Panel B, error bars are bootstrapped 95% CIs for the mean relative difference in the number of images in the monetary and each of the non-monetary conditions.

The money advantage over each of the two non-monetary conditions (flat fee and norm) was significantly larger in the United Kingdom than in China (*b* = −44.88, *t*(2,143) = −9.82, *P* < 0.001, 95% CI −53.84 to −35.91 for the flat-fee condition; and *b* = −31.55, *t*(2,143) = −6.96, *P* < 0.001, 95% CI −40.44 to −22.67 for the norm condition) (Extended Data Fig. 2a and Supplementary Table 7).

In China, the monetary condition was significantly less cost-effective than the norm condition (two-sided Welch’s *t*(681.11) = −2.54, *P* = 0.011, *P*_Bonf_ = 0.045, Mean_difference_ = −4.28, Cohen’s *d* = −0.19, 95% CI −7.58 to −0.97) and did not significantly differ in cost-effectiveness from the flat-fee condition (two-sided Welch’s *t*(658.54) = 0.45, *P* = 0.653, *P*_Bonf_ = 1.000, Mean_difference_ = 0.73, *d* = 0.03, 95% CI −2.45 to 3.90) (Extended Data Fig. 2c). In the United Kingdom, the monetary condition was significantly more cost-effective than each of the two non-monetary conditions: monetary and norm, two-sided Welch’s *t*(574.32) = 2.70, *P* = 0.007, *P*_Bonf_ = 0.029, Mean_difference_ = 6.84, *d* = 0.20, 95% CI 1.86 to 11.82; monetary and flat fee, two-sided Welch’s *t*(692.87) = 10.88, *P* < 0.001, *P*_Bonf_ < 0.001, Mean_difference_ = 22.11, *d* = 0.81, 95% CI 18.12–26.10.

### Study 2b. Adjusting the pay to reflect the real value of money in Mexico and the United States

We followed up on Study 2a to make up for two shortcomings of the design. For one, Study 2a compared people on two different platforms (Prolific in the United Kingdom and social media in China). Because Prolific is a work platform, it could explain the difference in the money advantage without reference to culture. Second, we paid participants in the two countries the same amount of money. However, a dollar goes further in China than in the United Kingdom. To compensate for these shortcomings, in Studies 2b and 2c, we recruited workers only on crowdsourcing platforms and adjusted for local purchasing power.

In Study 2b, we extended the sample of non-WEIRD cultures to Mexico and again compared the monetary condition with the social norm and flat-fee conditions. We recruited a Prolific sample in Mexico (*N* = 1,053; 536 female, 26 non-binary, Mean_Age_ = 24.51, s.d._Age_ = 5.43) and two Prolific samples in the United States. In one of these US samples (*N* = 1,098; 652 female, 24 non-binary, Mean_Age_ = 37.28, s.d._Age_ = 13.77), we paid participants the same amount of money as in Mexico. In the other US sample (*N* = 1,122; 674 female, 25 non-binary, Mean_Age_ = 36.13, s.d._Age_ = 13.31), we increased the pay so that it became subjectively equivalent to the amount received by the participants in Mexico. To this end, we used a common economics procedure to establish subjective pay equivalence^107^ (see Methods). Participants in the increased-pay sample in the United States received $1.56 as base pay for their participation (compared with $1.30 in the other two samples) and a piece-rate of 6 cents (compared with 5 cents).

### Results

**Fig. 3:**
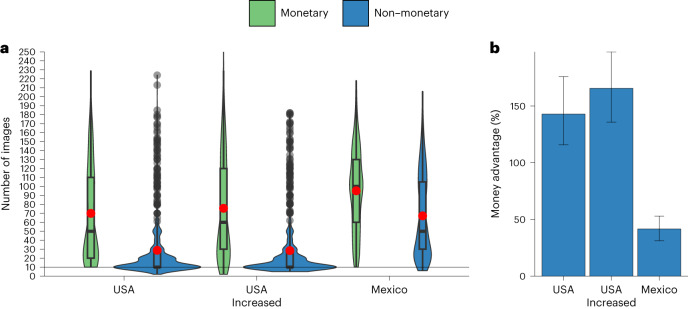
Study 2b, monetary vs pooled non-monetary conditions in the United States and Mexico. Effects of a monetary incentive (green) and pooled non-monetary treatments (flat fee and social norm; blue) in Mexico (*N* = 1,053 participants recruited on Prolific) and two samples in the United States: one with the same nominal pay (*N* = 1,098 on Prolific) as in Mexico and one with the same subjective^107^ pay (*N* = 1,122 participants recruited on Prolific) as in Mexico. **a**, The central tendency and distribution of effort by incentive type and country. The black line within each box represents the median and the red dot shows the mean; upper and lower bounds show the third and first quartiles, respectively; whiskers represent 1.5× the interquartile range, with black dots showing observations outside of this range. The width of each violin corresponds to the frequency of observations at any given number of images rated on the *y* axis. The interaction between country and incentive type in a multiple linear regression model is statistically significant (*b* = 13.93*, t*(2,143) = 3.42, *P* < 0.001, 95% CI 5.94–21.92 for the comparison between Mexico and the US sample with the same nominal pay; *b* = 19.28, *t*(2,167) = 4.71, *P* < 0.001, 95% CI 11.26–27.30 for the comparison between Mexico and the US sample with the same subjective pay). **b**, The money advantage, that is, how much more effective money is than the pooled non-monetary treatments in each sample. In **b**, error bars are bootstrapped 95% CIs for the mean relative difference in the number of images rated in the monetary vs pooled non-monetary conditions.

The money advantage was larger in the United States than in Mexico, regardless of whether we compared Mexico to the US sample with the same nominal pay (*b* = 13.93*, t*(2,143) = 3.42, *P* < 0.001, 95% CI 5.94–21.92), or that with the same subjective pay (*b* = 19.28, *t*(2,167) = 4.71, *P* < 0.001, 95% CI 11.26–27.30) (Fig. 3a and Supplementary Table 3). Stated differently, the effectiveness of monetary and non-monetary motivators was closer to one another in Mexico than in the United States. In the United States, money increased effort by 142.9% over the two pooled non-monetary conditions in the sample with the same nominal pay and by 165.6% in the sample with the same subjective pay (Fig. 3b). In Mexico, the difference was 41.6%.

**Extended Data Fig. 3:**
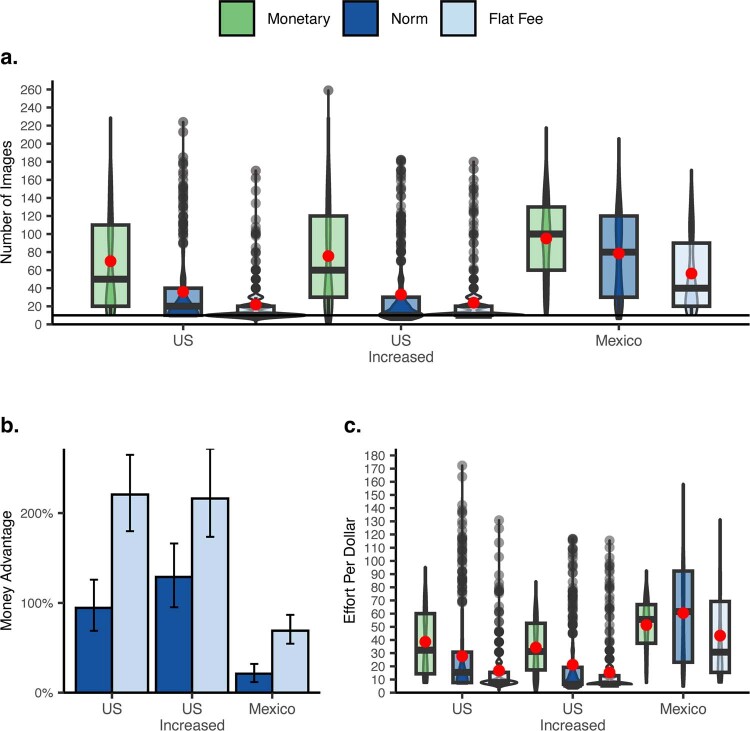
Study 2b, Monetary Versus Individual Non-Monetary Conditions in the US and Mexico. Effects of the three individual conditions: monetary (green), social norm (dark blue) and flat fee (light blue) in Mexico (*N* = 1,053 recruited on Prolific) and two samples in the US: one with the same nominal pay (*N* = 1,098 recruited on Prolific) as in Mexico and the other with the same subjective^107^ pay (*N* = 1,122 recruited on Prolific) as in Mexico. Panel A shows the central tendency and distribution of effort by incentive type and country. The black line within each box represents a median, and the red dot shows a mean. Upper and lower bounds show the third and the first quartile, respectively. The whiskers represent the 1.5 times the interquartile range, with black points showing observations outside of this range. The width of each violin corresponds to the frequency of observations at any given number of images rated on the y-axis. Panel B shows the money advantage—that is, how much more effective money is than each of the two non-monetary treatments in each sample. Panel C shows the central tendency and distribution of the cost-effectiveness (effort per dollar spent) by incentive type and country. Graph elements are analogous to those in Panel A, with the width of each violin corresponding to the frequency of observations at any given level of cost-effectiveness (effort per dollar spent) rated on the y-axis. The results for cost-effectiveness are summarized in the main text. In Panel B, error bars are bootstrapped 95% CIs for the mean relative difference in the number of images in the monetary and each of the non-monetary conditions.

Next, we analysed individual conditions. The interaction between culture and incentive was statistically significant. The money advantage was larger in the United States than in Mexico for the flat-fee condition (*b* = −10.32, *t*(2,141) = −2.24, *P* = 0.025, 95% CI −19.37 to −1.27) and for the norm condition (*b* = −17.69, *t*(2,141) = −3.81, *P* < 0.001, 95% CI −26.80 to −8.58) (Extended Data Fig. 3a and Supplementary Table 8). We found the same pattern in our comparison of Mexico and the US sample with the same subjective pay: money was relatively more effective in the United States than in Mexico when compared with the flat-fee condition (*b* = −13.41, *t*(2,165) = −2.88, *P* = 0.004, 95% CI −22.55 to −4.27) and the norm condition (*b* = −25.55, *t*(2,165) = −5.47, *P* < 0.001, 95% CI −34.72 to −16.38).

Finally, we analysed the amount of effort per dollar spent for each of the individual incentive conditions. In Mexico, the monetary condition was significantly less cost-effective than the norm condition (two-sided Welch’s *t*(527.85) = −3.90, *P* < 0.001, *P*_Bonf_ < 0.001, Mean_difference_ = −9.03, *d* = −0.30, 95% CI −13.58 to −4.49) but more cost-effective than the flat-fee condition (two-sided Welch’s *t*(605.56) = 4.00, *P* < 0.001, *P*_Bonf_ < 0.001, Mean_difference_ = 8.07, *d* = 0.30, 95% CI 4.11–12.03) (Extended Data Fig. 3c).

In both samples in the United States, money was more cost-effective than the norm condition (same nominal pay: two-sided Welch’s *t*(671.05) = 5.09, *P* < 0.001, *P*_Bonf_ < 0.001, Mean_difference_ = 10.97, *d* = 0.38, 95% CI 6.74–15.20; increased pay: two-sided Welch’s *t*(721.26) = 7.76, *P* < 0.001, *P*_Bonf_ < 0.001, Mean_difference_ = 13.13, *d* = 0.57, 95% CI 9.81–16.46) and the flat-fee condition (same nominal pay: two-sided Welch’s *t*(665.00) = 13.70, *P* < 0.001, *P*_Bonf_ < 0.001, Mean_difference_ = 21.85, *d* = 1.01, 95% CI 18.72–24.99; increased pay: two-sided Welch’s *t*(742.17) = 12.96, *P* < 0.001, *P*_Bonf_ < 0.001, Mean_difference_ = 18.97, *d* = 0.95, 95% CI 16.10–21.85). Thus, consistent with the findings in Study 2a, we found that a monetary incentive is less cost-effective than a non-monetary intervention (social norm) in a non-WEIRD culture (Mexico), but not in a WEIRD culture (the United States).

### Study 2c. Pre-registered replication in the United States and South Africa

In Study 2c, we made three improvements over Study 2b. First, we pre-registered the hypothesis and analysis. Second, we extended the sample to another non-WEIRD culture. We recruited Prolific workers in the United States (two samples, *N* = 662 each; 318 female, 20 non-binary, Mean_Age_ = 36.91, s.d._Age_ = 12.62 in the sample with the same nominal pay; 323 female, 17 non-binary, Mean_Age_ = 36.27, s.d._Age_ = 12.52 in the sample with the same subjective pay) and South Africa (*N* = 649; 316 female, 6 non-binary, Mean_Age_ = 28.29, s.d._Age_ = 7.45). South Africa is an interesting test case because, although it scores lower on individualism than the United States, it is more individualistic than China or India^108^. Individualism is often contrasted with collectivism and refers to the tendency to prioritize individual goals and achievement^41,69,70^. South Africa’s English-speaking population tends to be particularly individualist^109,110^. All participants in South Africa completed the study in English and reported speaking it fluently.

Third, we tested a wider range of psychological interventions. We tried to ‘stress test’ our findings by choosing non-monetary interventions we suspected might be particularly effective in WEIRD cultures. In one condition, we told participants they were competing with other participants, since some researchers see competition as a facet of individualism^111,112^. In another condition, we incentivized people with donations to organized charities, which receive more contributions in WEIRD compared with non-WEIRD cultures (even controlling for income)^113,114^. Given these stress tests, we anticipated smaller effect sizes than in Studies 2a and 2b. We compared these two conditions (competition and charity) with the monetary condition.

As in Study 2b, we ran some US participants with the same pay as offered to the participants in South Africa. Other US participants worked for pay adjusted to reflect local differences in purchasing power, using a procedure analogous to the one in Study 2b^107^. In the US sample with the same subjective pay, we increased the base pay from $1.30 to $2.25 in all conditions, as well as the piece-rate from 5 to 9 cents per 10 images in the monetary condition.

We pre-registered pooled analyses because we did not have sufficient power to compare individual conditions. We report the comparisons between the monetary and each of the non-monetary conditions in ‘Mean effort and cost-effectiveness in individual conditions in Study 2c’ in Supplementary Information, but readers should be aware that the statistical power for these comparisons is lower.

### Results

**Fig. 4:**
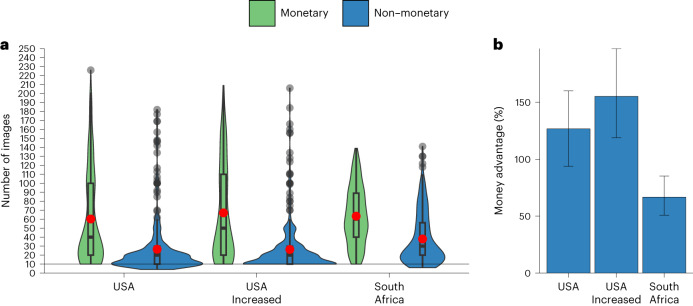
Study 2c, monetary vs pooled non-monetary conditions in the United States and South Africa. Effects of a monetary incentive (green) and pooled non-monetary treatments (competition and charity; blue) in South Africa (*N* = 649 participants on Prolific) and two samples in the United States: one with the same nominal pay (*N* = 662 on Prolific) as in South Africa and one with the same subjective^107^ pay (*N* = 662 participants on Prolific) as in South Africa. **a**, The central tendency and distribution of effort by incentive type and country. The black line within each box represents the median and the red dot shows the mean; upper and lower bounds show the third and first quartiles, respectively; whiskers represent 1.5× the interquartile range, with black dots showing observations outside of this range. The width of each violin corresponds to the frequency of observations at any given number of images rated on the *y* axis. The interaction between country and incentive type in a multiple linear regression model is statistically significant (*b* = 7.98*, t*(1,303) = 1.97, *P* = 0.049, 95% CI 0.03–15.92 for the comparison between South Africa and the US sample with the same nominal pay; *b* = 15.40, *t*(1,303) = 3.74, *P* < 0.001, 95% CI 7.33–23.48 for the comparison between South Africa and the US sample with the same subjective pay). **b**, The money advantage, that is, how much more effective money is than the pooled non-monetary treatments in each sample. In **b**, error bars are bootstrapped 95% CIs for the mean relative difference in the number of images rated in the monetary vs pooled non-monetary conditions.

The difference in the effectiveness between monetary and non-monetary conditions was larger in the United States than in South Africa, both when we compared the sample in South Africa to the US sample with the same nominal pay (*b* = 7.98*, t*(1,303) = 1.97, *P* = 0.049, 95% CI 0.03–15.92) and to the US sample with the same subjective pay (*b* = 15.40, *t*(1,303) = 3.74, *P* < 0.001, 95% CI 7.33–23.48) (Fig. 4a and Supplementary Table 4). Additional money increased effort by 66.7% in South Africa, but by 126.6% in the US sample with the same nominal pay and by 155.2% in the US sample with increased pay (Fig. 4b).

## Studies 3a,b. Comparing psychological incentives and minimal pay

Studies 2a–c extended the findings from Study 1 to four other cultures and to a more meaningful task. The money advantage was higher in WEIRD than in non-WEIRD cultures both when the contractual minimum was left ambiguous (Study 1) and when it was made explicit (Studies 2a–c). Cultural differences persisted after we adjusted the pay to reflect local purchasing power (Studies 2b and 2c).

In Study 3a, we pushed the boundaries of just how little extra money beyond the base pay it would take to spur extra effort. The bonuses we paid in Studies 2a–c were not large, but they were above average for crowdsourcing sites. Surveys have found that crowdsourcing workers average $3.31 per hour, and the pay is even lower for non-Western workers^115,116^. Our studies maxed out at 10 min, which would work out to 55 cents at these sites’ average wage. In the monetary conditions in Studies 2a–c, participants could earn more than that from the piece-rates alone (and the base pay made the earnings even better). Since the piece-rates were above average, it may not be surprising that monetary incentives would be more motivating than psychological interventions.

This made us wonder whether it was the actual pragmatic value of the money that motivated people or whether money holds a symbolic value beyond the actual usefulness of the amount. To test this, we changed the piece-rates in Study 3a to negligible amounts.

### Study 3a. Lowering the piece-rate in India and the United States

In Study 3a, we lowered the bonus to a penny for every 20 images completed. We compared this minimal pay condition to a social norm intervention in samples in India (*N* = 352 recruited on MTurk; 83 female, Mean_Age_ = 35.79, s.d._Age_ = 8.73) and the United States (*N* = 382 recruited on Prolific; 197 female, 14 non-binary, Mean_Age_ = 37.76, s.d._Age_ = 13.99). We pre-registered the hypothesis, methods and analysis. As in the previous studies, we analysed the data using regressions controlling for age, gender and education.

### Results

**Fig. 5:**
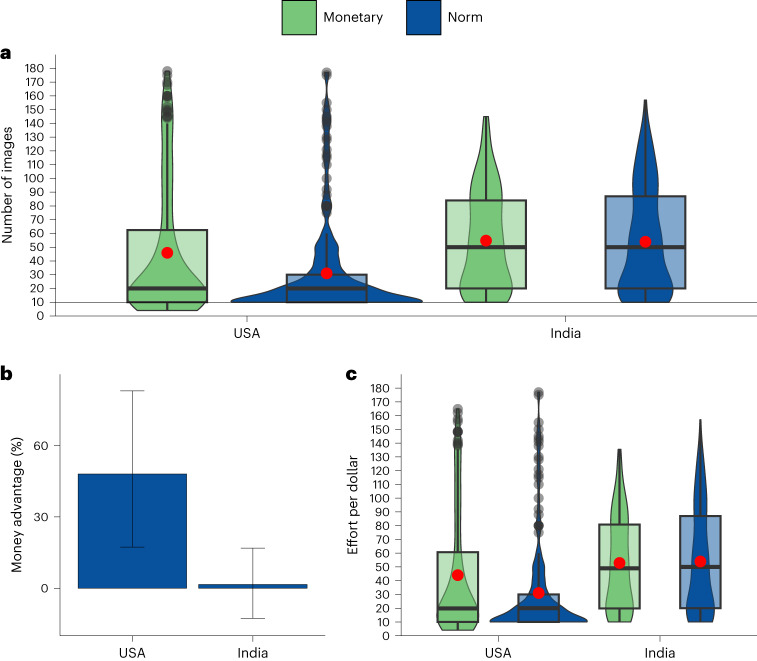
Study 3a, norm vs minimal pay condition in India and the United States. Effects of a minimal monetary incentive (of 1 cent per 20 image ratings; green) and a social norm condition (blue) in the United States (*N* = 382 participants recruited on Prolific) and India (*N* = 352 participants recruited on MTurk). **a**, The central tendency and distribution of effort by incentive and country. The black line within each box represents the median and the red dot shows the mean; upper and lower bounds show the third and first quartiles, respectively; whiskers represent 1.5× the interquartile range, with black dots showing observations outside of this range. The width of each violin corresponds to the frequency of observations at any given number of images rated on the *y* axis. The interaction between country and incentive in a multiple linear regression model is statistically significant (*b* = 13.77, *t*(726) = 2.31, *P* = 0.021, 95% CI 2.09–25.45). **b**. The money advantage, that is, how much more effective the minimal incentive is compared to the social norm condition in each country. **c**, The central tendency and distribution of cost-effectiveness (effort per dollar spent) of each incentive by country. Graph elements are analogous to those in **a**, with the width of each violin corresponding to the frequency of observations at any given level of cost-effectiveness (effort per dollar spent) rated on the *y* axis. Minimal monetary incentive is significantly more cost-effective than the social norm condition in the United States (two-sided Welch’s *t*(365.20) = 3.14, *P* = 0.002, *P*_Bonf_ = 0.004, Mean_difference_ = 13.00, *d* = 0.32, 95% CI 4.86–21.15) but not in India (two-sided Welch’s *t*(345.72) = −0.27, *P* = 0.785, *P*_Bonf_ = 1.000, Mean_difference_ = −1.06, *d* = −0.03, 95% CI −8.73 to 6.60). In **b**, error bars are bootstrapped 95% CIs for the mean relative difference in the number of images rated in the minimal-monetary-incentive vs social norm condition.

The money advantage was larger in the United States than in India, as evidenced by a statistically significant interaction between cultures (United States, India) and incentives (norm, monetary) (*b* = 13.77, *t*(726) = 2.31, *P* = 0.021, 95% CI 2.09–25.45) (Fig. 5a and Supplementary Table 5). A tiny monetary incentive, when compared to the social norm condition, increased effort by just 1.6% in India but by 48.1% in the United States (Fig. 5b).

In India, paying 1 additional cent per 20 images rated was not significantly more cost-effective than emphasizing a descriptive norm (two-sided Welch’s *t*(345.72) = −0.27, *P* = 0.785, *P*_Bonf_ = 1.000, Mean_difference_ = −1.06, *d* = −0.03, 95% CI −8.73 to 6.60) (Fig. 5c). In the United States, minimal pay was significantly more cost-effective than the social norm condition (two-sided Welch’s *t*(365.20) = 3.14, *P* = 0.002, *P*_Bonf_ = 0.004, Mean_difference_ = 13.00, *d* = 0.32, 95% CI 4.86–21.15).

**Extended Data Fig. 4:**
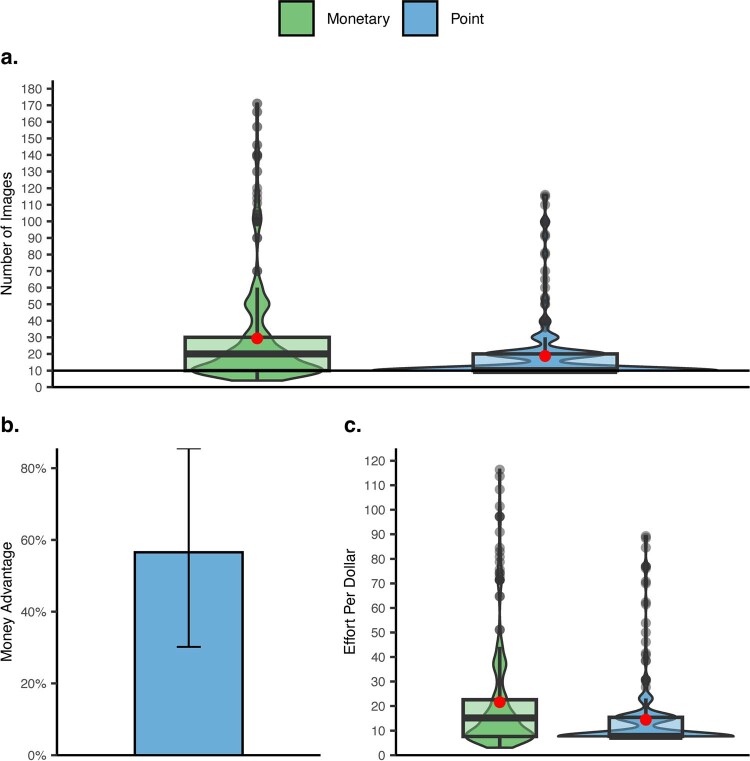
Supplementary Study 3b, Minimal Pay Versus Points (Gamification) Condition in the US. Effects of the minimal monetary incentive (1 cent per rating 10 images; in green) and a non-monetary gamification treatment (1 extra point per rating 10 images; in blue) in the US (*N* = 537 on Prolific). Panel A shows the central tendency and distribution of effort by incentive condition. The black line within each box represents a median; the red dot shows a mean. Upper and lower bounds show the third and the first quartile, respectively. The whiskers represent the 1.5 times the interquartile range, with black points showing observations outside of this range. The width of each violin corresponds to the frequency of observations at any given number of images rated on the y-axis. The difference in the number of images rated in the two conditions is statistically significant, Welch’s *t*(446.98) = 4.67, *P* < 0.001, *Mean*_difference_ = 10.61, *d* = 0.40, 95% CI 6.15 to 15.08. Panel B shows the money advantage—that is, how much more effective the minimal monetary incentive is compared to the gamification condition. Panel C shows the central tendency and distribution of cost-effectiveness (effort per dollar spent) of each incentive. Graph elements are analogous to those in Panel A, with the width of each violin corresponding to the frequency of observations at any given level of cost-effectiveness (effort per dollar spent) rated on the y-axis. The minimal monetary incentive is more cost-effective than the gamification treatment, Welch’s *t*(471.93) = 4.37, *P* < 0.001, *Mean*_difference_ = 7.15, *d* = 0.38, 95% CI 3.93 to 10.36. In Panel B, the error bar is a bootstrapped 95% CI for the mean relative difference in the number of images rated in the minimal-monetary-incentive versus social-norm condition.

Therefore, Study 3a showed that relative to a psychological intervention, even a minimal monetary incentive was more motivating in a WEIRD compared with a non-WEIRD culture. In the pre-registered Supplementary Study 3b, we further found that gamification^117–119^—triggering the sense of competitive games or accumulating non-financial rewards such as points in a video game—could not explain the effectiveness of minimal pay in the United States (Extended Data Fig. 4).

## Study 4. Randomly assigning cultural frames through language

In Study 4, we conducted a lab-in-the-field experiment to overcome the problem of causality. In interpreting the results of our previous studies, we attributed the differences to culture, but we did not randomly assign culture. To get at this issue, we randomly assigned bilingual people in India (*N* = 2,065; 286 female, 2 non-binary, Mean_Age_ = 24.87, s.d._Age_ = 4.90) to complete the study in Hindi or in English.

Previous studies have found that having bilingual people switch languages activates a discrete set of social and moral norms in the culture associated with each language^120–124^. For example, one study surveyed managers in Hong Kong in Chinese or English^122^. When surveyed in English, the surveyed managers endorsed values more similar to those espoused by American managers. For instance, they rated conformity and tradition as less important, while they rated achievement and hedonism as more important.

We recruited participants through a post on the Facebook group QMaths. This group has over 280,000 members interested in preparing for competitive exams for jobs in sectors ranging from banking to railways.

We compared how many images participants completed when given a monetary reward vs a psychological intervention (social norm). In addition, we asked questions to explore the mechanism behind the cultural differences. The seven exploratory variables asked about participants’ motivation for and perception of the task, such as whether they enjoyed completing the task and whether they completed it only for money.

### Results

**Fig. 6:**
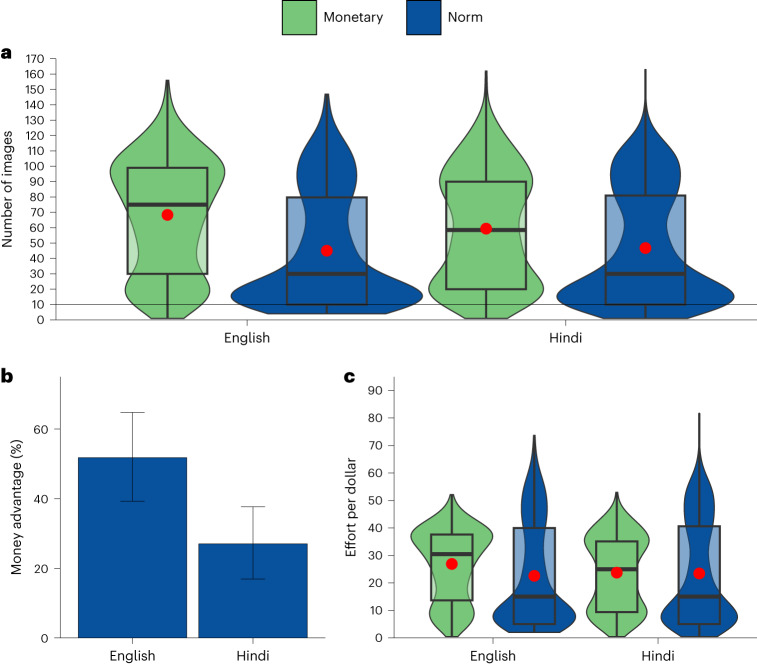
Study 4, norm vs monetary condition in India, by language prime (English or Hindi). Effects of a monetary incentive (green) and a social norm treatment (blue) in India (*N* = 2,065 participants recruited on Facebook), by assigned language (English or Hindi). **a**, The central tendency and distribution of effort by language and incentive conditions. The black line within each box represents the median and the red dot shows the mean; upper and lower bounds show the third and first quartiles, respectively; whiskers represent 1.5× the interquartile range, with black dots showing observations outside of this range. The width of each violin corresponds to the frequency of observations at any given number of images rated on the *y* axis. The interaction between language and incentive in a multiple linear regression model is statistically significant (*b* = 10.69, *t*(2,061) = 3.31, *P* = 0.001, 95% CI 4.35–17.02). **b**, The money advantage, that is, how much more effective the monetary condition is compared to the social norm condition. **c**, The central tendency and distribution of the cost-effectiveness (effort per dollar spent) by language and incentive. Graph elements are analogous to those in **a**, with the width of each violin corresponding to the frequency of observations at any given level of cost-effectiveness (effort per dollar spent) rated on the *y* axis. The monetary incentive is more cost-effective than the social norm condition in English (two-sided Welch’s *t*(919.21) = 4.37, *P* < 0.001, *P*_Bonf_ < 0.001, Mean_difference_ = 4.34, *d* = 0.27, 95% CI 2.39–6.28), but the two incentives do not significantly differ in their cost-effectiveness in Hindi (two-sided Welch’s *t*(915.62) = 0.30, *P* = 0.761, *P*_Bonf_ = 1.000, Mean_difference_ = 0.30, *d* = 0.02, 95% CI −1.64 to 2.24). In **b**, error bars are bootstrapped 95% CIs for the mean relative difference in the number of images rated in the norm vs monetary condition.

The money advantage was larger when participants took the study in English than in Hindi, as evidenced by an interaction between language and incentive type (*b* = 10.69, *t*(2,061) = 3.31, *P* = 0.001, 95% CI 4.35–17.02) (Fig. 6a and Supplementary Table 6). The difference in effort between the monetary and the norm condition was 51.8% in English and 27.0% in Hindi (Fig. 6b).

In our analyses on cost-effectiveness, we found that the monetary incentive was more cost-effective than the social norm in English (two-sided Welch’s *t*(919.21) = 4.37, *P* < 0.001, *P*_Bonf_ < 0.001, Mean_difference_ = 4.34, *d* = 0.27, 95% CI 2.39–6.28) (Fig. 6c). By contrast, in Hindi, the two conditions did not significantly differ in their cost-effectiveness (two-sided Welch’s *t*(915.62) = 0.30, *P* = 0.761, *P*_Bonf_ = 1.000, Mean_difference_ = 0.30, *d* = 0.02, 95% CI −1.64 to 2.24).

Next, we analysed whether the cultural prime influenced motivations. Language produced a statistically significant difference in one of seven items (with Bonferroni corrections). Participants were more likely to say that they completed the task only for money in English than in Hindi (*b* = 0.40, *t*(2,017) = 3.49, *P* = 0.001, *P*_Bonf_ = 0.004, 95% CI 0.18–0.63, 99.3% CI 0.09–0.71), which suggests that English activated a more transactional mental frame across incentive treatments. We report further details on the motivation questions in Supplementary Tables 14, 15a,b and 16, and Supplementary Fig. 3.

Thus, in Study 4 we found that English increased the money advantage. However, we recognize that using language to switch cultural frames is not reducible to the simple claim that the Hindi condition ‘randomly assigns’ Indian culture, while the English condition ‘randomly assigns’ United States (or United Kingdom, or WEIRD) culture. The differential set of associations activated by the two languages is likely to be considerably more complex^120,125^.

For instance, English might activate concepts related to work, professional life or achievement. This may be particularly true given that English is the primary language used for higher education in India and is often associated with status and prestige^126,127^. Similarly, people who completed the study in English may have implicitly compared themselves to native English speakers, which could have led to more upward social comparisons. These are empirically testable conjectures worthy of unpacking in future studies. However, it is worth remembering that professionalism, drive for prestige and social comparison are all part of culture too.

## Discussion

How effective are different incentives in motivating effort? The data here suggest that the answer depends on culture. The motivating power of money over psychological interventions was stronger in the United States and the United Kingdom than in China, India, Mexico and South Africa. In China and Mexico, a social norm intervention was more cost-effective than a monetary incentive. In our last study, we found that randomly assigning people in India to take the study in English (compared with in Hindi) increased the money advantage. Participants who took the study in English were also more likely to report that they completed the task ‘only for money’, which suggests that they thought of their work more in accordance with the transactional norms of market exchange.

We interpret our results as reflecting cultural differences in exchange norms and psychological contracts. However, one can argue that our findings show that people in WEIRD cultures are closer to the model of Homo economicus, particularly in its narrow reading that includes the ‘selfishness axiom’^128^, or the assumption that ‘individuals seek to maximize their own material gains…and expect others to do the same’^40^. If maximizing material gains is the sole goal, our participants should work only when it pays. Many Americans did just that, living up to Benjamin Franklin’s dictum that “time is money.”

The most marked difference was observed in Study 2b. There, over half of the American participants quit the task as soon as they could do so without losing the base pay when there was no monetary incentive to continue. In Mexico, over 90% of participants continued even when they knew that extra effort would not result in more pay (see ‘Probability of quitting the task at the first opportunity in Studies 2a–4’ in Supplementary Information).

We do not see the Homo economicus interpretation as contradictory to our focus on transactional contracts and exchange norms. Both make the same prediction regarding effort in the non-monetary conditions^67,129^. However, Homo economicus has become somewhat of a strawman^130^, particularly since most economists agree that rational agents can derive utility not solely from material gains, but also from moral and reputational considerations^38,131^. Both working above the contractual minimum and quitting in the absence of monetary incentives can be seen as rational utility-maximizing actions. Therefore, we neither interpret the behaviour of non-WEIRD participants in our studies as ‘irrational’, nor do we evaluate the behaviour of WEIRD participants as demonstrating an obsession with money. Instead, we theorize that transactional mentality is a continuum, whereby people in some cultures are more likely to perceive work as governed by explicit contracts and inherently involving monetary remuneration^132,133^.

Our experiments have limitations because they stripped out several elements of work in the real world. We can only theorize whether the same cultural differences in the money advantage would arise in different tasks. For one, our tasks gave all workers a base salary, meaning that no one worked solely for commission. In addition, everyone worked alone, without the potential influence of coworkers. We also randomly assigned incentives to workers, but some workers in the real world can choose between jobs on the basis of how much those jobs pay and how much fulfilment they give^134^. Moreover, while our experiments focused on the effectiveness of incentives in terms of motivating effort, it is not the sole metric relevant to evaluating their overall value^135^. For instance, prioritizing performance pay can lead to negative consequences such as higher levels of stress^136^ and lower quality of non-work relationships^137^. Therefore, in future research, it is crucial to assess various incentives on other factors, such as people’s sense of overall well-being.

The findings of our study also raise thorny questions about pay. People could use our data to justify paying people in non-WEIRD cultures less. The extent to which employers across different countries already take advantage of these tendencies deserves further research. While performance pay has been found to be more frequent in WEIRD countries, employees in non-WEIRD countries might receive more of other types of compensation, such flexible benefits or group-based performance rewards^100,138^. This having been said, we do not believe that our findings justify exploitation: non-monetary incentives cannot and should not be seen as a substitute for paying a fair wage. In our design, everyone received a base pay (a ‘salary’), which was probably the first-order motivator. On MTurk and Prolific, participants would probably not start tasks in the first place if they were not paid^84^. Instead, we interpret our results as suggesting that, given an adequate salary, workers from non-WEIRD cultures are less incentivized by further pay-for-effort incentives as compared with free psychological motivators. We discuss this further in the ‘Additional discussion’ section in Supplementary Information.

**Extended Data Fig. 5:**
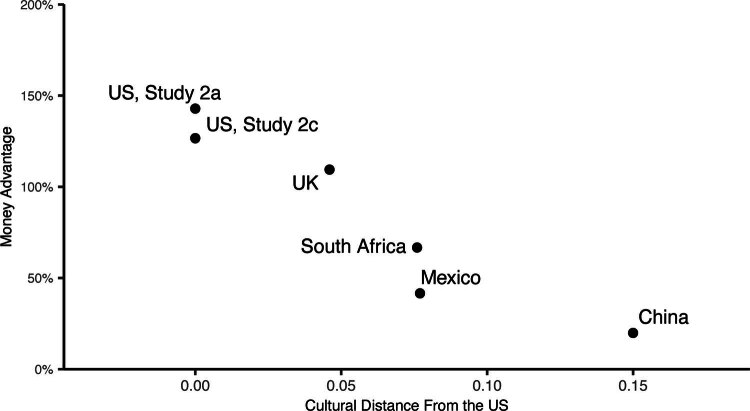
Studies 2a–c, Mapping the Money Advantage on Cultural Distance from the US. Mapping the difference in the effectiveness of monetary and non-monetary treatments (money advantage) from the samples with the same nominal pay in Studies 2a–c on the cultural distance from the US. Cultural distance scores are taken from a previous study^140^.

Furthermore, although we use the terms ‘WEIRD’ and ‘non-WEIRD’ throughout the paper, we agree with the researchers who coined this term that the underlying cultural differences are not binary^18,139–141^. One way to move from the WEIRD–non-WEIRD dichotomy into a more nuanced continuum is the calculation of cultural distance. Researchers used responses to the World Values Survey to calculate different cultures’ ‘distance’ from the United States^140^. Interestingly, these cultural distance scores follow the strength of the money advantage in our studies (Extended Data Fig. 5). Future studies with more cultures and identical conditions can more robustly test the predictive power of cultural distance.

The findings in this paper are a reminder that we should avoid extrapolating the conclusions from studies based on the 12% of the world population that lives in WEIRD cultures to the remaining 88% (ref. ^18^). Instead, monetary—relative to psychological—incentives may increase effort less in non-WEIRD cultures. Across the cultures we have studied, the money advantage was largest in the United States and smallest in China. Accordingly, when designing interventions and reward schemes, particularly under limited resources, the relative benefit of adding a pay-per-effort incentive might be attenuated in non-WEIRD cultures.

## Methods

All studies were carried out in accordance with all the ethical regulations and were approved by the Institutional Review Board (IRB15-1623 and IRB20-1056) at the University of Chicago. Informed consent was obtained from study participants consistent with the IRB protocol.

### Study 1

#### Categorizing treatments

The original ref. ^8^ experiment included a total of 18 incentive treatments, to which participants from both India and the United States were randomly assigned. Out of those 18 treatments, we first selected all pay-for-effort treatments with linear, immediate and guaranteed piece-rates (for example, “You will be paid an extra 1 cent for every 100 points”) and categorized them as ‘monetary.’ These monetary treatments included a range of pay-for-effort incentives with varying linear piece-rates, ranging from 1 cent per 1,000 button presses to 10 cents per 100 button presses.

Using the criteria of linear, immediate and guaranteed piece-rates, we excluded those conditions that, for instance, tested for loss aversion (“You will be paid an extra 40 cents if you score at least 2,000 points”) or delay discounting (“You will be paid an extra 1 cent for every 100 points” with a 4-week delay). The reasons for these exclusions were that the former conditions only offered additional (lump) payment upon reaching a high number of presses, while the latter offered extra payment that was not immediate.

To create a list of non-monetary conditions, we selected treatments where the participants could not earn any additional money for themselves, neither immediately nor at some point in the future, and categorized them as ‘non-monetary’. The non-monetary treatments included conditions with incentives labelled by ref. ^8^ as ‘social psychological’ (such as the social-norm treatment “Many participants scored more than 2,000”), the two charity conditions, where the participants could earn money for the Red Cross but not for themselves, as well as the flat-fee ‘control’ condition (“Your score will not affect your payment”).

Supplementary Table 9a summarizes all the treatments we included on the basis of these criteria and Supplementary Table 9b lists all the treatments we excluded, together with the corresponding reasons for exclusion. Our final sample consisted of 6,294 participants: 5,526 in the United States and 768 in India. Demographic information is available in Supplementary Table 11a.

### Study 2a–c

#### Establishing the minimal amount of effort

Instead of the button-pressing task, we asked participants to classify whether images contained a building or not. We kept the original 10-min limit but made it explicit to participants (including a comprehension check) that they were free to quit the task after every 10 image ratings without losing their base pay. We did this to remove any ambiguity regarding whether participants would be punished for not exerting a lot of effort, particularly in the non-monetary conditions, and because we were worried that non-WEIRD participants might be more inclined to think they might be penalized for doing so.

#### Internet connectivity

We also assumed that people might be faster at rating images on their computers than on their phones or tablets and that people in different countries might have different Internet speeds. While the main effect of culture on effort was not our primary variable of interest, we still chose to limit the participants to laptop or desktop computers only. We also asked participants to self-report how long it took for images to load. This allowed us to rule out technological differences across countries as an explanation for the pattern of findings. Analyses controlling for Internet connectivity are presented in Supplementary Table 13a.

#### Work meaning

Because the meaning behind work matters for the effectiveness of incentives^106,142^, we wanted to replicate the findings from Study 1, in which the work was explicitly meaningless, on a task where participants would be provided with some purpose behind the monotonous work. Therefore, we told participants that image classifications would help the researchers with ‘training a machine-learning algorithm’. The main rationale behind this change was the assumption that most real-life work tasks are not as devoid of meaning as pressing two buttons interchangeably for no apparent reason.

#### Base pay and piece-rates

Our goal was to select a piece-rate that would provide a sufficiently strong incentive for the participants to exert effort. However, we did not want the piece-rate to be so high as to remove any meaningful variation. We agreed to set the base pay across studies to $1.30 and the piece-rate in the monetary condition to 5 cents per 10 images (unless otherwise noted). Participants did not receive partial pay for rating fewer than 10 images within each increment. That is, a participant who rated 11 images and a participant who rated 19 images would both receive a bonus of 5 cents.

On the basis of a pilot, we expected the participants in the monetary condition to earn, on average, an additional 30 to 50 cents, a nominal amount and proportion of final earnings (relative to base pay for taking the study) between those of the ‘1 cent per 100 presses’ and ‘4 cents per 100 presses’ conditions in ref. ^8^. However, we estimated that participants working their hardest would be able to complete at least 150 images within the span of 10 min and thus earn 75 cents.

#### Incentive treatments

We wanted to have the minimal number of conditions to meaningfully test our hypothesis with a sufficiently large sample. Across Studies 2a–c, we kept one pay-for-effort incentive condition and two non-monetary conditions.

In Studies 2a and b, the non-monetary conditions included a social norm condition, where the participants were told that many other participants tried hard on the task and assessed 160 images, and a flat-fee condition, where no additional instructions were given. In Study 2c, we changed the non-monetary treatments. In one of them, participants read that the task was a competition and that they would see how they did relative to others upon completion (competition condition). In the other, participants read that they would not receive any additional pay; however, the researchers would transfer 5 cents for every 10 images participants rated to the Red Cross (charity condition).

In all the non-monetary conditions, the amount of money one earned was not contingent on how much effort one exerted. Every participant received the same salary regardless of how many images they rated. Yet, receiving a fixed salary might invoke norms with regards to obligations and reciprocity, which can differ across cultures^143,144^. Since the pay-off structure was the same across all the non-monetary conditions, we first pooled across the non-monetary conditions in each study and compared them to the monetary condition.

#### Procedure

Participants first read the consent form. Then they read about the nature of the task and were told that they would receive their base pay if they completed at least 10 image ratings. Next, participants had to pass an attention check that asked them about the purpose of the task mentioned on the previous page (to train a machine-learning image-classification database). After that, they learned about the structure of the task and their condition assignment. They had to pass two comprehension checks: one asking them about the maximum duration of the task (10 min) and the other asking them whether they would receive additional pay on the basis of how many images they rated. Further detail and exact wordings are provided in ‘Attention and comprehension checks’ in Supplementary Information.

Only those participants who passed the attention check and both comprehension checks proceeded to the image-rating task that contained the dependent variable. The participants saw several images one by one, with a 10-min timer visible. Images included pictures of plants, flowers, urban landscapes and buildings (for examples of images used in the picture-rating task, see Supplementary Images 1 and 2). For each image, the participants had to answer whether it contained a building.

After rating the first 10 images, the participants saw a screen that asked them whether they wanted to quit or continue with the task. If they chose to quit, they proceeded with the other questions in the survey. Participants who continued with the image-rating task saw a similar screen after rating every 10 images.

#### Cost-effectiveness

To calculate the amount of effort per dollar spent, we divided the number of images each participant rated by the pay they received. Confidence intervals for the amount per dollar spent are 95% confidence intervals of the mean effort divided by the cost in dollar amount adjusted for each level of effort. This cost would be the same for the non-monetary conditions for all amounts of effort within the confidence interval (except for the charity condition, which entailed an additional cost to implement contingent on participants’ effort). However, it would vary in the monetary conditions (that is, would be higher for higher values within the confidence interval).

### Study 2a

#### Participants

In China, we recruited participants (*N* = 1,086) who were either students at Hubei University (*N* = 188) or users of two major social media in China: WeChat Moments (*N* = 79) or QQ Zones (*N* = 819). In the United Kingdom, we recruited participants on Prolific (*N* = 1,067). A colleague from Hubei University completed the translation. Demographic information is available in Supplementary Table 11b.

#### Incentive treatments

Participants were randomly assigned to one of the three conditions: monetary, flat fee and norm. The base pay was ¥8.25 ($1.30 at the time of the experiment) in China and $1.30 in the United Kingdom. In the monetary condition, the bonus for every additional 10 images in the monetary condition was ¥0.30 ($0.05) in China and $0.05 in the United Kingdom.

### Study 2b

#### Participants

Participants for all three samples were recruited from Prolific: *N* = 1,053 in Mexico; *N* = 1,098 in the US sample with the same nominal pay; *N* = 1,122 in the US sample with increased pay pre-tested to be subjectively equivalent^107^ to that in Mexico. The participants in Mexico completed the study in Spanish, while those in the United States did so in English. A professional translator and a Spanish-native research assistant completed the translation. Demographic information is available in Supplementary Table 11b.

#### Incentive treatments

Participants were randomly assigned to one of the three conditions: monetary, flat fee and social norm. The incentive treatments were identical to those in Study 2a, except for the increase in pay in one of the US samples, as described below. Participants in Mexico received a $1.30 base pay for completing the study and, in the monetary condition, a $0.05 bonus per 10 image ratings. The two US samples (described in the next section) differed in both the flat fee that the participants received ($1.30 vs $1.56) and in the piece-rate ($0.05 vs $0.06 per 10 images) in the monetary condition.

#### Becker–DeGroot–Marshak procedure

To address the limitation of Study 1 and 2a, namely, the potentially different subjective values of the pay amounts between the samples: for Study 2b, we collected three samples of participants on Prolific: one from Mexico (*N* = 1,053) and two from the United States, with one US sample having the same nominal pay as in Mexico (*N* = 1,098) and the other having the same subjective pay as in Mexico (*N* = 1,122). Participants in the United States completed the study in English, while those in Mexico did so in Spanish.

To determine subjectively equivalent pay amounts for Prolific participants from Mexico and from the United States, we followed the Becker–DeGroot–Marschak (BDM) procedure^107^. A separate group of participants got briefly acquainted with the task and were then asked how much remuneration they would need to complete its full version. A detailed description of the procedure and the results is provided in ‘Becker–Degroot–Marshak (BDM) procedure for establishing pay equivalence’ in Supplementary Information.

### Study 2c

#### Pre-registration

The study was pre-registered on 3 February 2023. The pre-registration is available on AsPredicted: https://aspredicted.org/dm562.pdf. The study did not deviate from the pre-registration. We pre-registered a total of 665 participants per sample to have enough power to detect an effect size of *f* = 0.14 for the interaction between incentive type and culture.

#### Participants

As in Study 2b, we recruited three samples of participants on Prolific: one from South Africa (*N* = 649) and two from the United States, with one US sample having the same nominal pay as in South Africa (*N* = 662) and the other having the same subjective pay as in South Africa (*N* = 662). All participants completed the survey in English. We pre-screened participants in South Africa who were fluent in English. Demographic information is available in Supplementary Table 11b.

#### Incentive treatments

Unlike in Studies 2a and b, participants in this study were first randomly assigned to types of incentives (monetary or non-monetary) and not individual incentive treatments. Those assigned to the non-monetary incentive type were then randomly assigned to either the competition or the charity condition. Thus, incentive types have roughly identical numbers of people per cell (∼330), but each individual non-monetary treatment had half as many people (∼165) as the monetary condition. In our previous studies, the number of participants was the same in each individual condition, not in each type of incentive condition (monetary or non-monetary). The pay structure in the monetary condition is described in the next section.

#### BDM procedure

To determine subjectively equivalent pay amounts between Prolific participants from South Africa and from the United States, we similarly followed the BDM procedure^107^. A separate group of participants got briefly acquainted with the task and were then asked how much remuneration they would need to complete the full version of the survey.

The two US samples differed in both the flat fee that participants received ($1.30 vs $2.25) for participation and in the pay-for-effort rate ($0.05 vs $0.09 per 10 images) in the monetary incentive condition. Participants in South Africa received a $1.30 base pay for completing the study in all three conditions and a $0.05 bonus per 10 image ratings in the monetary incentive condition. A detailed description of the procedure and the results are available in ‘Becker–Degroot–Marshak (BDM) procedure for establishing pay equivalence’ in Supplementary Information.

### Study 3a

#### Pre-registration

The study was pre-registered on 25 August 2022, although we deviated from the pre-registration as described below. The pre-registration is available on AsPredicted: https://aspredicted.org/uz5gh.pdf.

The main deviation concerned the analysis plan. We pre-registered data analysis from both countries separately using *t*-tests, with a prediction that, for people in the United States, a small monetary incentive would result in higher effort than emphasizing the social norm; our second prediction was that the two incentives would be statistically indistinguishable from each other in India. While these hypotheses were supported by the data, we believe that we did not have sufficient power to detect the smallest meaningful effect size in India, hence the pre-registered analysis was inappropriate. Instead, we ran a multiple linear regression model, consistent with other studies in the paper, to probe for the interaction between incentive and country. We present the results of the pre-registered analyses in ‘Additional analyses for Study 3a’ in Supplementary Information.

Second, we recruited participants in the United States on Prolific instead of MTurk. The pre-registration stated that recruitment would take place on MTurk. We made this change because we noticed that some US MTurkers had posted reviews of the previous reiterations of the study, informing other MTurkers that it was sufficient to complete only 10 images to receive the pay, which we thought might compromise the quality of the data.

Third, we pre-registered 352 participants per country after exclusions (that is, people who did not rate a single image or those who did not complete the full study and receive payment) to have 80% power to observe a small to medium-sized effect (*d* = 0.30). We sampled more people to allow for exclusions and repeated submissions (exclusion details for all studies are available in ‘Exclusion data and criteria for Studies 2–4’ in Supplementary Information). Fewer people than we had estimated did not meet the inclusion criteria in the United States, hence the final sample included 382 people in the United States and 352 people in India. Excluding the last 30 participants in the United States did not significantly change any of the main results, and these analyses are reported in ‘Additional analyses for Study 3a’ in Supplementary Information.

#### Participants

In India (*N* = 352), we recruited people on MTurk. In the United States (*N* = 382), we recruited people on Prolific. Participants in both countries completed the survey in English. Demographic information is available in Supplementary Table 11c.

#### Incentive treatments

Participants were randomly assigned to one of the three conditions: minimal pay or norm. Procedure was identical to the one used in Studies 2a–c, except for the base pay that was $1.00 (as opposed to $1.30 in previous studies) in both India and the United States across conditions. The norm condition was identical to the one in Studies 2a and b. In the minimal-pay condition, the participants could earn an extra cent for completing every 20 image ratings.

### Study 4

#### Participants

We recruited participants through the Center for Social and Behavior Change at Ashoka University, a private university in Haryana, India. The final sample consisted of 2,065 participants recruited through advertisements on the Facebook group ‘QMaths’. This group has over 280,000 members interested in preparing for competitive exams for jobs in sectors ranging from banking to railways. We selected this Facebook group because (1) Ashoka had an ongoing relationship with one of the moderators of the group and (2) the members would generally be proficient in English and Hindi. Demographic information is available in Supplementary Table 11d.

#### Language

Participants were randomly assigned to complete the survey in Hindi or in English. Participants in the Hindi condition completed the whole survey, starting with the consent form, in Hindi. Participants in the English condition completed the entire survey in English. Two research assistants from Ashoka University completed the translation from English into Hindi, occasionally changing the original English wordings to ensure compatibility between the two languages.

We included checks to ensure that participants were proficient in both languages. One week before taking the survey for Study 4, participants completed another survey for which they had to report being ‘very good’ or ‘fluent’ speakers of both English and Hindi.

#### Incentive treatments

Participants were randomly assigned to incentive and language conditions. To ensure a sufficiently high number of participants per condition, we kept two conditions: one monetary condition and one non-monetary condition (social norm). Therefore, the study followed a 2 language (English, Hindi) × 2 incentive (monetary, non-monetary) between-subjects design. The social norm condition was the same as in the previous studies. In the monetary condition, participants received a monetary bonus of ₹5 rupees ($0.0665) for every 10 images they rated. Everyone received ₹150 ($1.995) for completing the main experimental task. To calculate cost-efficiency, we used the exchange rate on the day the last response was collected (7 December 2021; ₹1 = $0.0133).

#### Additional variables

We included seven exploratory variables to measure participants’ perceptions of the task and motivations for completing it. These variables were all measured on a 7-point Likert scale (1 = strongly disagree to 7 = strongly agree). Participants reported their agreement with the following statements: “I enjoyed completing the task’; “I am satisfied with how well I did on the task”; “I believe that I helped others by completing the task”; “Completing the task was boring”; “I only completed the task for money”; “I could have assessed more pictures if I'd tried harder”; *“*I am satisfied with the amount of pay I received”. Details are available in ‘Exploratory variables’ in Supplementary Information.

### Reporting summary

Further information on research design is available in the Nature Portfolio Reporting Summary linked to this article.

### Supplementary information

Supplementary Information Table of contents provided on the first two pages.

Reporting Summary

## Data Availability

All de-identified raw data are available at https://osf.io/8yu95/.

## Code availability

All code is available at https://osf.io/8yu95/. All analyses were performed in RStudio with the following packages: Hmisc, tidyverse, rstatix, apaTables, ggrepel, knitr, ggpubr and readxl.

## References

[1] Weber, M. *The Protestant Ethic and the Spirit of Capitalism* (Routledge, 1905).

[2] Touré-Tillery M, Fishbach A (2018). Three sources of motivation. Consum. Psychol. Rev..

[3] Hutson D (2000). New incentives are on the rise. Compens. Benefits Rev..

[4] Ashraf N, Bandiera O, Jack BK (2014). No margin, no mission? A field experiment on incentives for public service delivery. J. Public Econ..

[5] Cassar L, Meier S (2018). Nonmonetary incentives and the implications of work as a source of meaning. J. Econ. Perspect..

[6] Erkal N, Gangadharan L, Koh BH (2018). Monetary and non-monetary incentives in real-effort tournaments. Eur. Econ. Rev..

[7] Jenkins GD, Mitra A, Gupta N, Shaw JD (1998). Are financial incentives related to performance? A meta-analytic review of empirical research. J. Appl. Psychol..

[8] DellaVigna S, Pope D (2018). What motivates effort? Evidence and expert forecasts. Rev. Econ. Stud..

[9] Campos-Mercade P (2021). Monetary incentives increase COVID-19 vaccinations. Science.

[10] Milkman KL (2021). Megastudies improve the impact of applied behavioural science. Nature.

[11] Thomas CC, Markus HR (2023). Enculturating the science of international development: beyond the WEIRD independent paradigm. J. Cross Cult. Psychol..

[12] Estrada-Villalta S, Adams G (2018). Decolonizing development: a decolonial approach to the psychology of economic inequality. Transl. Issues Psychol. Sci..

[13] Kizilcec RF, Cohen GL (2017). Eight-minute self-regulation intervention raises educational attainment at scale in individualist but not collectivist cultures. Proc. Natl Acad. Sci. USA.

[14] Sloan TS (1990). Psychology for the third world?. J. Soc. Issues.

[15] Brady LM, Fryberg SA, Shoda Y (2018). Expanding the interpretive power of psychological science by attending to culture. Proc. Natl Acad. Sci. USA.

[16] Sinha JBP (1984). Towards partnership for relevant research in the third world. Int. J. Psychol..

[17] Domenech Rodríguez MM (2018). Scaling out evidence-based interventions outside the US mainland: social justice or Trojan horse?. J. Lat. Psychol..

[18] Henrich J, Heine SJ, Norenzayan A (2010). The weirdest people in the world?. Behav. Brain Sci..

[19] Harguess JM, Crespo NC, Hong MY (2020). Strategies to reduce meat consumption: a systematic literature review of experimental studies. Appetite.

[20] Burnette JL (2022). A systematic review and meta-analysis of growth mindset interventions: for whom, how, and why might such interventions work?. Psychol. Bull..

[21] Liebe U, Gewinner J, Diekmann A (2018). What is missing in research on non-monetary incentives in the household energy sector?. Energy Policy.

[22] *World Development Indicators* (World Bank, 2021).

[23] Nielsen M, Haun D, Kärtner J, Legare CH (2017). The persistent sampling bias in developmental psychology: a call to action. J. Exp. Child Psychol..

[24] Rad MS, Martingano AJ, Ginges J (2018). Toward a psychology of *Homo sapiens*: making psychological science more representative of the human population. Proc. Natl Acad. Sci. USA.

[25] Cheon BK, Melani I, Hong Y (2020). How USA-centric is psychology? An archival study of implicit assumptions of generalizability of findings to human nature based on origins of study samples. Soc. Psychol. Personal. Sci..

[26] Bossuroy T (2022). Tackling psychosocial and capital constraints to alleviate poverty. Nature.

[27] Dalton PS, Rüschenpöhler J, Uras B, Zia B (2021). Curating local knowledge: experimental evidence from small retailers in Indonesia. J. Eur. Econ. Assoc..

[28] Wu SJ, Paluck EL (2021). Designing nudges for the context: golden coin decals nudge workplace behavior in China. Organ. Behav. Hum. Decis. Process..

[29] Bandiera O (2017). Labor markets and poverty in village economies. Q. J. Econ..

[30] Bursztyn L, Fiorin S, Gottlieb D, Kanz M (2019). Moral incentives in credit card debt repayment: evidence from a field experiment. J. Polit. Econ..

[31] Kapoor, H. et al. Does incentivization promote sharing ‘true’ content online? *Harv. Kennedy Sch. Misinformation Rev.*10.37016/mr-2020-120 (2023).

[32] Polanyi, K. in *Trade market in the Early Empires* (eds Polanyi, K. et al.) 243–270 (The Free Press, 1957).

[33] Thompson EP (1971). The moral economy of the English crowd in the eighteenth century. Past Present.

[34] Scott, J. C. *The Moral Economy of the Peasant: Rebellion and Subsistence in Southeast Asia* (Yale Univ. Press, 1976).

[35] Mauss, M. *The Gift: The Form and Reason for Exchange in Archaic Societies* (WW Norton & Company, 2000).

[36] Greif A (1993). Contract enforceability and economic institutions in early trade: the Maghribi traders’ coalition. Am. Econ. Rev..

[37] Fernández R, Fogli A (2009). Culture: an empirical investigation of beliefs, work, and fertility. Am. Econ. J. Macroecon..

[38] Boyer P, Petersen MB (2018). Folk-economic beliefs: an evolutionary cognitive model. Behav. Brain Sci..

[39] Guiso L, Sapienza P, Zingales L (2006). Does culture affect economic outcomes?. J. Econ. Perspect..

[40] Henrich J (2005). ‘Economic man’ in cross-cultural perspective: behavioral experiments in 15 small-scale societies. Behav. Brain Sci..

[41] Markus HR (2016). What moves people to action? Culture and motivation. Curr. Opin. Psychol..

[42] Roth AE, Prasnikar V, Okuno-Fujiwara M, Zamir S (1991). Bargaining and market behavior in Jerusalem, Ljubljana, Pittsburgh, and Tokyo: an experimental study. Am. Econ. Rev..

[43] Shao L, Kwok CC, Guedhami O (2010). National culture and dividend policy. J. Int. Bus. Stud..

[44] Fisman R, Miguel E (2007). Corruption, norms, and legal enforcement: evidence from diplomatic parking tickets. J. Polit. Econ..

[45] Chen MK (2013). The effect of language on economic behavior: evidence from savings rates, health behaviors, and retirement assets. Am. Econ. Rev..

[46] Alesina A, Giuliano P (2015). Culture and institutions. J. Econ. Lit..

[47] Biernacki, R. *The Fabrication of Labor: Germany and Britain, 1640–1914* Vol. 22 (Univ. of California Press, 1995).

[48] Adler NJ, Jelinek M (1986). Is ‘organization culture’ culture bound?. Hum. Resour. Manage..

[49] Blau, P. M. & Scott, W. R. *Formal Organizations: A Comparative Approach* (Chandler, 1962).

[50] Baron, J. N. & Kreps, D. M. in *Handbook of Organizational Economics* (eds Gibbons, R. & Roberts, J.) 315–341 (Princeton Univ. Press, 2012).

[51] Westwood R, Chan A, Linstead S (2004). Theorizing Chinese employment relations comparatively: exchange, reciprocity and the moral economy. Asia Pac. J. Manage..

[52] Fiske, A. P. *Structures of Social Life: The Four Elementary Forms of Human Relations: Communal Sharing, Authority Ranking, Equality Matching, Market Pricing* (Free Press, 1991).

[53] Li Y, Zahra SA (2012). Formal institutions, culture, and venture capital activity: a cross-country analysis. J. Bus. Ventur..

[54] Tiessen JH (1997). Individualism, collectivism, and entrepreneurship: a framework for international comparative research. J. Bus. Ventur..

[55] Raja U, Johns G, Ntalianis F (2004). The impact of personality on psychological contracts. Acad. Manage. J..

[56] Thomas DC, Au K, Ravlin EC (2003). Cultural variation and the psychological contract. J. Organ. Behav..

[57] Bowles S (1998). Endogenous preferences: the cultural consequences of markets and other economic institutions. J. Econ. Lit..

[58] Rousseau DM, Parks JM (1993). The contracts of individuals and organizations. Res. Organ. Behav..

[59] Boyacigiller NA, Adler NJ (1991). The parochial dinosaur: organizational science in a global context. Acad. Manage. Rev..

[60] Macchiavello R (2022). Relational contracts and development. Annu. Rev. Econ..

[61] McMillan J, Woodruff C (1999). Interfirm relationships and informal credit in Vietnam. Q. J. Econ..

[62] Board S (2011). Relational contracts and the value of loyalty. Am. Econ. Rev..

[63] Johnson S, McMillan J, Woodruff C (2002). Courts and relational contracts. J. Law Econ. Organ..

[64] Camuffo A, Furlan A, Rettore E (2007). Risk sharing in supplier relations: an agency model for the Italian air‐conditioning industry. Strateg. Manage. J..

[65] Singh U, Srivastava KB (2009). Interpersonal trust and organizational citizenship behavior. Psychol. Stud..

[66] Miller JG, Akiyama H, Kapadia S (2017). Cultural variation in communal versus exchange norms: implications for social support. J. Personal. Soc. Psychol..

[67] Chen H, Bolton LE, Ng S, Lee D, Wang D (2018). Culture, relationship norms, and dual entitlement. J. Consum. Res..

[68] Greif A (1994). Cultural beliefs and the organization of society: a historical and theoretical reflection on collectivist and individualist societies. J. Polit. Econ..

[69] White K, Lehman DR (2005). Culture and social comparison seeking: the role of self-motives. Personal. Soc. Psychol. Bull..

[70] Oyserman D, Coon HM, Kemmelmeier M (2002). Rethinking individualism and collectivism: evaluation of theoretical assumptions and meta-analyses. Psychol. Bull..

[71] Dore R (1983). Goodwill and the spirit of market capitalism. Br. J. Sociol..

[72] DiMaggio P (1997). Culture and cognition. Annu. Rev. Sociol..

[73] Meeker BF (1971). Decisions and exchange. Am. Sociol. Rev..

[74] Hui C, Lee C, Rousseau DM (2004). Psychological contract and organizational citizenship behavior in China: investigating generalizability and instrumentality. J. Appl. Psychol..

[75] Mandel DR (2006). Economic transactions among friends: asymmetric generosity but not agreement in buyers’ and sellers’ offers. J. Conflict Resolut..

[76] Bandiera O, Barankay I, Rasul I (2010). Social incentives in the workplace. Rev. Econ. Stud..

[77] Rodríguez CM, Wilson DT (2002). Relationship bonding and trust as a foundation for commitment in US–Mexican strategic alliances: a structural equation modeling approach. J. Int. Mark..

[78] Barron K, Chou SY (2016). Developing normative commitment as a consequence of receiving help – the moderated mediating roles of team-member exchange and individualism/collectivism: a multi-level model. J. Manage. Sci..

[79] Johnson NB, Droege S (2004). Reflections on the generalization of agency theory: cross-cultural considerations. Hum. Resour. Manage. Rev..

[80] Gardner DG, Huang G-H, Niu X, Pierce JL, Lee C (2015). Organization‐based self‐esteem, psychological contract fulfillment, and perceived employment opportunities: a test of self‐regulatory theory. Hum. Resour. Manage..

[81] Akerlof GA (1982). Labor contracts as partial gift exchange. Q. J. Econ..

[82] Baron JN (2013). Empathy wages?: gratitude and gift exchange in employment relationships. Res. Organ. Behav..

[83] Ghani T, Reed T (2022). Relationships on the rocks: contract evolution in a market for ice. Am. Econ. J. Microecon..

[84] Ipeirotis, P. G. *Demographics of Mechanical Turk*. NYU Working Paper No. CEDER-10-01 (2010).

[85] King RC, Bu N (2005). Perceptions of the mutual obligations between employees and employers: a comparative study of new generation IT professionals in China and the United States. Int. J. Hum. Resour. Manage..

[86] Corney WJ, Richards CH (2005). A comparative analysis of the desirability of work characteristics: Chile versus the United States. Int. J. Manage..

[87] Chiang FF, Birtch TA (2012). The performance implications of financial and non‐financial rewards: an Asian Nordic comparison. J. Manage. Stud..

[88] House, R. J., Hanges, P. J., Javidan, M., Dorfman, P. W. & Gupta, V. *Culture, Leadership, and Organizations: The GLOBE Study of 62 Societies* (Sage Publications, 2004).

[89] Oyserman, D. in *International Encyclopedia of the Social and Behavioral Sciences* Vol. 25 (ed. Wright, J. D.) 36–40 (Elsevier, 2015).

[90] Miles A (2015). The (re) genesis of values: examining the importance of values for action. Am. Sociol. Rev..

[91] Shteynberg G, Gelfand MJ, Kim K (2009). Peering into the ‘magnum mysterium’ of culture: the explanatory power of descriptive norms. J. Cross Cult. Psychol..

[92] Morris MW, Hong Y, Chiu C, Liu Z (2015). Normology: integrating insights about social norms to understand cultural dynamics. Organ. Behav. Hum. Decis. Process..

[93] Heath C (1999). On the social psychology of agency relationships: lay theories of motivation overemphasize extrinsic incentives. Organ. Behav. Hum. Decis. Process..

[94] Vuolevi JH, Van Lange PA (2010). Beyond the information given: the power of a belief in self‐interest. Eur. J. Soc. Psychol..

[95] Miller DT (1999). The norm of self-interest. Am. Psychol..

[96] Chatman JA, Barsade SG (1995). Personality, organizational culture, and cooperation: evidence from a business simulation. Adm. Sci. Q..

[97] Kasser T, Cohn S, Kanner AD, Ryan RM (2007). Some costs of American corporate capitalism: a psychological exploration of value and goal conflicts. Psychol. Inq..

[98] Kitayama S, Park J (2014). Error-related brain activity reveals self-centric motivation: culture matters. J. Exp. Psychol. Gen..

[99] DeVoe SE, Iyengar SS (2004). Managers’ theories of subordinates: a cross-cultural examination of manager perceptions of motivation and appraisal of performance. Organ. Behav. Hum. Decis. Process..

[100] Schuler RS, Rogovsky N (1998). Understanding compensation practice variations across firms: the impact of national culture. J. Int. Bus. Stud..

[101] Bandiera, O., Dahlstrand-Rudin, A. & Fischer, G. Incentives and culture: evidence from a multi-country field experiment. *AEA RCT* Registry 10.1257/rct.4685-2.1 (2020).

[102] Gooderham P, Fenton-O’Creevy M, Croucher R, Brookes M (2018). A multilevel analysis of the use of individual pay-for-performance systems. J. Manage..

[103] Gorodnichenko Y, Roland G (2011). Individualism, innovation, and long-run growth. Proc. Natl Acad. Sci. USA.

[104] Gneezy U (2019). Measuring success in education: the role of effort on the test itself. Am. Econ. Rev. Insights.

[105] Fryer RG (2011). Financial incentives and student achievement: evidence from randomized trials. Q. J. Econ..

[106] Ariely D, Kamenica E, Prelec D (2008). Man’s search for meaning: the case of Legos. J. Econ. Behav. Organ..

[107] Becker GM, DeGroot MH, Marschak J (1964). Measuring utility by a single‐response sequential method. Behav. Sci..

[108] Suh E, Diener E, Oishi S, Triandis HC (1998). The shifting basis of life satisfaction judgments across cultures: emotions versus norms. J. Personal. Soc. Psychol..

[109] Adams BG, Van de Vijver FJ, De Bruin GP (2012). Identity in South Africa: examining self-descriptions across ethnic groups. Int. J. Intercult. Relat..

[110] Eaton L, Louw J (2000). Culture and self in South Africa: individualism-collectivism predictions. J. Soc. Psychol..

[111] Triandis HC, Bontempo R, Villareal MJ, Asai M, Lucca N (1988). Individualism and collectivism: cross-cultural perspectives on self-ingroup relationships. J. Personal. Soc. Psychol..

[112] Triandis HC, Gelfand MJ (1998). Converging measurement of horizontal and vertical individualism and collectivism. J. Personal. Soc. Psychol..

[113] Einolf CJ (2017). Cross-national differences in charitable giving in the west and the world. Voluntas.

[114] Wiepking P (2021). The global study of philanthropic behavior. Voluntas.

[115] Rani, U. & Furrer, M. Digital labour platforms and new forms of flexible work in developing countries: Algorithmic management of work and workers. *Compet. Change* **25**, 212–236 (2021).

[116] Berg, J., Furrer, M., Harmon, E., Rani, U. & Silberman, M. S. *Digital Labour Platforms and the Future of Work: Towards Decent Work in the Online World*. (International Labour Organization, 2018).

[117] Morschheuser B, Hamari J (2019). The gamification of work: lessons from crowdsourcing. J. Manage. Inq..

[118] Koivisto J, Hamari J (2019). The rise of motivational information systems: a review of gamification research. Int. J. Inf. Manage..

[119] Bouchrika I, Harrati N, Wanick V, Wills G (2021). Exploring the impact of gamification on student engagement and involvement with e-learning systems. Interact. Learn. Environ..

[120] Benet-Martínez V, Leu J, Lee F, Morris MW (2002). Negotiating biculturalism: cultural frame switching in biculturals with oppositional versus compatible cultural identities. J. Cross Cult. Psychol..

[121] Luna D, Ringberg T, Peracchio LA (2008). One individual, two identities: frame switching among biculturals. J. Consum. Res..

[122] Ralston DA, Cunniff MK, Gustafson DJ (1995). Cultural accommodation: the effect of language on the responses of bilingual Hong Kong Chinese managers. J. Cross Cult. Psychol..

[123] Trafimow D, Silverman ES, Fan RM-T, Fun Law JS (1997). The effects of language and priming on the relative accessibility of the private self and the collective self. J. Cross Cult. Psychol..

[124] Verkuyten M, Pouliasi K (2002). Biculturalism among older children: cultural frame switching, attributions, self-identification, and attitudes. J. Cross Cult. Psychol..

[125] Hong Y, Morris MW, Chiu C, Benet-Martinez V (2000). Multicultural minds: a dynamic constructivist approach to culture and cognition. Am. Psychol..

[126] Altbach PG (2009). The giants awake: higher education systems in China and India. Econ. Polit. Wkly.

[127] Si A (2011). A diachronic investigation of Hindi–English code-switching, using Bollywood film scripts. Int. J. Biling..

[128] Thaler RH (2000). From Homo economicus to Homo sapiens. J. Econ. Perspect..

[129] Ferraro F, Pfeffer J, Sutton RI (2005). Economics language and assumptions: how theories can become self-fulfilling. Acad. Manage. Rev..

[130] Binmore K (2005). Economic man – or straw man?. Behav. Brain Sci..

[131] Loewenstein GF, Thompson L, Bazerman MH (1989). Social utility and decision making in interpersonal contexts. J. Personal. Soc. Psychol..

[132] Daniels AK (1987). Invisible work. Soc. Probl..

[133] Zaki J, Neumann E, Baltiansky D (2021). Market cognition: how exchange norms alter social experience. Curr. Dir. Psychol. Sci..

[134] Wilmers N, Zhang L (2022). Values and inequality: prosocial jobs and the college wage premium. Am. Sociol. Rev..

[135] Bubonya M, Cobb-Clark DA, Wooden M (2017). Mental health and productivity at work: does what you do matter?. Labour Econ..

[136] Baktash MB, Heywood JS, Jirjahn U (2022). Worker stress and performance pay: German survey evidence. J. Econ. Behav. Organ..

[137] Hur JD, Lee-Yoon A, Whillans AV (2021). Are they useful? The effects of performance incentives on the prioritization of work versus personal ties. Organ. Behav. Hum. Decis. Process..

[138] Gomez-Mejia LR, Welbourne T (1991). Compensation strategies in a global context. Hum. Resour. Plan..

[139] Graham J, Meindl P, Beall E, Johnson KM, Zhang L (2016). Cultural differences in moral judgment and behavior, across and within societies. Curr. Opin. Psychol..

[140] Muthukrishna M (2020). Beyond Western, Educated, Industrial, Rich, and Democratic (WEIRD) psychology: measuring and mapping scales of cultural and psychological distance. Psychol. Sci..

[141] Schulz, J., Bahrami-Rad, D., Beauchamp, J. & Henrich, J. The origins of WEIRD psychology. *SSRN*10.2139/ssrn.3201031 (2018).

[142] Kosfeld M, Neckermann S, Yang X (2017). The effects of financial and recognition incentives across work contexts: the role of meaning. Econ. Inq..

[143] Hitokoto H (2016). Indebtedness in cultural context: the role of culture in the felt obligation to reciprocate. Asian J. Soc. Psychol..

[144] Shen H, Wan F, Wyer RS (2011). Cross-cultural differences in the refusal to accept a small gift: the differential influence of reciprocity norms on Asians and North Americans. J. Personal. Soc. Psychol..

