# Supplementary material for: The motivating effect of monetary over psychological incentives is stronger in WEIRD cultures
Source: Nat Hum Behav. 2024 Jan 8;8(3):456–70. doi: 10.1038/s41562-023-01769-5 (PMC10963269; doi:10.1038/s41562-023-01769-5)
Supplement: Supplementary file 1 — Table of contents provided on the first two pages. [file 41562_2023_1769_MOESM1_ESM.pdf]

nature human behaviour

Article https://doi.org/10.1038/s41562-023-01769-5

The motivating effect of monetary over psychological incentives is stronger in WEIRD cultures

In the format provided by the authors and unedited

SUPPLEMENTARY MATERIALS

Table of Contents

REGRESSION TABLES 1
Supplementary Table 1: Study 1, Pooled Monetary Versus Pooled Non-Monetary Conditions in India and the US. 1
Supplementary Table 2: Study 2a, Monetary Versus Pooled Non-Monetary Conditions in the UK and China. 2
Supplementary Table 3: Study 2b, Monetary Versus Pooled Non-Monetary Conditions in the US and Mexico. 3
Supplementary Table 4: Study 2c, Monetary Versus Pooled Non-Monetary Conditions in the US and South Africa. 4
Supplementary Table 5: Study 3a, Norm Versus Minimal Pay Condition in India and the US. 5
Supplementary Table 6: Study 4, Norm Versus Monetary Condition in India, by Language Prime (English or Hindi). 6
Supplementary Table 7: Study 2a, Monetary Versus Individual Non-Monetary Conditions in the UK and China. 7
Supplementary Table 8: Study 2b, Monetary Versus Individual Non-Monetary Conditions in the US and Mexico. 8

CONDITIONS FROM DELLAVIGNA AND POPE [1] INCLUDED IN AND EXCLUDED FROM STUDY 1 10
Supplementary Table 9a: Monetary Versus Non-Monetary Treatments from DellaVigna and Pope [1] Included in Study 1. 11
Supplementary Table 9b: Treatments from DellaVigna and Pope [1] Excluded from Study 1 and Reasons for Exclusions. 12

ATTENTION AND COMPREHENSION CHECKS 13
Attention Check 13
Comprehension Check 13

IMAGES FROM THE EXPERIMENTAL TASK 14
Supplementary Image 1: Example of an Image from the Image-Rating Task. 14
Supplementary Image 2: Example of an Image from the Image-Rating Task. 15

MAIN EFFECTS FOR REPORTED REGRESSIONS 16
Study 1 16
Study 2A 16
Study 2B 16
Study 2C 17
Study 3A 17
Study 4 17

STUDY 3B: IS MONEY MOTIVATING TO PEOPLE IN THE US BECAUSE OF GAMIFICATION? 18
Methods for Supplementary Study 3b 19

EXCLUSION DATA AND CRITERIA FOR STUDIES 2–4 21
Exclusion Criteria 21
Exclusion Data 21
Note Regarding Samples in Studies 2A and 3A 28
Note Regarding the US Samples in Study 2B 28
Fig. S1: Study 2b, Monetary Versus Pooled Non-Monetary Conditions in Mexico and the US (Duplicate Participants in the US Excluded). 30

MEAN EFFORT AND COST-EFFECTIVENESS IN INDIVIDUAL CONDITIONS IN STUDY 2C31
Fig. S2: Study 2c, Monetary Versus Individual Non-Monetary Conditions in the US and South Africa. 32

Supplementary Table 10: Study 2c, Monetary Versus Individual Non-Monetary Conditions in the US and South Africa. ... 34

PROBABILITY OF QUITTING THE TASK AT THE FIRST OPPORTUNITY IN STUDIES 2A–4 35
Study 2A ... 35
Study 2B ... 35
Study 2C ... 36
Study 3A ... 37
Supplementary Study 3B ... 37
Study 4 ... 38

DEMOGRAPHIC VARIABLES ... 39
Supplementary Table 11a: Demographic Variables for Study 1. ... 39
Supplementary Table 11b: Demographic Variables for Studies 2a–c. ... 40
Supplementary Table 11c: Demographic Variables for Study 3a and Supplementary Study 3b. ... 45
Supplementary Table 11d: Demographic Variables for Study 4. ... 47

BECKER-DEGROOT-MARSHAK (BDM) [2] PROCEDURE FOR ESTABLISHING PAY EQUIVALENCE ... 48
Study 2B ... 48
Study 2C ... 49
Note on Purchasing Power Parity ... 49

ADDITIONAL CONTROLS ... 51
Repeating the Analyses with Interactions Between Country and Each Predictor Variable ... 51
Supplementary Table 12a: Studies 2a–c, Mean Effort in Monetary Versus Pooled Non-Monetary Incentive Conditions, with Interactions Between Country and Predictor Variables. ... 52
Supplementary Table 12b: Study 3a, Mean Effort in Monetary Versus Pooled Non-Monetary Incentive Conditions, with Interactions Between Country and Predictor Variables. ... 55
Repeating the Analyses in Studies 2a–c and 3a with Additional Controls ... 56
Supplementary Table 13a: Studies 2a–c, Mean Effort in Monetary Versus Pooled Non-Monetary Incentive Conditions, with Additional Controls. ... 59
Supplementary Table 13b: Study 3a, Mean Effort in Norm Versus Minimal Pay Condition, with Additional Controls. ... 63

ADDITIONAL ANALYSES FOR STUDY 3A ... 65

EXPLORATORY VARIABLES ... 66
Supplementary Table 14: Exploratory Variables in Study 2a. ... 68
Supplementary Table 15a: Exploratory Variables in Study 2b (Mexico and US Sample with Same Nominal Pay). ... 71
Supplementary Table 15b: Exploratory Variables in Study 2b (Mexico and US Sample with the Same Subjective Pay). ... 74
Supplementary Table 16: Exploratory Variables in Study 4. ... 77
Fig. S3: Exploratory Variables in Study 4, by Language and Incentive Type. ... 79

ADDITIONAL DISCUSSION ... 80
Conceptualizing the Task As “Work” Versus “Non-Work” ... 80
Is The Effect Driven by Money or by Psychological Interventions? ... 81

REFERENCES FOR SUPPLEMENTARY MATERIALS ... 83

REGRESSION TABLES

Supplementary Table 1: **Study 1, Pooled Monetary Versus Pooled Non-Monetary Conditions in India and the US.**

	b	*SE*	*t*	*P*	95% CI
Monetary Incentive	159.85	54.84	2.91	0.004	[52.35, 267.35]
US	195.69	36.04	5.43	< 0.001	[125.05, 266.34]
Female	-143.21	18.82	-7.61	< 0.001	[-180.10, -106.33]
Education	-101.11	18.95	-5.33	< 0.001	[-138.26, -63.95]
Age	-69.92	7.36	-9.50	< 0.001	[-84.35, -55.49]
Monetary Incentive × US	170.56	58.48	2.92	0.004	[55.91, 285.21]

Regression output for the multiple linear regression model on the number of button presses, reported in the Results section of Study 1. Monetary Incentive is a binary variable for whether the participants were in one of the monetary (pay-for-effort) conditions. US is a binary variable for whether the participants were part of the US sample. Female is a categorical variable for gender. Age is measured on a scale, where 1 = “18–24”; 2 = “25–30”; 3 = “31–40”; 4 = “41–50”; 5 = “51–64”; 6 = “65 or higher.” Education is a binary variable specifying people who had either completed or were pursuing an undergraduate degree or higher at the time of the experiment.

Supplementary Table 2: **Study 2a, Monetary Versus Pooled Non-Monetary Conditions in the UK and China.**

	b	*SE*	*t*	*P*	95% CI
Monetary Incentive	8.79	2.83	3.11	0.002	[3.25, 14.33]
UK	2.18	2.76	0.79	0.429	[-3.22, 7.58]
Female	1.13	1.89	0.6	0.551	[-2.58, 4.84]
Non-Binary	10.96	12.66	0.87	0.387	[-13.88, 35.79]
Education	-5.03	2.23	-2.25	0.024	[-9.41, -0.65]
Age	-2.73	1.23	-2.22	0.027	[-5.14, -0.32]
Monetary Incentive × UK	38.40	3.98	9.65	< 0.001	[30.59, 46.20]

Regression output for the multiple linear regression model on the number of images rated in the Study 2a samples from China (N = 1,086 recruited on social media) and the UK (N = 1,067 recruited on Prolific). Monetary Incentive is a binary variable for whether the participants were assigned to the monetary condition. UK is a binary variable for whether the participants were part of the UK sample. Female and Non-Binary are categorical variables for gender. Education is a binary variable specifying people who had either completed or were pursuing an undergraduate degree or higher at the time of the experiment. Age is a continuous variable and is scaled.

Supplementary Table 3: **Study 2b, Monetary Versus Pooled Non-Monetary Conditions in the US and Mexico.**

	b	*SE*	*t*	*P*	95% CI
Mexico and the US (Same Nominal Pay)					
Monetary Incentive	27.49	2.92	9.42	< 0.001	[21.76, 33.21]
US	-37.51	2.62	-14.32	< 0.001	[-42.64, -32.37]
Female	-4.95	1.96	-2.52	0.012	[-8.80, -1.09]
Non-Binary	11.68	6.46	1.81	0.071	[-1.00, 24.36]
Education	-5.91	2.14	-2.76	0.006	[-10.12, -1.71]
Age	-1.36	1.13	-1.21	0.227	[-3.57, 0.85]
Monetary Incentive × US	13.93	4.07	3.42	< 0.001	[5.94, 21.92]
Mexico and the US (Same Subjective Pay)					
Monetary Incentive	27.49	2.95	9.31	< 0.001	[21.70, 33.28]
US	-36.21	2.61	-13.87	< 0.001	[-41.33, -31.09]
Female	-5.57	1.99	-2.80	< 0.001	[-9.47, -1.66]
Non-Binary	11.12	6.49	1.71	0.087	[-1.61, 23.85]
Education	-5.39	2.16	-2.50	0.013	[-9.61, -1.16]
Age	-2.76	1.12	-2.47	0.014	[-4.95, -0.56]
Monetary Incentive × US	19.28	4.09	4.71	< 0.001	[11.26, 27.30]

Regression output for the multiple linear regression on the number of images rated in the Study 2b sample from Mexico (*N* = 1,053 recruited on Prolific) and each of the two Study 2b samples in the US: one with the same nominal pay (*N* = 1,098 on Prolific) as in Mexico and the other with the same subjective [1] pay (*N* = 1,122 recruited on Prolific) as in Mexico. Monetary Incentive is a binary variable for whether the participants were assigned to the monetary condition. US is a binary variable for whether the participants were part of the US sample. Female and Non-Binary are categorical variables for gender. Education is a binary variable specifying people who had either completed or were pursuing an undergraduate degree or higher at the time of the experiment. Age is a continuous variable and is scaled.

Supplementary Table 4: **Study 2c, Monetary Versus Pooled Non-Monetary Conditions in the US and South Africa.**

	b	*SE*	*t*	*P*	95% CI
South Africa and the US (Same Nominal Pay)					
Monetary Incentive	25.29	2.88	8.78	< 0.001	[19.64, 30.93]
US	-8.97	3.02	-2.97	0.003	[-14.90, -3.04]
Female	0.49	2.07	0.24	0.813	[-3.57, 4.55]
Non-Binary	-7.13	7.37	-0.97	0.333	[-21.59, 7.32]
Education	-6.98	2.08	-3.36	0.001	[-11.05, -2.90]
Age	-2.97	1.11	-2.68	0.007	[-5.14, -0.80]
Monetary Incentive × US	7.98	4.05	1.97	0.049	[0.03, 15.92]
South Africa and the US (Same Subjective Pay)					
Monetary Incentive	25.22	2.92	8.63	< 0.001	[19.48, 30.95]
US	-10.78	3.02	-3.57	< 0.001	[-16.70, -4.86]
Female	-2.09	2.09	-1.00	0.318	[-6.19, 2.01]
Non-Binary	-13.06	7.92	-1.65	0.099	[-28.59, 2.47]
Education	-4.21	2.11	-1.99	0.046	[-8.36, -0.07]
Age	-1.02	1.11	-0.92	0.359	[-3.20, 1.16]
Monetary Incentive × US	15.40	4.12	3.74	< 0.001	[7.33, 23.48]

Regression output for the multiple linear regression on the number of images rated in the Study 2c sample from South Africa (N = 649 recruited on Prolific) and each of the two Study 2c samples in the US: one with the same nominal pay (N = 662 recruited on Prolific) as in South Africa and the other with the same subjective [1] pay (N = 662 recruited on Prolific) as in South Africa. Monetary Incentive is a binary variable for whether the participants were assigned to the monetary condition. US is a binary variable for whether the participants were part of the US sample. Female and Non-Binary are categorical variables for gender. Education is a binary variable specifying people who had either completed or were pursuing an undergraduate degree or higher at the time of the experiment. Age is a continuous variable and is scaled.

Supplementary Table 5: **Study 3a, Norm Versus Minimal Pay Condition in India and the US.**

	b	*SE*	*t*	*P*	95% CI
Monetary Incentive	0.83	4.28	0.19	0.847	[-7.57, 9.22]
US	-26.03	4.56	-5.71	< 0.001	[-34.99, -17.08]
Female	2.59	3.23	0.80	0.422	[-3.75, 8.94]
Non-Binary	21.16	11.10	1.91	0.057	[-0.62, 42.95]
Education	-4.45	3.87	-1.15	0.250	[-12.05, 3.14]
Age	-0.42	1.50	-0.28	0.781	[-3.36, 2.52]
Monetary Incentive × US	13.77	5.95	2.31	0.021	[2.09, 25.45]

Regression output for the multiple linear regression on the number of images rated in the Study 3a samples from India (N = 352 recruited on MTurk) and the US (N = 382 recruited on Prolific). Monetary Incentive is a binary variable for whether the participants were assigned to the monetary (minimal pay) condition. US is a binary variable for whether the participants were part of the US sample. Female and Non-Binary are categorical variables for gender. Education is a binary variable specifying people who had either completed or were pursuing an undergraduate degree or higher at the time of the experiment. Age is a continuous variable and is scaled.

***Supplementary Table 6:* Study 4, Norm Versus Monetary Condition in India, by Language Prime (English or Hindi).**

	b	*SE*	*t*	*P*	95% CI
Monetary Incentive	12.63	2.27	5.56	< 0.001	[8.17, 17.09]
English	-1.74	2.28	-0.76	0.445	[-6.22, 2.74]
Monetary Incentive × English	10.69	3.23	3.31	0.001	[4.35, 17.02]

Regression output for the multiple linear regression on the number of images rated in the sample of bilingual participants from India (N = 2,065 recruited on Facebook), reported in the Results section of Study 4. Monetary Incentive is a binary variable for whether participants were assigned to the monetary condition. English is a binary variable for whether participants were assigned to complete the study in English (as compared to in Hindi).

***Supplementary Table 7:* Study 2a, Monetary Versus Individual Non-Monetary Conditions in the UK and China.**

	b	*SE*	*t*	*P*	95% CI
Flat Fee	-12.23	3.25	-3.76	< 0.001	[-18.60, -5.86]
Norm	-5.63	3.19	-1.77	0.078	[-11.87, 0.62]
UK	40.55	3.56	11.40	< 0.001	[33.58, 47.53]
Female	1.26	1.87	0.67	0.500	[-2.41, 4.94]
Non-Binary	12.66	12.55	1.01	0.313	[-11.95, 37.27]
Education	-5.07	2.21	-2.29	0.022	[-9.41, -0.74]
Age	-2.72	1.22	-2.23	0.026	[-5.11, -0.33]
Flat Fee × UK	-44.88	4.57	-9.82	< 0.001	[-53.84, -35.91]
Norm x UK	-31.55	4.53	-6.96	< 0.001	[-40.44, -22.67]

Regression output for the multiple linear regression on the number of images rated in individual conditions in the Study 2a samples from China (*N* = 1,086 recruited on social media) and the UK (*N* = 1,067 recruited on Prolific). Flat Fee is a binary variable for whether participants were assigned to the flat fee condition. Norm is a binary variable for whether participants were assigned to the norm condition. UK is a binary variable for whether participants were part of the UK sample. Female and Non-Binary are categorical variables for gender. Education is a binary variable specifying people who have either completed or are currently pursuing an undergraduate degree or higher. Age is a continuous variable and is scaled.

Supplementary Table 8: **Study 2b, Monetary Versus Individual Non-Monetary Conditions in the US and Mexico.**

	b	*SE*	*t*	*P*	95% CI
Mexico and the US (Same Nominal Pay)					
Flat Fee	-38.24	3.30	-11.59	< 0.001	[-44.71, -31.77]
Norm	-16.42	3.32	-4.94	< 0.001	[-22.94, -9.90]
US	-23.46	3.51	-6.69	< 0.001	[-30.34, -16.58]
Female	-4.60	1.94	-2.37	0.018	[-8.40, -0.80]
Non-Binary	11.65	6.37	1.83	0.068	[-0.84, 24.15]
Education	-5.87	2.11	-2.78	0.006	[-10.02, -1.73]
Age	-1.51	1.11	-1.36	0.174	[-3.69, 0.67]
Flat Fee × US	-10.32	4.61	-2.24	0.025	[-19.37, -1.27]
Norm x US	-17.69	4.64	-3.81	< 0.001	[-26.80, -8.58]
Mexico and the US (Same Subjective Pay)					
Flat Fee	-38.22	3.35	-11.42	< 0.001	[-44.79, -31.65]
Norm	-16.39	3.37	-4.86	< 0.001	[-23.01, -9.78]
US	-16.74	3.51	-4.77	< 0.001	[-23.62, -9.86]
Female	-5.56	1.97	-2.82	0.005	[-9.43, -1.70]
Non-Binary	11.28	6.42	1.76	0.079	[-1.31, 23.87]
Education	-4.87	2.13	-2.28	0.023	[-9.05, -0.69]
Age	-2.88	1.11	-2.60	0.009	[-5.05, -0.71]
Flat Fee × US	-13.41	4.66	-2.88	0.004	[-22.55, -4.27]
Norm x US	-25.55	4.68	-5.47	< 0.001	[-34.72, -16.38]

Regression output for the multiple linear regression on the number of images rated in individual conditions in the Study 2b sample from Mexico (*N* = 1,053 recruited on Prolific) and each of the two Study 2b samples in the US: one with the same nominal pay (*N* = 1,098 on Prolific) as in Mexico and one with the same subjective [94] pay (*N* = 1,122 recruited on Prolific) as in Mexico. Flat Fee is a binary variable for whether participants were assigned to the flat fee condition. Norm is a binary variable for whether participants were assigned to the norm condition. US is a binary variable for whether participants were part of the US sample. Female and Non-Binary are categorical variables for gender. Education is a binary variable specifying people who have either completed or are currently pursuing an undergraduate degree or higher. Age is a continuous variable and is scaled.

CONDITIONS FROM DELLAVIGNA AND POPE [2] INCLUDED IN AND EXCLUDED FROM STUDY 1

Exclusion criteria are summarized in more detail in the Methods section of Study 1. To reiterate, we categorized as "monetary" pay-for-effort treatments with linear, immediate, and guaranteed piece-rates. Conditions in which the participants could not earn any additional money for themselves—either immediately or at some point in the future—were categorized as "non-monetary." Below we report every condition from the previous study [2], for which the data were originally collected, and whether these data were included in our analysis in Study 1.

***Supplementary Table 9a:* Monetary Versus Non-Monetary Treatments from DellaVigna and Pope [1] Included in Study 1.**

Monetary Treatments	Non-Monetary Treatments
Self–1 cent for 1,000 points<br>“You will be paid an extra 1 cent for every 1,000 points.”	*Flat fee Control*<br>“Your score will not affect your payment.”
Self–1 cent for 100 points<br>“You will be paid an extra 1 cent for every 100 points.”	*Unconditional Gift*<br>“In appreciation for performing this task, you will be paid a bonus of 40 cents. Your score will not affect your payment.”
Self–4 cents for 100 points<br>“You will be paid an extra 4 cents for every 100 points.”	*Please Try*<br>“Please try as hard as you can.”
Self–10 cents for 100 points<br>“You will be paid an extra 10 cents for every 100 points.”	*Social Comparison*<br>“We will show you how well you did relative to others.”
	Social Norm<br>"Many participants scored more than 2,000.”
	Charity–1 cent for 100 points<br>“The Red Cross will be given 1 cent for every 100 points.”
	Charity–10 cents for 100 points<br>“The Red Cross will be given 10 cents for every 100 points.”

Incentive treatments from a previous study [2] included in Study 1 and categorized as either monetary or non-monetary.

***Supplementary Table 9b:* Treatments from DellaVigna and Pope [1] Excluded from Study 1 and Reasons for Exclusions.**

Treatment	Reason
Probability Weighting–Low Chance, High Reward<br>"You will have a 1% chance of an extra \$1 for every 100 points."	Extra payment is not guaranteed
Probability Weighting–High Chance, Low Reward<br>"You will have a 50% chance of an extra 2 cents for every 100 points."	Extra payment is not guaranteed
Delay Discounting–2 weeks<br>"You will be paid an extra 1 cent for every 100 points (2-week delay)."	Extra payment is not immediate
Delay Discounting–4 weeks<br>"You will be paid an extra 1 cent for every 100 points (4-week delay)."	Extra payment is not immediate
Gain–40 cents<br>"You will be paid an extra 40 cents if you score at least 2,000 points."	Extra payment is not linear
Loss–40 cents<br>"You will be paid an extra 40 cents. However, you will lose this bonus unless you score."	Extra payment is not linear
Gain–80 cents<br>"You will be paid an extra 80 cents if you score at least 2,000 points."	Extra payment is not linear

Incentive treatments from a previous study [2] excluded from Study 1, together with the reasons for each exclusion (i.e., which criteria for being classified as "monetary" were not met).

ATTENTION AND COMPREHENSION CHECKS

Attention Check

Participants across studies first read the following two paragraphs:

"We are developing a machine learning image classification database, and we need your help! You are about to see a collection of images one by one, and your task is to assess whether each image contains a building or not. If you see that the image contains a building, answer YES. If you see the image does not contain a building, answer NO. If any image doesn't load, please answer NO.

Every ten images, you will be given an opportunity to be finished with the task. You can view as many images as you like. After the first 10 images completed, you will get the fee for your participation regardless of how many images you view or when you choose to quit the task."

Then, participants saw the first attention check that asked them to select the purpose of the task ("developing a machine-learning image-classification database") out of the three multiple choice options. Those who didn't pass the check were not allowed to proceed with the survey.

Comprehension Check

After being assigned to a condition and learning about the pay structure and their incentive treatment assignment, participants were asked, "How much extra pay will you get for every 10 images?" The four multiple choice answers ranged from "I will not receive any additional pay based on the number of images I complete" to "20 cents per 10 images."

Participants had two attempts to answer the questions correctly. Those who failed on both attempts were not allowed to proceed to the image-rating task.

IMAGES FROM THE EXPERIMENTAL TASK

Supplementary Image 1: **Example of an Image from the Image-Rating Task.**

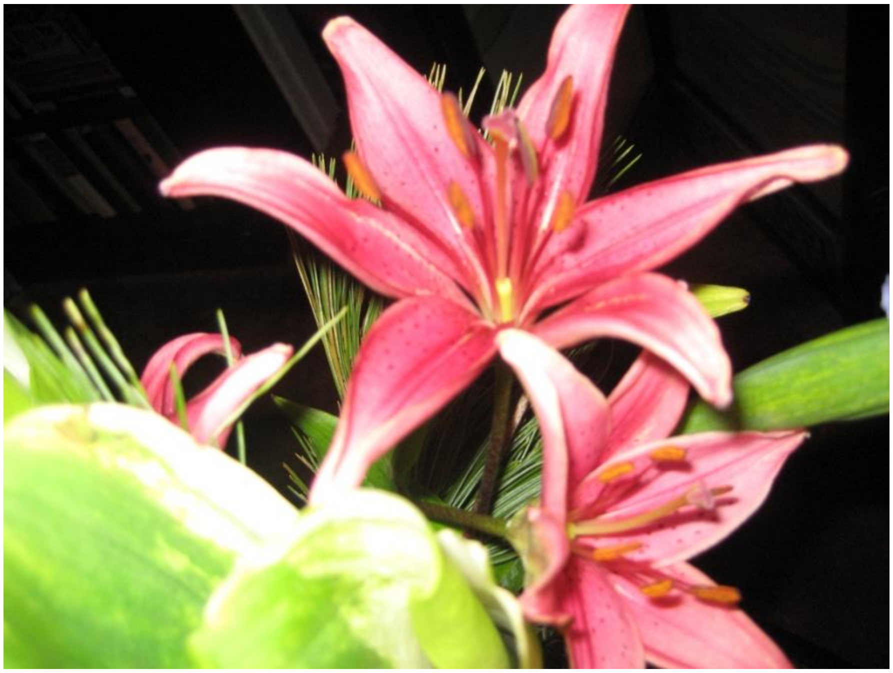

Example of an image [3] included in the image-rating task that did not contain a building.

***Supplementary Image 2:* Example of an Image from the Image-Rating Task.**

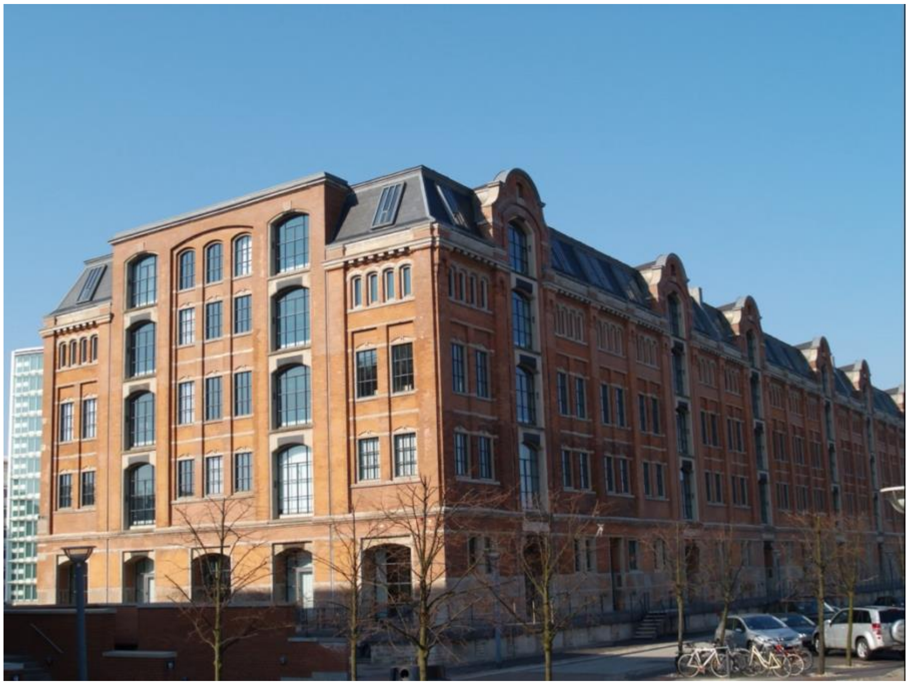

Example of an image [4] included in the image-rating task that contained a building.

MAIN EFFECTS FOR REPORTED REGRESSIONS

Below we report the main effects for the regressions on the mean number of images rated in Studies 1–4 in the monetary and (pooled) non-monetary conditions, controlling for age, gender, and education.

Study 1

We found both a main effect of treatment type, $b = 159.85$, $t(6287) = 2.91$, $P = 0.004$, 95% CI 52.35 to 267.35, and country, $b = 195.69$, $t(6287) = 5.43$, $P < 0.001$, 95% CI 125.05 to 266.34 (Fig. 1 and Table 1).

Study 2A

The main effect of incentive type for the regression on the number of images rated with incentive type, country, age, gender, and education as predictors was statistically significant, $b = 8.79$, $t(2145) = 3.11$, $P = 0.002$, 95% CI 3.25 to 14.33; the main effective of culture, $b = 2.18$, $t(2145) = 0.79$, $P = 0.429$, 95% CI -3.22 to 7.58 was not (Fig. 2 and Table 2).

Study 2B

Participants across samples rated more images in the monetary condition, $b = 27.49$, $t(2143) = 9.42$, $P < 0.001$, 95% CI 21.76 to 33.21 for the comparison between Mexico and the US sample with the same nominal pay, and $b = 27.49$, $t(2167) = 9.31$, $P < 0.001$, 95% CI 21.70 to 33.28 for the comparison between Mexico and the same subjective pay sample in the US (Fig. 3 and Table 3). Additionally, participants in the US completed fewer images than did participants in Mexico: Mexico and the US sample with the same nominal pay: $b = -37.51$, $t(2143) = -14.32$, $P < 0.001$, 95% CI -42.64 to -32.37; Mexico and the US sample with the same subjective pay: $b = -36.21$, $t(2167) = -13.87$, $P < 0.001$, 95% CI -41.33 to -31.09.

Study 2C

Participants in South Africa and the US completed more images in the monetary than non-monetary conditions, $b = 25.29$, $t(1303) = 8.78$, $P < 0.001$, 95% CI 19.64 to 30.93 for South Africa and the US sample with the same nominal pay; $b = 25.22$, $t(1303) = 8.63$, $P < 0.001$, 95% CI 19.48 to 30.95 for South Africa and the US sample with the same subjective pay (Fig. 4 and Table 4). Participants in the US completed fewer images than did participants in South Africa, $b = -8.97$ (South Africa and the US sample with the same nominal pay); $t(1303) = -2.97$, $P = 0.003$, 95% CI -14.90 to -3.04; $b = -10.78$, $t(1303) = -3.57$, $P < 0.001$, 95% CI -16.70 to -4.86 (South Africa and the US sample with the same subjective pay).

Study 3A

We found a statistically significant main effect of country, with participants in India completing more image-ratings than did participants in the US, $b = -26.03$, $t(726) = -5.71$, $P < 0.001$, 95% CI -34.99 to -17.08. We did not observe a statistically significant effect of incentive, $b = 0.83$, $t(726) = 0.19$, $P = 0.847$, 95% CI -7.57 to 9.22 (Fig. 5 and Table 5).

Study 4

A multiple linear regression on the mean number of images rated with incentive and language as predictors showed a statistically significant main effect of incentive, $b = 12.63$, $t(2061) = 5.56$, $P < 0.001$, 95% CI 8.17 to 17.09, but not language, $b = -1.74$, $t(2061) = -0.76$, $P = 0.445$, 95% CI -6.22 to 2.74 (Fig. 6 and Table 6).

STUDY 3B: IS MONEY MOTIVATING TO PEOPLE IN THE US BECAUSE OF GAMIFICATION?

One alternative explanation for the findings in Study 3a is that participants in the US found a tiny monetary incentive more motivating not because it was money, but because the task triggered their sense of competitive games. Many smartphone apps take advantage of gamification to motivate people to rate restaurants, tag photos, or correct errors by rewarding points, even without money [5]. We tested this alternative explanation in Study 3b.

We pre-registered the hypothesis, methods, and analysis. We tested whether people in the US (N = 537 recruited on Prolific; 263 female, 7 non-binary, $Mean_{\text{Age}}$ = 41.04, SD_{Age} = 12.39) would exert the same amount of effort when we rewarded them for rating each 10 additional pictures with a cent (minimal pay) or a point without any monetary value (gamification).

Results

Participants worked harder in the minimal pay (M = 29.38, SD = 31.90) than in the gamification condition (M = 18.76, SD = 19.39), Welch's $t(446.98)$ = 4.67, $P < 0.001$, $Mean_{\text{difference}}$ = 10.61, d = 0.40, 95% CI 6.15 to 15.08 (Extended Data Fig. 4). They completed 56.6% more pictures when working for cents as compared to for points.

The minimal pay condition was more cost-effective than the gamification condition, two-sided Welch's $t(471.93) = 4.37$, $P < 0.001$, $Mean_{\text{difference}} = 7.15$, $d = 0.38$, 95% CI 3.93 to 10.36 (Extended Data Fig. 4).

The results of Supplementary Study 3b show that gamification cannot not explain the effectiveness of tiny amounts of money in Study 3a. Instead, participants in the US seem to be motivated specifically by money.

Methods for Supplementary Study 3b

Pre-Registration

The study was pre-registered on March 29, 2023. The pre-registration is available on AsPredicted: https://aspredicted.org/7e2xy.pdf. We chose not to pre-register a specific hypothesis and instead delineated how the results would be interpreted based on each possible outcome of the analysis. The study did not deviate from the pre-registration. We pre-registered recruiting a total of 540 participants in the US to have 90% power to detect an effect size of f = 0.14.

Participants

We recruited the participants in the US on Prolific (N = 537). Demographic information is available in Supplementary Table 11c.

Incentive Treatments

Participants were randomly assigned to one of the two conditions: minimal pay or points (gamification). In both conditions, participants received $1.30 for participation. In the minimal pay condition, participants read that they would receive an additional 1 cent for every 10 pictures they rated. In the points (gamification) condition, participants were told that they would receive

no additional pay but would get a point for every 10 pictures they assessed. The instructions further explained: “You can treat these points as if it were a game. However, they do not lead to additional financial compensation.”

We raised the pay from 1 cent for every 20 images in Study 3a to allow participants to receive a cent (or a point) on every screen that asked them whether they wanted to continue with or quit the task (after rating every 10 images), which we thought would be more conducive to gamification.

EXCLUSION DATA AND CRITERIA FOR STUDIES 2–4

Exclusion Criteria

Among the participants who passed the attention checks, the only criteria for inclusion were that participants should (1) receive the payment code, which meant they got to the end of the survey, (2) rate at least one image, and (3) not complete the same survey more than once. Completing the task could be achieved in one of the two ways: (1) completing the whole 10 minutes of the task and timing out or (2) choosing to quit the task at one of the screens that appeared after every 10 image-ratings and asked whether participants wanted to continue to rate images. For criterion (3), we used Worker IDs to identify duplicate submissions for the same survey.

Exclusion Data

Study 2A

China

For the survey in China, we received a total of 2,029 unique submissions. Of these, 9 participants did not provide consent to participate in the study; 702 people did not pass the attention check (N = 500) or comprehension check (N = 168) or willingly quit the survey before getting to the image-rating task (N = 34). Additionally, 222 participants willingly quit the survey during the task that contained DV (i.e., not during one of the screens that allowed them to do so without penalty) and thus did not complete the task.

Out of the people who received the payment code (N = 1,096), which means they completed the whole survey, we removed the participants who did not rate any images (N = 5) or

reported being younger than 18 years old (*N* = 5). Every remaining participant completed at least one image, lived in China, and was at least 18 years old. This left us with 1,086 participants.

In China, we did not have a variable similar to one's "Worker ID." Although we did collect participants' AliPay accounts, we thought it was unreasonable to automatically classify repeating AliPay accounts as duplicates: not everyone has their own AliPay account, and it is relatively common for people to receive payments on someone else's behalf.

UK

For the survey in the UK, we received a total of 1,164 unique submissions. From these submissions, we excluded those where participants did not provide consent to participate in the study (*N* = 1). Further, 90 people failed the attention (*N* = 18) or comprehension (*N* = 72) check or willingly quit the survey before getting to the image-rating task that contained the DV (*N* = 0).

From those who received the payment code (*N* = 1,073), which means they completed the whole survey, we removed the second or further attempts by the same participant as identified by their input of the Worker IDs (*N* = 6). Every remaining participant completed at least one image, lived in the UK, and was at least 18 years old. This left us with 1,067 participants.

Study 2B

Mexico

For the survey in Mexico, we received a total of 1,268 unique submissions. Of these, 2 participants did not give consent to participate in the study. Further, 156 people failed the attention (*N* = 60) or comprehension (*N* = 94) check or willingly quit the survey before getting to the image-rating task that contained the DV (*N* = 2). Additionally, 5 participants willingly quit the survey during the task that contained the DV (i.e., not during one of the screens that allowed them to do so without penalty) and thus did not complete the task.

From those who received the payment code (*N* = 1,105), which means they completed the whole survey, we removed the second or further attempts by the same participant as identified by their input of the Worker IDs (*N* = 52). Every remaining participant completed at least one image, lived in Mexico, and was at least 18 years old. This left us with 1,053 participants.

US Same Nominal Pay

For the US survey with the same nominal pay, we received a total of 1,195 unique submissions. Of these, 79 people failed the attention (*N* = 43) or comprehension check (*N* = 35) or willingly quit the survey before getting to the image-rating task that contained the DV (*N* = 1). Additionally, 5 participants willingly quit the survey during the task that contained DV (not during one of the screens that allowed them to do so without penalty) and thus did not complete the task.

From those who received the payment code (*N* = 1,111), which means they completed the whole survey, we removed the second or further attempts by the same participant as identified by their input of the Worker IDs (*N* = 13). Every remaining participant completed at least one image, lived in the US, and was at least 18 years old. This left us with 1,098 participants.

US Same Subjective Pay

For the US survey with the same subjective pay, we received a total of 1,219 unique submissions. Of these people, 1 participant did not give consent to participate in the study. Seventy-five failed the attention (*N* = 46) or comprehension (*N* = 29) check or willingly quit the survey before getting to the image-rating task that contained the DV (*N* = 0). Additionally, 11 participants willingly quit the survey during the task that contained DV (not during one of the screens that allowed them to do so without penalty) and thus did not complete the task.

From those who received the payment code (*N* = 1,132), which means they completed the whole survey, we removed the second or further attempts by the same participant as identified by their input of the Worker IDs (*N* = 10). Every remaining participant completed at least one image, lived in the US, and was at least 18 years old. This left us with 1,122 participants.

Study 2C

South Africa

For the survey in South Africa, we received a total of 717 unique submissions. Of these, 4 participants did not provide consent to participate in the study. Fifty-one either failed the attention (*N* = 10) or comprehension (*N* = 41) check or willingly quit the survey before getting to the image-rating task that contained the DV (*N* = 0). Additionally, 6 participants willingly quit the survey during the task that contained the DV (i.e., not during one of the screens that allowed them to do so without penalty) and thus did not complete the task.

From those who received the payment code (*N* = 656), which means they completed the whole survey, we removed the second or further attempts by the same participant as identified by their input of the Worker IDs (*N* = 4). Finally, we excluded participants who did not rate a single image (*N* = 1) and those who stated that they did not currently live in South Africa (*N* = 2). Every remaining participant completed at least one image, currently lived in South Africa, and was at least 18 years old. This left us with 649 participants.

US Same Nominal Pay

For the US survey with the same nominal pay, we received a total of 700 unique submissions. Of these, 23 failed the attention (*N* = 8) or comprehension (*N* = 13) check or willingly quit the survey before getting to the image-rating task that contained the DV (*N* = 2). Additionally, 14 participants willingly quit the survey during the task that contained the DV (i.e.,

not during one of the screens that allowed them to do so without penalty) and thus did not complete the task.

From those who received the payment code (*N* = 663), which means they completed the whole survey, we removed the second or further attempts by the same participant as identified by their Worker IDs (*N* = 1). Every remaining participant completed at least one image, currently lived in the US, and was at least 18 years old. This left us with 662 participants.

US Same Subjective Pay

For the US survey with the same subjective pay, we received a total of 697 unique submissions. Of these, 1 participant did not provide consent to participate in the study. Twenty-four failed the attention (*N* = 5) or comprehension (*N* = 18) check or willingly quit the survey before getting to the image-rating task that contained the DV (*N* = 1). Additionally, 6 participants willingly quit the survey during the task that contained the DV (i.e., not during one of the screens that allowed them to do so without penalty) and thus did not complete the task.

From those who received the payment code (*N* = 666), which means they completed the whole survey, we removed the second or further attempts by the same participant as identified by their input of the Worker IDs (*N* = 2) and participants who stated that they did not currently living in the US (*N* = 2). Every remaining participant completed at least one image, currently lived in the US, and was at least 18 years old. This left us with 662 participants.

Study 3A

India

For the survey in India, we received a total of 602 unique submissions. Of these, 2 participants did not currently live in India and 10 did not give consent to participate in the study. Further, 160 participants failed the attention (*N* = 62) or comprehension check (*N* = 97) or

willingly quit the survey before rating the image (*N* = 1). Additionally, 17 participants willingly quit the survey during the task that contained the DV (i.e., not during one of the screens that allowed them to do so without penalty) and thus did not complete the task.

From those who received the payment code (*N* = 413), which means they completed the whole survey, we removed the second or further attempts by the same participant as identified by their input of the Worker IDs (*N* = 61). Every remaining participant completed at least one image, currently lived in India, and was at least 18 years old. This left us with 352 participants.

US

For the survey in the US, we received a total of 432 unique submissions. Of these, 4 participants did not give consent to participate in the study, 35 people either did not pass the attention (*N* = 16) or comprehension (*N* = 18) check or willingly quit the survey before rating a single image (*N* = 1). Additionally, 7 participants willingly quit the survey during the task that contained the DV (i.e., not during one of the screens that allowed them to do so without penalty) and thus did not complete the task.

From those who received the payment code (*N* = 386), which means they completed the whole survey, we removed the second or further attempts by the same participant as identified by their input of the Worker IDs (*N* = 4). Every remaining participant completed at least one image, currently lived in the US, and was at least 18 years old. This left us with 382 participants.

Supplementary Study 3B

For the US survey, we received a total of 566 unique submissions. Of these, we removed 23 people who failed the attention (*N* = 7) or comprehension (*N* = 15) check or willingly quit the survey before getting to the image-rating task that contained the DV (*N* = 1). Additionally, 5

participants willingly exited the survey during the task that contained the dependent variable and thus did not complete the survey.

From those who completed the whole survey and received the payment code (N = 538), we removed 1 participant who stated that they did not currently live in the US. Every remaining participant completed at least one image, currently lived in the US, and was at least 18 years old. This left us with 537 participants.

Study 4

For the survey in India, we received a total of 6,246 responses. Of these, 825 were excluded as they were identified as “Spam” by the (internal) Qualtrics quality-control mechanism. Of the remaining 5,421 unique submissions, 2,386 were removed as they were identified by either duplicate phone numbers or email addresses as second or further attempts by the same participant. This number is higher than in the previous studies, because participants were recruited on Facebook and not a crowd-sourcing website, which means that nothing prevented participants from using the survey link again, if they wanted to do so.

Of the remaining 3,035 participants, 933 people either failed the attention (N = 589) or comprehension (N = 339) check or willingly quit the survey before the image-rating task (N = 5). Additionally, 33 participants willingly quit the survey during the image-rating task (i.e., not during one of the screens that allowed them to do so without penalty) and thus did not complete the task. Finally, 2 participants completed the survey without rating any images, and 2 participants provided phone number input that was not numeric. Every remaining participant completed at least one image, currently lived in India, and was at least 18 years old. This left us with 2,065 participants.

Note Regarding Samples in Studies 2A and 3A

Some participants completed the task and received the payment code but did not press the button that officially ended the survey. We downloaded the data for Studies 2a and 3a before the responses from these participants were counted as completed by Qualtrics. On Qualtrics, such responses do not appear in the data for one week. In Study 2a, 13 participants in China and 62 participants in the UK were not included in the original dataset we used for the analyses. In Study 3a, the number of these participants was 7 in India and 1 in the US.

We only noticed the data from these participants after we had downloaded the raw Qualtrics data during the revision process to upload the raw Qualtrics files onto OSF together with the rest of the study materials. We chose not to update the results in the paper to reflect these additional participants. However, the main results reported in the paper do not change regardless of whether these "late" participants are included or excluded.

Thus, the central interaction between country and incentive type in Study 2a reported in the main text as $b = 38.40$, $t(2145) = 9.65$, $P < 0.001$, 95% CI 30.59 to 46.20 becomes $b = 38.85$, $t(2214) = 9.92$, $P < 0.001$, 95% CI 31.18 to 46.53, if these "late" participants are included. The analogous interaction in Study 3a, reported as $b = 13.77$, $t(726) = 2.31$, $P = 0.021$, 95% CI 2.09 to 25.45, becomes $b = 14.16$, $t(734) = 2.39$, $P = 0.017$, 95% CI 2.52 to 25.80.

Note Regarding the US Samples in Study 2B

After collecting the data in the US, we noticed that some participants completed both the survey with the same nominal pay and the survey with increased pay, as we ran the two surveys concurrently and could not automatically exclude participants from taking both surveys. In the main text, attempts for both surveys are included. Here, we report the key results if we only include the participants' first attempt across the two surveys.

Thus, if a participant completed the survey with the nominal pay first, we would keep this response and exclude their data from the sample with the same subjective pay. Performing these exclusions would leave us with 1,048 participants in the sample with the same nominal pay and 953 participants in the sample with the same subjective pay. None of the results reported in the main text significantly change based on whether we include or exclude these participants. The interaction between country and incentive type, controlling for age, gender, and education, reported in the main text remains statistically significant for the comparison between Mexico and the US sample with the same nominal pay, $b = 13.10$, $t(2093) = 3.20$, $P = 0.001$, 95% CI 5.07 to 21.14, as well as the US sample with increased pay, $b = 17.86$, $t(1998) = 4.16$, $P < 0.001$, 95% CI 9.44 to 26.27 (Fig. S1).

Fig. S1: **Study 2b, Monetary Versus Pooled Non-Monetary Conditions in Mexico and the US (Duplicate Participants in the US Excluded).**

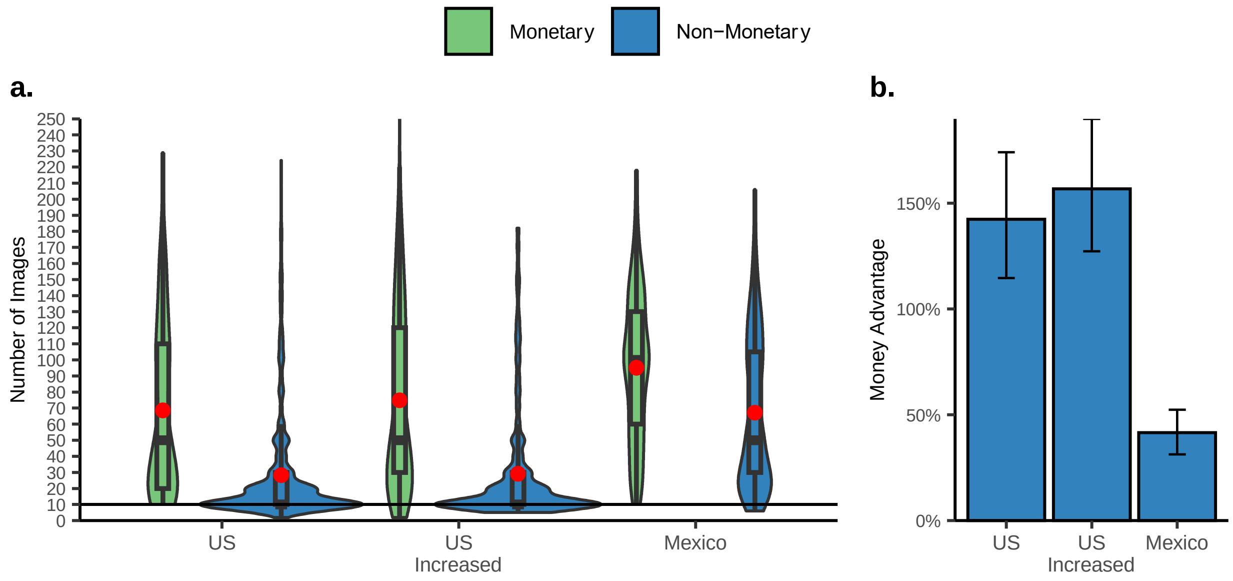

Effects of a monetary incentive (green) and pooled non-monetary treatments (flat fee and social norm; blue) in Mexico (N = 1,053 participants recruited on Prolific) and two samples in the US: one with the same nominal pay (N = 1,048 participants recruited on Prolific) as in Mexico and one with the same subjective [1] pay (N = 953 participants recruited on Prolific) as in Mexico. Second attempts from the participants in the US who completed both versions of the survey (N_{Nominal} = 50 participants, $N_{\text{Subjective}}$ = 169 participants) are excluded. Panel A shows the central tendency and distribution of effort by incentive type and country. The black line within each box represents a median, and the red dot shows a mean. Upper and lower bounds show the third and the first quartile, respectively. The whiskers represent the 1.5 times the interquartile range, with black points showing observations outside of this range. The width of each violin corresponds to the frequency of observations at any given number of images rated on the y-axis. The interaction between country and incentive type in a multiple linear regression model is statistically significant for the comparison between Mexico and the US sample with the same nominal pay, $b = 13.10$, $t(2093) = 3.20$, $P = 0.001$, 95% CI 5.07 to 21.14, as well as the US sample with the same subjective pay, $b = 17.86$, $t(1998) = 4.16$, $P < 0.001$, 95% CI 9.44 to 26.27. Panel B shows the money advantage—that is, how much more effective money is than the pooled non-monetary treatments in each sample. In Panel B, error bars are bootstrapped 95% CIs for the mean relative difference in the number of images rated in the monetary versus pooled non-monetary conditions.

MEAN EFFORT AND COST-EFFECTIVENESS IN INDIVIDUAL CONDITIONS IN STUDY 2C

Compared to the monetary incentive condition, the competition condition decreased effort more in the US than in South Africa, both in the same nominal pay sample in the US, *b* = -9.85, *t*(1301) = -1.98, *P* = 0.048, 95% CI -19.60 to -0.11 and the sample with the same subjective pay, *b* = -14.51, *t*(1301) = -2.90, *P* = 0.004, 95% CI -24.33 to -4.69. For the charity condition, the interaction between culture and incentive type was statistically significant for the same-subjective-pay sample, *b* = -16.38, *t*(1301) = -3.21, *P* = 0.001, 95% CI -26.38 to -6.38, but not for the same-nominal-pay sample, *b* = -6.09, *t*(1301) = -1.22, *P* = 0.222, 95% CI -15.86 to 3.69 (Fig. S2a and Supplementary Table 10).

In South Africa, the monetary incentive was significantly more cost-effective than the competition condition, two-sided Welch's *t*(236.70) = 3.81, $P < 0.001$, $P_{Bonf} = 0.001$, $Mean_{\text{difference}} = 7.89$, *d* = 0.39, 95 CI% 3.81 to 11.97, and the charity condition, two-sided Welch's *t*(331.79) = 9.58, $P < 0.001$, $P_{Bonf} < 0.001$, $Mean_{\text{difference}} = 14.12$, *d* = 0.91, 95 CI% 11.22 to 17.02 (Fig. S2c). In both US samples, money was similarly more cost-effective than the competition condition (Same Nominal Pay: two-sided Welch's *t*(337.20) = 6.48, $P < 0.001$, $P_{Bonf} < 0.001$, $Mean_{\text{difference}} = 14.44$, *d* = 0.62, 95 CI% 10.06 to 18.83; Increased Pay: two-sided Welch's *t*(358.34) = 7.05, $P < 0.001$, $P_{Bonf} < 0.001$, $Mean_{\text{difference}} = 8.88$, *d* = 0.66, 95 CI% 6.40 to 11.36) and the charity condition (Same Nominal Pay: two-sided Welch's *t*(468.45) = 9.21, $P < 0.001$, $P_{Bonf} < 0.001$, $Mean_{\text{difference}} = 16.32$, *d* = 0.81, 95 CI% 12.84 to 19.80; Increased Pay: two-sided Welch's *t*(474.17) = 12.34, $P < 0.001$, $P_{Bonf} < 0.001$, $Mean_{\text{difference}} = 12.22$, *d* = 1.08, 95 CI% 10.28 to 14.17).

Fig. S2: **Study 2c, Monetary Versus Individual Non-Monetary Conditions in the US and South Africa.**

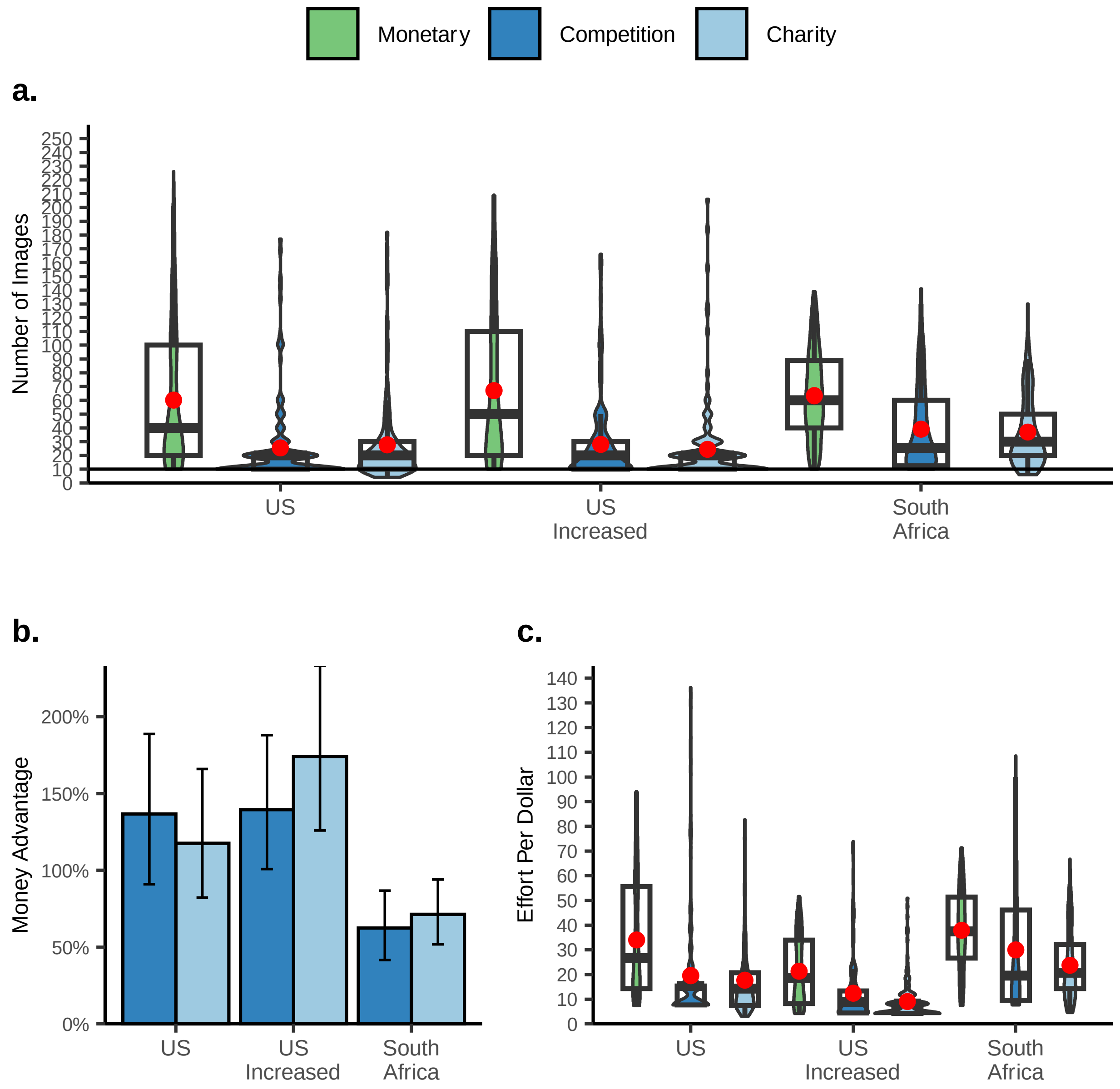

Effects of the three individual conditions: monetary (green), competition (dark blue), and charity (light blue) in South Africa (N = 649 participants recruited on Prolific) and two samples in the US: one with the same nominal pay (N = 662 participants recruited on Prolific) as in South Africa and the other with the same subjective [1] pay (N = 662 participants recruited on Prolific) as in South Africa. Panel A shows the central tendency and distribution of effort by incentive type and country. The black line within each box represents a median, and the red dot shows a mean. Upper and lower bounds show the third and the first quartile, respectively. The whiskers represent the 1.5 times the interquartile range, with black points showing observations outside of this range. The width of each violin corresponds to the frequency of observations at any given number of images rated on the y-axis. The interaction between culture and the competition condition is significant for the sample with the US sample with the same nominal pay, b = -

9.85, $t(1301) = -1.98$, $P = 0.048$, 95% CI -19.60 to -0.11, and the sample with the same subjective pay, $b = -14.51$, $t(1301) = -2.90$, $P = 0.004$, 95% CI -24.33 to -4.69. The interaction between culture and the charity condition in a multiple linear regression model is statistically significant for the comparison between South Africa and the same subjective-pay US sample, $b = -16.38$, $t(1301) = -3.21$, $P = 0.001$, 95% CI -26.38 to -6.38, but not the same-nominal-pay sample, $b = -6.09$, $t(1301) = -1.22$, $P = 0.222$, 95% CI -15.86 to 3.69. Panel B shows the money advantage—that is, how much more effective money is than each of the two non-monetary treatments in each sample. Panel C shows the central tendency and distribution of the cost-effectiveness (effort per dollar spent) by incentive type and country. Graph elements are analogous to those in Panel A, with the width of each violin corresponding to the frequency of observations at any given level of cost-effectiveness (effort per dollar spent) rated on the y-axis. The results for cost-effectiveness are summarized in the Supplementary Materials. In Panel B, error bars are bootstrapped 95% CIs for the mean relative difference in the number of images in the monetary and each of the non-monetary conditions.

Supplementary Table 10: **Study 2c, Monetary Versus Individual Non-Monetary Conditions in the US and South Africa.**

	b	*SE*	*t*	*P*	95% CI
South Africa and the US (Same Nominal Pay)					
Competition	-24.32	3.52	-6.92	< 0.001	[-31.21, -17.42]
Charity	-26.29	3.57	-7.37	< 0.001	[-33.29, -19.30]
US	-0.99	2.96	-0.33	0.738	[-6.81, 4.82]
Female	0.47	2.07	0.22	0.822	[-3.60, 4.53]
Non-Binary	-6.79	7.42	-0.92	0.360	[-21.34, 7.76]
Education	-6.95	2.08	-3.35	0.001	[-11.03, -2.88]
Age	-3.12	1.16	-2.69	0.007	[-5.40 -0.84]
Competition × US	-9.85	4.97	-1.98	0.048	[-19.60, -0.11]
Charity x US	-6.09	4.98	-1.22	0.222	[-15.86, 3.69]
South Africa and the US (Same Subjective Pay)					
Competition	-24.22	3.57	-6.79	< 0.001	[-31.22, -17.22]
Charity	-26.25	3.62	-7.25	< 0.001	[-33.35, -19.15]
US	4.64	3.02	1.53	0.125	[-1.29, 10.57]
Female	-2.13	2.09	-1.02	0.309	[-6.23, 1.98]
Non-Binary	-12.97	7.92	-1.64	0.102	[-28.50, 2.57]
Education	-4.27	2.11	-2.02	0.044	[-8.42, -0.12]
Age	-1.11	1.19	-0.93	0.351	[-3.43, 1.22]
Competition × US	-14.51	5.01	-2.90	0.004	[-24.33, -4.69]
Charity x US	-16.38	5.10	-3.21	0.001	[-26.38, -6.38]

Regression output for the multiple linear regression on the number of images rated in the individual conditions in the Study 2c sample from South Africa (N = 649 recruited on Prolific) and each of the two Study 2c samples in the US: one with the same nominal pay (N = 662 recruited on Prolific) as in South Africa and the other with the same subjective [1] pay (N = 662 recruited on Prolific) as in South Africa. Competition is a binary variable for whether participants were assigned to the competition condition. Charity is a binary variable for whether participants were assigned to the charity condition. US is a binary variable for whether participants were part of the US sample. Female and Non-Binary are categorical variables for gender. Education is a binary variable specifying people who had either completed or were pursuing an undergraduate degree or higher at the time of the experiment. Age is a continuous variable and is scaled.

PROBABILITY OF QUITTING THE TASK AT THE FIRST OPPORTUNITY IN STUDIES 2A–4

As an additional measure of effort, we looked at the proportion of participants who chose to quit the task without losing the base pay for participation when provided with the first opportunity to do so. Everyone in our study was explicitly instructed that they could quit the task without losing the base pay for participation after rating only 10 images.

Study 2A

Unsurprisingly, few people regardless of culture quit the task immediately when presented with a pay-for-effort incentive (8.0% in China vs. 4.6% in the UK). However, in the non-monetary conditions, 34.3% of participants in the UK quit the task at the first opportunity, while only 14.2% of participants in China did so. When we conducted a logistic regression on the probability of quitting after rating exactly 10 images, we found a statistically significant interaction between incentive type and country, $b = -1.73$, $z = -5.03$, $P < 0.001$, OR = 0.18, 95% CI 0.09 to 0.35.

There was a statistically significant main effect of incentive type, with the participants quitting less frequently when offered a monetary bonus, $b = -0.66$, $z = -2.95$, $P = 0.003$, OR = 0.52, 95% CI 0.33 to 0.79, and country, with the UK participants being more likely to quit, $b = 1.30$, $z = 8.14$, $P < 0.001$, OR = 3.68, 95% CI 2.69 to 5.04.

Study 2B

Across samples, a relatively small proportion of participants quit the task immediately when presented with a pay-for-effort incentive, especially in Mexico (2.6% in Mexico; 15.1% in the US sample with the sample nominal pay and 13.3% in the US sample with the same subjective pay). However, when there was no monetary incentive to continue, 51.2% of US

participants in the same nominal pay sample and 54.5% in the increased pay sample exited the task at the first opportunity. In Mexico, 91.7% continued even when they could have quit early and received the same total pay for their work.

A logistic regression was used to analyze the role of incentive type and culture on the probability of quitting at the first opportunity without losing the base pay. Controlling for age, gender, and education, the interaction between incentive type and culture on the probability of quitting at 10 images was not statistically significant for the comparison between Mexico and the US sample with the same nominal pay, $b = -0.57$, $z = -1.41$, $P = 0.157$, OR = 0.57, 95% CI 0.27 to 1.32, but statistically significant for the US sample with the same subjective pay, $b = -0.85$, $z = -2.11$, $P = 0.034$, OR = 0.43, 95% CI 0.20 to 0.99.

Study 2C

In South Africa, 4.2% of participants quit at the first opportunity in the monetary condition. In the US, that number was 18.5% in the sample with the same nominal pay as in South Africa and 16.0% in the sample with the same subjective pay. Across the two non-monetary conditions, 21.8% of people in South Africa quit after rating 10 images and 43.8% and 45.5% of people in the two US samples, respectively.

When we conducted a logistic regression on the probability of quitting the task at the first opportunity, we found that participants were less likely to quit the task in the monetary condition, $b = -1.87$, $z = -6.11$, $P < 0.001$, OR = 0.15, 95% CI 0.08 to 0.27 for the comparison between South Africa and the US sample with the same nominal pay (and $b = -1.88$, $z = -6.13$, $P < 0.001$, OR = 0.15, 95% CI 0.08 to 0.27 for the comparison between South Africa and the US sample with the same subjective pay). Participants in the US were more likely to quit the task than participants in South Africa, $b = 1.10$, $z = 5.88$, $P < 0.001$, OR = 3.00, 95% CI 2.09 to 4.34

for the sample in the US with the same nominal pay (and $b = 1.17$, $z = 6.35$, $P < 0.001$, OR = 3.20, 95% CI 2.25 to 4.62 for the sample with the same subjective pay). The interaction was not statistically significant for the comparison between the South African sample and the US sample with the same nominal pay, $b = 0.59$, $z = 1.67$, $P = 0.094$, OR = 1.81, 95% CI 0.92 to 3.73, and between South Africa and the US sample with the same subjective pay, $b = 0.40$, $z = 1.11$, $P = 0.266$, OR = 1.49, 95% CI 0.75 to 3.09, showing that participants in the US were more likely to quit in both types of conditions and not in the non-monetary conditions in particular.

Study 3A

Unlike in all the previous studies, in Study 3a a higher proportion of people in the US quit at the first opportunity (35.4% in the minimal pay condition compared to 14.9% in India) in the monetary condition. In the norm condition, 47.4% of people in the US quit the task compared to 18.1% in India. When we ran a multiple linear regression and controlled for age, gender, and education, we found a statistically significant main effect of country, $b = 1.63$, $z = 6.11$, $P < 0.001$, OR = 5.09, 95% CI 3.05 to 8.68, showing that people in the US were more likely to quit the task after 10 images regardless of condition, but neither a main effect of incentive, $b = -0.27$, $z = -0.92$, $P = 0.359$, OR = 0.77, 95% CI 0.43 to 1.35, nor their interaction, $b = -0.24$, $z = -0.66$, $P = 0.510$, OR = 0.79, 95% CI 0.39 to 1.60.

Supplementary Study 3B

A logistic regression on quitting after rating ten images showed that people in the minimal pay condition were less likely to quit at the first opportunity (46.5%) compared to people in the points condition (63.9%), $b = -0.71$, $z = -4.03$, $P < 0.001$, OR = 0.49, 95% CI 0.35 to 0.69.

Study 4

In the monetary condition, 7.9% of participants who completed the study in English and 9.5% who completed the study in Hindi exited the task after rating 10 images—the first time they could do so without losing their base pay. In the norm condition, those numbers were 24.9% in English and 25.3% in Hindi. A logistic linear regression on the likelihood of quitting at the first opportunity was not statistically significant, $b = -0.17$, $z = -0.66$, $P = 0.509$, OR = 0.84, 95% CI 0.50 to 1.41. The regression further showed a statistically significant main effect of incentive, $b = -1.18$, $z = -6.54$, $P < 0.001$, OR = 0.31, 95% CI 0.21 to 0.44, but not of language, $b = -0.02$, $z = -0.16$, $P = 0.871$, OR = 0.98, 95% CI 0.74 to 1.29.

DEMOGRAPHIC VARIABLES

Supplementary Table 11a: **Demographic Variables for Study 1.**

	M	*SD*	*N*
US			
Monetary Incentive	36.7%		5526
Female	57.8%		5526
Education	50.6%		5526
Age Category	2.62	1.29	5526
India			
Monetary Incentive	35.8%		768
Female	32.2%		768
Education	84.0%		768
Age Category	2.41	1.02	768

Monetary Incentive is a binary variable for whether participants were in one of the monetary (pay-for-effort) conditions. US is a binary variable for whether participants were part of the US sample. Female is a categorical variable for gender. Age is measured on a scale, where 1 = “18–24”; 2 = “25–30”; 3 = “31–40”; 4 = “41–50”; 5 = “51–64”; 6 = “65 or higher.” Education is a binary variable specifying people who had either completed or were pursuing an undergraduate degree or higher at the time of the experiment.

***Supplementary Table 11b:* Demographic Variables for Studies 2a–c.**

	M	*SD*	*N*
Study 2a: China			
Monetary Incentive	32.0%		1086
Female	57.6%		1086
Non-Binary	0%		1086
Education	83.4%		1086
Current Student	72.7%		1086
No Internet Issues	35.5%		1086
Time to Load Images	5.11	12.85	1086
Age	23.31	5.78	1086
Subjective SES	4.99	1.65	1070
Study 2a: UK			
Monetary Incentive	34.4%		1067
Female	51.0%		1067
Non-Binary	1.1%		1067
Education	63.4%		1067
Current Student	11.2%		1067
Lives in a City	49.9%		1067
No Internet Issues	87.6%		1067
Time to Load Images	1.49	2.19	1067
Age	40.04	13.60	1067
Subjective SES	4.96	1.73	1067
Income	48219.11	32715.32	963

	M	*SD*	*N*
Study 2b: Mexico			
Monetary Incentive	32.6%		1053
Female	50.9%		1053
Non-Binary	2.5%		1053
Education	77.9%		1053
Current Student	59.9%		1053
Lives in a City	95.5%		1053
No Internet Issues	70.1%		1053
Time to Load Images	1.82	4.53	1053
Age	24.51	5.43	1053
Subjective SES	5.75	1.45	1053
Study 2b: US (Same Nominal Pay)			
Monetary Incentive	33.2%		1098
Female	59.4%		1098
Non-Binary	2.2%		1098
Education	65.5%		1098
Current Student	17.3%		1098
Lives in a City	68.4%		1098
No Internet Issues	87.0%		1098
Time to Load Images	1.72	2.14	1098
Age	37.28	13.77	1098
Subjective SES	5.04	1.88	1098
Income	71070.73	48702.77	1018

	M	*SD*	*N*
Study 2b: US (Same Subjective Pay)			
Monetary Incentive	33.6%		1122
Female	60.1%		1122
Non-Binary	2.2%		1122
Education	64.5%		1122
Current Student	19.9%		1122
Lives in a City	68.8%		1122
No Internet Issues	86.9%		1122
Time to Load Images	1.78	4.87	1122
Age	36.13	13.31	1122
Subjective SES	4.97	1.81	1122
Income	69599.24	47404.77	1048
Study 2c: South Africa			
Monetary Incentive	51.2%		649
Female	48.7%		649
Non-Binary	0.9%		649
Education	60.1%		649
Current Student	37.6%		649
Lives in a City	75.3%		649
No Internet Issues	79.4%		649
Time to Load Images	3.02	6.21	649
Age	28.29	7.45	649
Subjective SES	4.93	1.67	649

	M	*SD*	*N*
Income	26354.58	30553.08	502
Study 2c: US (Same Nominal Pay)			
Monetary Incentive	49.7%		662
Female	48.0%		662
Non-Binary	3.0%		662
Education	58.5%		662
Current Student	16.2%		662
Lives in a City	67.2%		662
No Internet Issues	85.2%		662
Time to Load Images	1.82	2.98	662
Age	36.91	12.62	662
Subjective SES	4.82	1.92	662
Income	72777.78	49359.34	621
Study 2c: US (Same Subjective Pay)			
Monetary Incentive	50.2%		662
Female	48.8%		662
Non-Binary	2.6%		662
Education	59.7%		662
Current Student	15.3%		662
Lives in a City	71.1%		662
No Internet Issues	83.1%		662
Time to Load Images	1.66	1.59	662
Age	36.27	12.52	662
Subjective SES	4.88	1.83	662

	M	*SD*	*N*
Income	70586.78	48008.63	605

Monetary Incentive is a binary variable for whether the participants were assigned to the monetary condition. Female and Non-Binary are categorical variables for gender. Education is a binary variable specifying people who had either completed or were pursuing an undergraduate degree or higher at the time of the experiment. Current Student is a binary variable for whether participants reported being currently enrolled as students. Lives in a City is a binary variable for whether participants reported living in a medium-sized or large city. No Internet Issues is a binary variable for participants who reported having no problems with their Internet connection during the study. Time to Load Images is a scaled, continuous variable for self-reported time it took for each image to load. Age is a continuous variable for age, ranging from 18 to 70 (and above), the latter coded as 70 (for China, Age is a continuous variable for age, ranging from 18 to 60 and above, the latter coded as 60). Subjective SES is a continuous variable for subjective socioeconomic status, ranging from 0 to 10. Income is a categorical variable, with "Income: Less than $10,000" coded as $5,000; "$10,000 to $19,999" coded as $15,000; "$20,000 to $29,999" coded as $25,000; "$30,000 to $39,999" coded as $35,000; "$40,000 to $49,999" coded as $45,000; "$50,000 to $59,999" coded as $55,000; "$60,000 to $69,999" coded as $65,000; "$70,000 to $79,999" coded as $75,000; "$80,000 to $89,999" coded as $85,000; "$90,000 to $99,999" coded as $95,000; "$100,000 to $149,999" coded as $125,000; "$150,000 or more" coded as $175,000; "Not Employed," "Student," and "Retired" were coded as missing values for the purpose of calculating the mean and standard deviation of income in each sample.

Supplementary Table 11c: **Demographic Variables for Study 3a and Supplementary Study 3b.**

	M	*SD*	*N*
Study 3a: India			
Monetary Incentive	49.7%		352
Female	23.6%		352
Non-Binary	0%		352
Education	95.7%		352
Current Student	2.6%		352
Lives in a City	79.5%		352
No Internet Issues	75.6%		352
Time to Load Images	3.28	5.46	352
Age	35.79	8.73	352
Subjective SES	6.07	1.64	352
Income	21911.31	25882.01	327
Study 3a: US			
Monetary Incentive	50.3%		382
Female	51.6%		382
Non-Binary	3.7%		382
Education	55.0%		382
Current Student	14.4%		382
Lives in a City	64.4%		382
No Internet Issues	81.2%		382
Time to Load Images	2.00	6.37	382
Age	37.76	13.99	382

	M	*SD*	*N*
Subjective SES	4.73	1.88	382
Income	66256.98	45403.09	358
Supplementary Study 3b: US			
Monetary Incentive	50.5%		537
Female	49.0%		537
Non-Binary	1.3%		537
Education	66.9%		537
Current Student	8.4%		537
Lives in a City	66.7%		537
No Internet Issues	88.8%		537
Time to Load Images	1.68	1.77	537
Age	41.04	12.39	537
Subjective SES	4.96	1.84	537
Income	78174.90	48343.96	526

Monetary Incentive is a binary variable for whether the participants were assigned to the monetary (minimal pay) condition. Female and Non-Binary are categorical variables for gender. Education is a binary variable specifying people who had either completed or were pursuing an undergraduate degree or higher at the time of the experiment. Current Student is a binary variable for whether participants reported being currently enrolled as students. Lives in a City is a binary variable for whether participants reported living in a medium-sized or large city. No Internet Issues is a binary variable for participants who reported having no problems with their Internet connection during the study. Time to Load Images is a scaled, continuous variable for self-reported time it took for each image to load. Age is a continuous variable for age, ranging from 18 to 70 (and above), the latter coded as 70. Subjective SES is a continuous variable for subjective socioeconomic status, ranging from 0 to 10. Income is a categorical variable, with “Income: Less than $10,000” coded as $5,000; “$10,000 to $19,999” coded as $15,000; “$20,000 to $29,999” coded as $25,000; “$30,000 to $39,999” coded as $35,000; “$40,000 to $49,999” coded as $45,000; “$50,000 to $59,999” coded as $55,000; “$60,000 to $69,999” coded as $65,000; “$70,000 to $79,999” coded as $75,000; “$80,000 to $89,999” coded as $85,000; “$90,000 to $99,999” coded as $95,000; “$100,000 to $149,999” coded as $125,000; “$150,000 or more” coded as $175,000; “Not Employed,” “Student,” and “Retired” were coded as missing values for the purpose of calculating the mean and standard deviation of income in each sample.

***Supplementary Table 11d:* Demographic Variables for Study 4.**

	M	*SD*	*N*
	Study 4		
Monetary Incentive	50.1%		2065
English	49.6%		2065
Female	14.2%		2013
Non-Binary	0.1%		2013
Education	81.2%		2009
Current Student	77.4%		2013
Lives in a City	65.8%		2017
No Internet Issues	59.6%		2020
Time to Load Images	4.09	8.37	2019
Age	24.87	4.90	2013
Subjective SES	5.99	2.15	2009
Income (INR)	338768.12	320456.21	1104

Monetary Incentive is a binary variable for whether participants were in one monetary condition. English is a binary variable for whether the participants were assigned to complete the study in English (as compared to in Hindi). Female and Non-Binary are categorical variables for gender. Education is a binary variable specifying people who had either completed or were pursuing an undergraduate degree or higher at the time of the experiment. Current Student is a binary variable for whether participants reported being enrolled as students at the time of the experiment. Lives in a City is a binary variable for whether participants reported living in a medium-sized or large city. No Internet Issues is a binary variable for participants who reported having no problems with their Internet connection during the study. Time to Load Images is a scaled, continuous variable for self-reported time it took for each image to load. Age is a continuous variable for age, ranging from 18 to 70 (and above), the latter coded as 70. Subjective SES is a continuous variable for subjective socioeconomic status, ranging from 0 to 10. Income is a categorical variable, with “Income: Less than ₹2.5 lakhs” coded as ₹125,000; “₹2.5 lakhs to ₹4.9 lakhs” coded as ₹375,000; “₹5 lakhs to ₹9.9 lakhs” coded as ₹750,000; “₹10 lakhs to ₹14.9 lakhs” coded as ₹1,250,000; “₹15 lakhs and above” coded as ₹1,750,000; “Not working,” “Student,” and “Retired” were coded as missing values for the purpose of calculating the mean and standard deviation of income in each sample.

BECKER-DEGROOT-MARSHAK (BDM) [2] PROCEDURE FOR ESTABLISHING PAY EQUIVALENCE

Study 2B

A separate group of participants in the pre-test (*N* = 222) completed 2 image-ratings to get a sense of the task. Then, they saw a series of choices that asked them whether they would complete 10 minutes of this task for a specified amount of money ranging from $0.10 to $6.00. They considered each pay amount separately and were told that one of their choices would be randomly implemented.

If they said “yes” to the randomly chosen amount of money, they would have to complete the whole task; if they said “no,” they would not have to continue with the task but also not get extra payment. For example, if a participant said “yes” to the 80-cent question and that was the money amount that was then randomly chosen, that participant would have to complete the task and get the extra 80 cents. If they said no, they would proceed to the end of the survey and not get the extra 80 cents.

The method resulted in $0.99 expected pay for Prolific workers from the US (*SD* = 0.73) and $0.82 for Prolific workers in Mexico (*SD* = 0.87). We used these two numbers to calculate an approximate ratio of 5:6 between the two countries. Said differently, to allow for approximately the same subjective pay in the US, we would have to increase the pay in the US by 20% as compared to that in Mexico. After allowing for additional time needed for study apart from the picture task itself (such as consent and demographics), we decided to pay $1.30 in Mexico and the “Same Nominal Pay” sample in the US and $1.59 in the “Same Subjective (Increased Pay)” sample in the US. We also adjusted the additional per-piece payments in the

monetarily incentivized condition. In the increased pay sample in the US, people received 6 cents per every 10 images rated (versus 5 cents in the other two samples).

Study 2C

A total of 209 participants on Prolific from the US (*N* = 116) and South Africa (*N* = 93) passed the attention checks and proceeded to the questions that asked them how much compensation they would need to complete a 10-minute image-rating task. The procedure was identical to that in Study 2b.

Out of people who passed the attention check, 2 people in the US and 9 people in South Africa provided nonlinear responses (e.g., said they would work for $1.00 but not for $2.00). We excluded them from the equivalence calculations. The mean "requested" compensation was $1.63 in the US (*SD* = 0.96) and $0.89 (*SD* = 0.67) in South Africa. Thus, the ratio of anticipated pay for these samples was approximately 9:5. In South Africa and in the nominal pay sample in the United States, we retained the same pay as in the previous studies: $1.30 base pay for participation, and a 5-cent bonus per rating 10 images in the monetary condition. In the sample in the US with the same subjective pay as in South Africa, we increased the pay to $2.25, with a 9-cent bonus in the monetary condition.

Note on Purchasing Power Parity

While we relied on a procedure that has been extensively used for this purpose in previous research [1], there are multiple ways to calculate the subjective equivalence of pay between different countries. One can argue that the difference in pay between Mexico and the US sample with increased pay was too small. For instance, if we were to instead rely on the Organization for Economic and Cooperation Development 2023 conversion rates for PPP

between US and Mexico to establish subjective equivalence, we would have paid participants in the US twice as much as participants in Mexico [6].

However, given the fact that neither a 20% increase in base pay (Study 2b) nor an 80% increase (Study 2c) had a statistically significant effect on effort provision in the conditions where no monetary incentive was provided, we find it unlikely that raising solely the base pay will increase the amount of effort American participants provide in the non-monetary conditions. In fact, the interaction coefficients in both Study 2b and Study 2c are larger for the comparison with the increased (versus same nominal) pay sample, and thus it is probable that choosing an even steeper conversion rate would have only further amplified the cultural differences that we observe.

ADDITIONAL CONTROLS

Repeating the Analyses with Interactions Between Country and Each Predictor Variable

Following the methods proposed by Bandiera and colleagues [7] and suggestions from an anonymous reviewer, we first repeated the analyses in the main text with interactions between country and each of the principal demographic predictor variables for all the main analyses that involved comparisons across samples. The results are presented in Supplementary Tables 12a and 12b.

Supplementary Table 12a: **Studies 2a–c, Mean Effort in Monetary Versus Pooled Non-Monetary Incentive Conditions, with Interactions Between Country and Predictor Variables.**

	b	*SE*	*t*	*P*	95% CI
Study 2a: China and the UK					
Monetary Incentive	8.88	2.83	3.14	0.002	[3.34, 14.42]
UK	5.09	4.69	1.09	0.278	[-4.11, 14.29]
Female	5.03	2.68	1.88	0.060	[-0.21, 10.28]
Non-Binary	8.76	12.69	0.69	0.490	[-16.12, 33.65]
Education	-3.55	3.93	-0.91	0.365	[-11.25, 4.14]
Age	1.97	3.36	0.59	0.558	[-4.62, 8.57]
Monetary Incentive × UK	38.36	3.98	9.64	< 0.001	[30.56, 46.16]
Female × UK	-7.73	3.78	-2.04	0.041	[-15.15, -0.31]
Education × UK	-1.40	4.82	-0.29	0.772	[-10.84, 8.05]
Age × UK	-5.35	3.61	-1.48	0.139	[-12.44, 1.73]
Study 2b: Mexico and the US (Same Nominal Pay)					
Monetary Incentive	27.05	2.92	9.28	< 0.001	[21.33, 32.77]
US	-41.27	4.83	-8.54	< 0.001	[-50.75, -31.80]
Female	-12.22	2.79	-4.38	< 0.001	[-17.69, -6.76]
Non-Binary	9.86	8.94	1.10	0.271	[-7.68, 27.40]
Education	-2.85	3.30	-0.86	0.388	[-9.31, 3.61]
Age	-2.75	3.13	-0.88	0.378	[-8.88, 3.38]
Monetary Incentive × US	14.45	4.07	3.55	< 0.001	[6.48, 22.43]
Female × US	14.36	3.93	3.66	< 0.001	[6.66, 22.07]
Non-Binary × US	4.92	12.93	0.38	0.704	[-20.44, 30.28]
Education × US	-5.16	4.34	-1.19	0.234	[-13.67, 3.35]
Age × US	1.53	3.35	0.46	0.648	[-5.04, 8.10]
Study 2b: Mexico and the US (Same Subjective Pay)					
Monetary Incentive	27.05	2.95	9.17	< 0.001	[21.26, 32.84]
US	-40.74	4.86	-8.38	< 0.001	[-50.28, -31.21]
Female	-12.22	2.82	-4.33	< 0.001	[-17.76, -6.69]

	b	*SE*	*t*	*P*	95% CI
Non-Binary	9.86	9.05	1.09	0.276	[-7.90, 27.61]
Education	-2.85	3.34	-0.85	0.393	[-9.39, 3.69]
Age	-2.64	3.03	-0.87	0.384	[-8.58, 3.30]
Monetary Incentive × US	20.04	4.09	4.90	< 0.001	[12.01, 28.06]
Female × US	13.30	3.98	3.34	< 0.001	[5.50, 21.11]
Non-Binary × US	3.42	12.97	0.26	0.792	[-22.01, 28.85]
Education × US	-4.55	4.37	-1.04	0.298	[-13.12, 4.02]
Age × US	-0.05	3.26	-0.02	0.988	[-6.45, 6.35]
Study 2c: South Africa and the US (Same Nominal Pay)					
Monetary Incentive	25.15	2.88	8.75	< 0.001	[19.51, 30.79]
US	-11.49	4.36	-2.63	0.009	[-20.05, -2.93]
Female	-3.89	2.92	-1.33	0.183	[-9.63, 1.84]
Non-Binary	-13.33	15.10	-0.88	0.377	[-42.95, 16.29]
Education	-4.09	2.97	-1.38	0.169	[-9.91, 1.74]
Age	1.24	2.17	0.57	0.569	[-3.03, 5.50]
Monetary Incentive × US	8.42	4.06	2.08	0.038	[0.46, 16.38]
Female × US	8.59	4.13	2.08	0.038	[0.48, 16.70]
Non-Binary × US	9.32	17.30	0.54	0.590	[-24.63, 43.27]
Education × US	-5.18	4.15	-1.25	0.212	[-13.33, 2.97]
Age × US	-5.67	2.52	-2.24	0.025	[-10.62, -0.71]
Study 2c: South Africa and the US (Same Subjective Pay)					
Monetary Incentive	25.15	2.93	8.59	< 0.001	[19.40, 30.90]
US	-13.29	4.43	-3.00	0.003	[-21.98, -4.61]
Female	-3.89	2.98	-1.31	0.191	[-9.73, 1.95]
Non-Binary	-13.33	15.38	-0.87	0.386	[-43.51, 16.84]
Education	-4.09	3.03	-1.35	0.177	[-10.02, 1.85]
Age	1.22	2.18	0.56	0.576	[-3.06, 5.49]
Monetary Incentive × US	15.69	4.13	3.80	< 0.001	[7.60, 23.79]
Female × US	3.58	4.19	0.85	0.393	[-4.64, 11.81]
Non-Binary × US	1.00	17.97	0.06	0.956	[-34.25, 36.24]

	b	*SE*	*t*	*P*	95% CI
Education × US	0.14	4.25	0.03	0.973	[-8.19, 8.47]
Age × US	-3.08	2.54	-1.21	0.225	[-8.06, 1.90]

Regression output for the linear regression models on the number of images rated in the samples from Studies 2a–c for each sample comparison pair, with interactions between country and each predictor variable added. Monetary Incentive is a binary variable for whether participants were in one of the monetary conditions. UK and US are binary variable for whether participants were part of the UK or one of the US samples, respectively. Female and Non-Binary are categorical variables for gender. Education is a binary variable specifying people who had either completed or were pursuing studies at the undergraduate level or higher at the time of the experiment. Age is a continuous variable and is scaled.

Supplementary Table 12b: **Study 3a, Mean Effort in Monetary Versus Pooled Non-Monetary Incentive Conditions, with Interactions Between Country and Predictor Variables.**

	b	*SE*	*t*	*P*	95% CI
		Study 3a: India and the US			
Monetary Incentive	1.47	4.29	0.34	0.732	[-6.96, 9.90]
US	-17.00	11.44	-1.49	0.138	[-39.45, 5.45]
Female	3.54	5.04	0.70	0.483	[-6.35, 13.43]
Non-Binary	19.52	11.19	1.74	0.081	[-2.44, 41.49]
Education	3.47	10.61	0.33	0.744	[-17.36, 24.30]
Age	4.52	2.91	1.55	0.122	[-1.21, 10.24]
Monetary Incentive × US	13.12	5.96	2.20	0.028	[1.43, 24.81]
Female × US	-1.76	6.56	-0.27	0.788	[-14.65, 11.12]
Education × US	-9.21	11.39	-0.81	0.419	[-31.57, 13.16]
Age × US	-6.67	3.40	-1.96	0.050	[-13.34, 0.00]

Regression output for the linear regression models on the number of images rated in the Study 3a samples from India (N = 352) and the US (N = 382), with interactions between country and each predictor variable added. Monetary Incentive is a binary variable for whether participants were assigned to the monetary (minimal pay) condition. US is a binary variable for whether participants were part of the US sample. Female and Non-Binary are categorical variables for gender. Education is a binary variable specifying people who had either completed or were pursuing studies at the undergraduate level or higher at the time of the experiment. Age is a continuous variable and is scaled.

Repeating the Analyses in Studies 2a–c and 3a with Additional Controls

For Study 2c, we pre-registered controlling for age, gender, and education only. However, the samples in South Africa and the US differed on some other demographic dimensions, such as the proportion of then-current students and that of people living in cities (as opposed to towns and villages). Similarly, we did not pre-register controlling for quality of the Internet connection in Study 2c. However, it is possible that having a slow connection would have prevented participants from rating more images, and that our samples differed in their Internet speeds.

Thus, in all the studies with between-culture comparisons for which we collected data (Studies 2a–c and 3a), we re-ran the regressions on the mean number of images rated with additional controls. We wanted to test whether the principal interaction between culture and incentive type (monetary or non-monetary) would remain robust. None of the main results changed. We report the regression outputs in Supplementary Tables 13a and 13b.

Additional Demographic Variables

Student Status and Living in a City

We added to the regression model binary variables for (1) whether participants were then-students and (2) whether they lived in a medium- or large-sized city.

Subjective SES

We also included participants' self-reported subjective SES, measured on the 0–10 "ladder" scale, where 0 = "0 (bottom) and 10 = "10 (top)." Participants saw an image of a ladder and were asked, "Think of this ladder as representing people's social status in your country. At the top of the ladder are the people who are the best off—those who have the most money, the

most education, and the most respected jobs. At the bottom are the people who are the worst off—who have the least money, the least education, and the least respected jobs or no job. Where would you place yourself on this ladder compared to people in your country?"

Income

We measured income on a scale that included non-numerical options such as "Not currently employed," "Retired," and "Student." In Studies 2a–c, 3a, and Supplementary Study 3b, numerical ranges for the income question were "Income: Less than $10,000" coded as $5,000; "$10,000 to $19,999" coded as $15,000; "$20,000 to $29,999" coded as $25,000; "$30,000 to $39,999" coded as $35,000; "$40,000 to $49,999" coded as $45,000; "$50,000 to $59,999" coded as $55,000; "$60,000 to $69,999" coded as $65,000; "$70,000 to $79,999" coded as $75,000; "$80,000 to $89,999" coded as $85,000; "$90,000 to $99,999" coded as $95,000; "$100,000 to $149,999" coded as $125,000; and "$150,000 or more" coded as $175,000.

We did not measure income as well as we could have. First—in all studies except for Study 4—we employed the scale we use by default in the US, with the lowest cut-off being $10,000 per year. $10,000 is higher than the national net income per capita of all four non-WEIRD countries in our samples [8].

Second, in China, we asked about income as a text-entry question instead, which made analysis unfeasible. Third, in Mexico, we did not specify whether "$" refers to the American dollar or the Mexican peso, which led to uninterpretable data. For these reasons, we did not include descriptive statistics for income for the samples from China and Mexico in the Demographic Variables section. For the same reasons, in Study 2a, we did not include income in

the regressions with additional controls, and in Study 2b, we included only non-numerical options (“Not currently employed,” “Retired,” and “Student”).

Internet Connection

The first Internet variable asked participants, “Did you have any internet connection/speed issues during the study?” and was measured on a scale from 1 (“No issues at all”) to 5 (“Lots of Issues”). We then created a binary variable called “No Internet Issues,” where 1 = 1 (“No issues at all”) and 0 = all other values of the variable.

The second variable, “Time to Load Images,” asked, “On average, how many seconds did it take for the picture to load completely? You may include up to 1 decimal point.” Participants were then free to input any number below 120 (2 minutes).

***Supplementary Table 13a:* Studies 2a–c, Mean Effort in Monetary Versus Pooled Non-Monetary Incentive Conditions, with Additional Controls.**

	b	*SE*	*t*	*P*	95% CI
Study 2a: China and the UK					
Monetary Incentive	8.37	2.85	2.94	0.003	[2.78, 13.96]
UK	1.32	3.13	0.42	0.674	[-4.83, 7.46]
Female	1.01	1.91	0.53	0.597	[-2.73, 4.75]
Non-Binary	9.87	12.67	0.78	0.436	[-14.98, 34.72]
Education	-5.36	2.36	-2.27	0.023	[-9.99, -0.73]
Current Student	-1.57	2.77	-0.57	0.571	[-7.01, 3.87]
Age	-2.61	1.32	-1.97	0.049	[-5.21, -0.02]
No Internet Issues	-4.11	2.31	-1.78	0.075	[-8.64, 0.42]
Time to Load Images	-3.60	0.96	-3.74	< 0.001	[-5.49, -1.71]
Subjective SES	-0.85	0.97	-0.88	0.378	[-2.75, 1.04]
Monetary Incentive × UK	38.75	4.00	9.69	< 0.001	[30.91, 46.59]
Study 2b: Mexico and the US (Same Nominal Pay)					
Monetary Incentive	29.25	3.14	9.32	< 0.001	[23.10, 35.41]
US	-38.98	2.92	-13.34	< 0.001	[-44.72, -33.25]
Female	-5.20	2.04	-2.55	0.011	[-9.20, -1.19]
Non-Binary	9.87	6.75	1.46	0.144	[-3.38, 23.11]
Education	-4.80	2.36	-2.03	0.042	[-9.42, -0.17]
Current Student	1.68	2.57	0.65	0.513	[-3.36, 6.72]
No Internet Issues	-5.37	2.51	-2.14	0.032	[-10.29, -0.46]
Time to Load Images	-0.94	1.03	-0.92	0.359	[-2.96, 1.07]
Lives in a City	-7.41	2.72	-2.73	0.006	[-12.74, -2.08]
Age	-1.57	1.28	-1.23	0.219	[-4.07, 0.93]
Subjective SES	-1.11	1.07	-1.03	0.302	[-3.21, 1.00]
Income: Not Employed	1.35	5.31	0.25	0.799	[-9.06, 11.75]
Income: Retired	-18.95	18.29	-1.04	0.300	[-54.82, 16.93]
Monetary Incentive × US	12.95	4.25	3.05	0.002	[4.62, 21.28]
Study 2b: Mexico and the US (Same Subjective Pay)					

	b	*SE*	*t*	*P*	95% CI
Monetary Incentive	28.96	3.18	9.12	< 0.001	[22.73, 35.20]
US	-37.35	2.92	-12.79	< 0.001	[-43.08, -31.62]
Female	-6.13	2.08	-2.95	0.003	[-10.20, -2.06]
Non-Binary	7.53	6.76	1.11	0.265	[-5.72, 20.77]
Education	-5.33	2.35	-2.27	0.023	[-9.94, -0.72]
Current Student	-1.32	2.53	-0.52	0.603	[-6.28, 3.65]
No Internet Issues	-8.07	2.53	-3.19	0.001	[-13.03, -3.11]
Time to Load Images	-1.87	1.00	-1.86	0.063	[-3.83, 0.10]
Lives in a City	-7.07	2.74	-2.58	0.010	[-12.44, -1.69]
Age	-3.07	1.26	-2.44	0.015	[-5.53, -0.60]
Subjective SES	1.43	1.08	1.32	0.187	[-0.69, 3.56]
Income: Not Employed	-3.91	5.64	-0.69	0.488	[-14.97, 7.15]
Income: Retired	-17.91	20.14	-0.89	0.374	[-57.41, 21.59]
Monetary Incentive × US	17.46	4.27	4.08	< 0.001	[9.07, 25.84]
Study 2c: South Africa and the US (Same Nominal Pay)					
Monetary Incentive	25.33	3.08	8.23	< 0.001	[19.29, 31.37]
US	-9.74	3.47	-2.81	0.005	[-16.54, -2.94]
Female	0.96	2.17	0.44	0.657	[-3.29, 5.22]
Non-Binary	-6.70	7.62	-0.88	0.380	[-21.65, 8.26]
Education	-6.76	2.34	-2.89	0.004	[-11.36, -2.16]
Current Student	-3.53	2.85	-1.24	0.216	[-9.11, 2.06]
No Internet Issues	-2.13	2.82	-0.75	0.452	[-7.66, 3.41]
Time to Load Images	-1.23	1.05	-1.17	0.243	[-3.28, 0.83]
Lives in a City	-1.92	2.40	-0.80	0.423	[-6.63, 2.78]
Age	-3.50	1.25	-2.80	0.005	[-5.95, -1.04]
Subjective SES	0.49	1.24	0.40	0.693	[-1.94, 2.92]
Income: $10,000 to $19,999	0.24	4.11	0.06	0.953	[-7.82, 8.31]
Income: $20,000 to $29,999	-1.22	4.14	-0.29	0.769	[-9.35, 6.91]
Income: $30,000 to $39,999	-0.32	4.64	-0.07	0.944	[-9.43, 8.78]
Income: $40,000 to $49,999	-1.69	4.85	-0.35	0.727	[-11.20, 7.82]

	b	*SE*	*t*	*P*	95% CI
Income: $50,000 to $59,999	-2.80	5.55	-0.51	0.614	[-13.70, 8.09]
Income: $60,000 to $69,999	0.54	5.64	0.10	0.924	[-10.52, 11.60]
Income: $70,000 to $79,999	-2.18	6.42	-0.34	0.734	[-14.77, 10.41]
Income: $80,000 to $89,999	3.65	7.55	0.48	0.629	[-11.17, 18.47]
Income: $90,000 to $99,999	5.97	6.43	0.93	0.354	[-6.66, 18.59]
Income: $100,000 to $149,999	-0.73	5.36	-0.14	0.891	[-11.25, 9.78]
Income: $150,000 or more	-7.67	6.04	-1.27	0.204	[-19.52, 4.17]
Income: Not Employed	-2.64	7.24	-0.36	0.715	[-16.85, 11.56]
Income: Student	-1.99	5.43	-0.37	0.715	[-12.65, 8.67]
Income: Retired	2.49	19.10	0.13	0.896	[-34.98, 39.96]
Monetary Incentive × US	8.44	4.25	1.98	0.047	[0.10, 16.78]
Study 2c: South Africa and the US (Same Subjective Pay)					
Monetary Incentive	24.51	3.12	7.87	< 0.001	[18.39, 30.62]
US	-9.77	3.50	-2.79	0.005	[-16.63, -2.90]
Female	-2.18	2.20	-0.99	0.322	[-6.49, 2.14]
Non-Binary	-13.31	8.19	-1.63	0.104	[-29.38, 2.75]
Education	-3.30	2.44	-1.36	0.176	[-8.09, 1.48]
Current Student	-4.69	3.01	-1.56	0.120	[-10.60, 1.22]
No Internet Issues	-7.40	2.78	-2.66	0.008	[-12.86, -1.95]
Time to Load Images	-1.78	1.06	-1.67	0.096	[-3.86, 0.31]
Lives in a City	-0.67	2.47	-0.27	0.786	[-5.53, 4.18]
Age	-1.15	1.26	-0.92	0.359	[-3.62, 1.32]
Subjective SES	0.46	1.25	0.36	0.716	[-2.00, 2.91]
Income: $10,000 to $19,999	-0.19	4.11	-0.05	0.964	[-8.25, 7.88]
Income: $20,000 to $29,999	-2.59	4.27	-0.60	0.545	[-10.97, 5.80]
Income: $30,000 to $39,999	-2.14	4.71	-0.45	0.650	[-11.38, 7.11]
Income: $40,000 to $49,999	3.14	4.92	0.64	0.524	[-6.51, 12.78]
Income: $50,000 to $59,999	-9.86	5.37	-1.84	0.067	[-20.39, 0.68]
Income: $60,000 to $69,999	-1.68	5.67	-0.30	0.768	[-12.79, 9.44]
Income: $70,000 to $79,999	-9.80	6.35	-1.54	0.123	[-22.27, 2.67]

	b	*SE*	*t*	*P*	95% CI
Income: $80,000 to $89,999	-7.71	7.31	-1.05	0.292	[-22.05, 6.63]
Income: $90,000 to $99,999	-7.92	7.29	-1.09	0.278	[-22.23, 6.39]
Income: $100,000 to $149,999	-6.69	5.30	-1.26	0.207	[-17.09, 3.71]
Income: $150,000 or more	-10.58	6.37	-1.66	0.097	[-23.08, 1.92]
Income: Not Employed	-1.72	7.67	-0.22	0.822	[-16.76, 13.32]
Income: Student	-0.61	5.28	-0.11	0.909	[-10.97, 9.76]
Income: Retired	-15.91	17.20	-0.93	0.355	[-49.66, 17.84]
Monetary Incentive × US	16.28	4.30	3.78	< 0.001	[7.84, 24.73]

Regression output for the linear regression models on the number of images rated in the samples from Studies 2a–c for each sample comparison pair, with additional controls added. Monetary Incentive is a binary variable for whether participants were in one of the monetary conditions. UK and US are binary variable for whether participants were part of the UK or one of the US samples, respectively. Female and Non-Binary are categorical variables for gender. Education is a binary variable specifying people who had either completed or were pursuing studies at the undergraduate level or higher at the time of the experiment. No Internet Issues is a binary variable for participants who reported having no problems with their Internet connection during the study. Time to Load Images is a scaled, continuous variable for self-reported average time to load each image. Live in Cities is a binary variable that specifies whether a participant lives in a medium- or large-sized city. Age is a continuous variable and is scaled. Subjective SES is a continuous variable for subjective socioeconomic status, ranging from 0 to 10. Income is a categorical variable, with “Income: Less than $10,000” coded as $5,000; “$10,000 to $19,999” coded as $15,000; “$20,000 to $29,999” coded as $25,000; “$30,000 to $39,999” coded as $35,000; “$40,000 to $49,999” coded as $45,000; “$50,000 to $59,999” coded as $55,000; “$60,000 to $69,999” coded as $65,000; “$70,000 to $79,999” coded as $75,000; “$80,000 to $89,999” coded as $85,000; “$90,000 to $99,999” coded as $95,000; “$100,000 to $149,999” coded as $125,000; and “$150,000 or more” coded as $175,000. Income also included non-numerical categories of “Not Employed,” “Student,” and “Retired.”

***Supplementary Table 13b:* Study 3a, Mean Effort in Norm Versus Minimal Pay Condition, with Additional Controls.**

	b	*SE*	*t*	*P*	95% CI
		Study 3a: India and the US			
Monetary Incentive	-0.54	4.41	-0.12	0.903	[-9.19, 8.11]
US	-27.50	5.92	-4.65	< 0.001	[-39.12, -15.88]
Female	1.42	3.29	0.43	0.667	[-5.05, 7.89]
Non-Binary	22.10	11.53	1.92	0.056	[-0.54, 44.73]
Education	-0.80	4.32	-0.18	0.853	[-9.27, 7.67]
Current Student	-3.93	6.16	-0.64	0.524	[-16.02, 8.16]
No Internet Issues	-8.67	3.76	-2.30	0.021	[-16.05, -1.28]
Time to Load Images	-1.33	1.49	-0.89	0.373	[-4.27, 1.60]
Lives in a City	-2.35	3.47	-0.68	0.499	[-9.16, 4.46]
Age	-0.57	1.67	-0.34	0.733	[-3.84, 2.71]
Subjective SES	-5.85	1.93	-3.04	0.002	[-9.63, -2.07]
Income: $10,000 to $19,999	-7.57	5.17	-1.46	0.144	[-17.73, 2.58]
Income: $20,000 to $29,999	-17.15	5.72	-3.00	0.003	[-28.39, -5.91]
Income: $30,000 to $39,999	-10.19	6.45	-1.58	0.115	[-22.86, 2.48]
Income: $40,000 to $49,999	-9.13	7.71	-1.18	0.237	[-24.27, 6.01]
Income: $50,000 to $59,999	-2.89	6.81	-0.43	0.671	[-16.27, 10.48]
Income: $60,000 to $69,999	-10.68	8.02	-1.33	0.183	[-26.43, 5.06]
Income: $70,000 to $79,999	4.34	7.95	0.55	0.585	[-11.27, 19.96]
Income: $80,000 to $89,999	-12.36	8.87	-1.39	0.164	[-29.76, 5.05]
Income: $90,000 to $99,999	-4.50	8.84	-0.51	0.611	[-21.86, 12.86]
Income: $100,000 to $149,999	4.76	8.16	0.58	0.560	[-11.26, 20.78]
Income: $150,000 or more	-4.71	9.70	-0.49	0.627	[-23.76, 14.33]
Income: Not Employed	-14.07	12.13	-1.16	0.246	[-37.89, 9.74]
Income: Student	-6.62	16.70	-0.40	0.692	[-39.41, 26.16]
Income: Retired	23.44	28.72	0.82	0.415	[-32.95, 79.82]
Monetary Incentive × US	14.61	6.10	2.39	0.017	[2.63, 26.59]

Regression output for the linear regression models on the number of images rated in Study 3a, with additional controls added. Monetary Incentive is a binary variable for whether participants were assigned to the monetary (minimal pay) condition. US is a binary variable for whether participants were part of the US sample. Female and Non-Binary are categorical variables for gender. Education is a binary variable specifying people who had either completed or were pursuing studies at the undergraduate level or higher at the time of the experiment. Current Student is a binary variable for those who report being currently enrolled as students. Lives in a City is a binary variable for participants who reported living in a medium-sized or large city. No Internet Issues is a binary variable for participants who reported having no problems with their Internet connection during the study. Time to Load Images a continuous variable for self-reported time it took for each image to load. Age is a continuous variable and is scaled. Subjective SES is a continuous variable for subjective socioeconomic status, ranging from 0 to 10. Income is a categorical variable, with "Income: Less than $10,000" coded as $5,000; "$10,000 to $19,999" coded as $15,000; "$20,000 to $29,999" coded as $25,000; "$30,000 to $39,999" coded as $35,000; "$40,000 to $49,999" coded as $45,000; "$50,000 to $59,999" coded as $55,000; "$60,000 to $69,999" coded as $65,000; "$70,000 to $79,999" coded as $75,000; "$80,000 to $89,999" coded as $85,000; "$90,000 to $99,999" coded as $95,000; "$100,000 to $149,999" coded as $125,000; and "$150,000 or more" coded as $175,000. Income also included non-numerical categories of "Not Employed," "Student," and "Retired."

ADDITIONAL ANALYSES FOR STUDY 3A

As specified in the Methods section for Study 3a, we pre-registered t-tests to compare the two incentive conditions within each country, with a prediction that the two incentives would be statistically indistinguishable in India but not in the US.

In the US, participants completed more images in the minimal pay compared to the norm condition, two-sided Welch's $t(354.7) = 3.45$, $P < 0.001$, $Mean_{\text{difference}} = 14.91$, 95% CI 6.41 to 23.41. In India, there was no statistically significant difference, two-sided Welch's $t(349.02) = 0.21$, $P = 0.834$, $Mean_{\text{difference}} = 0.84$, 95% CI -7.04 to 8.71.

The pre-registered analysis also specified recruiting 352 participants per country post-exclusions. Since fewer people than we had thought did not pass the checks in the US, we ended up with a final sample of 382 participants. Below we repeat the analysis reported in the paper, including only the first 352 participants in the US.

In the US, the *t*-test for differences between the two incentive conditions on the number of images rated remains statistically significant, two-sided Welch's $t(316.74) = 3.89$, $P < 0.001$, $Mean_{\text{difference}} = 17.17$, 95% CI 8.48 to 25.87.

The interaction between culture and incentive, reported in the main test as $b = 13.77$, $t(726) = 2.31$, $P = 0.021$, 95% CI 2.09 to 25.45, becomes $b = 16.11$, $t(696) = 2.68$, $P = 0.007$, 95% CI 4.32 to 27.89.

EXPLORATORY VARIABLES

In the samples that we recruited relatively early in the project (Studies 2a–b and Study 4), we included 7 exploratory variables that asked participants to report their motivation for completing the task and satisfaction with their performance, all on a 7-point Likert scale (1 = "Strongly Disagree" to 7 = "Strongly Agree"). We did not analyze these variables until after we had made the (wrong) decision to exclude them from future studies to minimize the time it takes to complete the survey. We report these variables for samples in Studies 2a, 2b, and 4 in Supplementary Tables 14, 15a, 15b, and 16. Results for Study 4 are also presented in Fig. S3.

Additional Variables Across Studies

In the earlier studies we ran, we collected more items measured on the same scale as the 7 exploratory DVs. For instance, we gave participants the relational mobility scale [9]. We did this to adjust for different cultural response styles by ipsatizing [10] the 7 exploratory variables. Ipsatization is a procedure used in cultural and personality psychology that adjusts people's responses to remove the influence of cultural differences in response styles. Ipsatization removes the mean of the items measured on the same scale but measuring different constructs from each variable of interest. We only analyzed the exploratory variables in the main text in Study 4, where no adjustments for different cultural response styles were needed (because participants came from the same culture—India). Thus, we did not use any of these additional measures. However, they are available in the study materials from Qualtrics.

Individual Differences in Study 4

In Study 4, we first collected data from numerous participants in India on a number of cultural scales (tightness/looseness [11,12], relational mobility [9], self-construal [13]) for a different

project. A week later, some of these participants completed the task for Study 4. We considered studying how individual differences might affect how many images people rate in the monetary and norm conditions in India. However, we ultimately decided against the idea of including the discussion of individual differences and did not analyze these data for this project.

***Supplementary Table 14:* Exploratory Variables in Study 2a.**

	b	*SE*	*t*	*P*	P_{Bonf}	99.3% CI
I enjoyed completing the task						
(Intercept)	5.88	0.07	78.79	< 0.001	< 0.001	[5.68, 6.09]
Monetary Incentive	0.07	0.08	0.92	0.359	1.000	[-0.14, 0.29]
UK	-0.60	0.08	-7.68	< 0.001	< 0.001	[-0.81, -0.39]
Female	0.04	0.05	0.82	0.415	1.000	[-0.10, 0.19]
Non-Binary	-0.11	0.36	-0.31	0.753	1.000	[-1.08, 0.85]
Education	-0.31	0.06	-4.85	< 0.001	< 0.001	[-0.48, -0.14]
Age	0.17	0.03	5.00	< 0.001	< 0.001	[0.08, 0.27]
Monetary Incentive × UK	0.17	0.11	1.50	0.134	0.941	[-0.14, 0.47]
I am satisfied with how well I did on the task						
(Intercept)	5.93	0.07	90.73	< 0.001	< 0.001	[5.75, 6.10]
Monetary Incentive	-0.01	0.07	-0.13	0.900	1.000	[-0.20, 0.18]
UK	0.10	0.07	1.41	0.158	1.000	[-0.09, 0.28]
Female	-0.12	0.05	-2.57	0.010	0.071	[-0.25, 0.01]
Non-Binary	0.14	0.31	0.44	0.659	1.000	[-0.71, 0.98]
Education	-0.07	0.06	-1.28	0.201	1.000	[-0.22, 0.08]
Age	0.08	0.03	2.65	0.008	0.056	[0.00, 0.16]
Monetary Incentive × UK	0.16	0.10	1.60	0.111	0.776	[-0.11, 0.42]
I believe that I helped others by completing the task						
(Intercept)	6.12	0.07	82.09	< 0.001	< 0.001	[5.92, 6.32]
Monetary Incentive	-0.05	0.08	-0.58	0.565	1.000	[-0.26, 0.17]
UK	-1.08	0.08	-13.84	< 0.001	< 0.001	[-1.29, -0.87]
Female	-0.03	0.05	-0.53	0.594	1.000	[-0.17, 0.12]
Non-Binary	-0.34	0.36	-0.95	0.342	1.000	[-1.30, 0.63]
Education	-0.15	0.06	-2.43	0.015	0.105	[-0.32, 0.02]
Age	0.08	0.03	2.29	0.022	0.156	[-0.01, 0.17]
Monetary Incentive × UK	0.23	0.11	2.02	0.044	0.306	[-0.08, 0.53]
Completing the task was boring						

	b	SE	t	P	P_{Bonf}	99.3% CI
(Intercept)	2.95	0.09	31.56	< 0.001	< 0.001	[2.7, 3.2]
Monetary Incentive	-0.06	0.10	-0.60	0.552	1.000	[-0.33, 0.21]
UK	0.42	0.10	4.33	< 0.001	< 0.001	[0.16, 0.69]
Female	-0.12	0.07	-1.77	0.077	0.540	[-0.30, 0.06]
Non-Binary	-0.26	0.45	-0.57	0.568	1.000	[-1.47, 0.95]
Education	0.20	0.08	2.57	0.010	0.071	[-0.01, 0.42]
Age	-0.21	0.04	-4.82	< 0.001	< 0.001	[-0.33, -0.09]
Monetary Incentive × UK	0.01	0.14	0.06	0.951	1.000	[-0.37, 0.39]
I only completed the task for money						
(Intercept)	3.36	0.10	33.82	< 0.001	< 0.001	[3.09, 3.63]
Monetary Incentive	0.06	0.11	0.61	0.541	1.000	[-0.22, 0.35]
UK	1.30	0.10	12.56	< 0.001	< 0.001	[1.02, 1.58]
Female	-0.01	0.07	-0.12	0.907	1.000	[-0.20, 0.18]
Non-Binary	0.24	0.48	0.51	0.610	1.000	[-1.04, 1.53]
Education	0.11	0.08	1.34	0.181	1.000	[-0.11, 0.34]
Age	-0.20	0.05	-4.23	< 0.001	< 0.001	[-0.32, -0.07]
Monetary Incentive × UK	-0.04	0.15	-0.28	0.778	1.000	[-0.45, 0.36]
I could have assessed more pictures if I’d tried harder						
(Intercept)	5.92	0.09	65.71	< 0.001	< 0.001	[5.68, 6.17]
Monetary Incentive	0.09	0.10	0.93	0.352	1.000	[-0.17, 0.35]
UK	-1.09	0.09	-11.60	< 0.001	< 0.001	[-1.35, -0.84]
Female	-0.13	0.06	-1.95	0.052	0.362	[-0.30, 0.05]
Non-Binary	0.41	0.43	0.94	0.348	1.000	[-0.76, 1.57]
Education	-0.13	0.08	-1.76	0.079	0.555	[-0.34, 0.07]
Age	0.05	0.04	1.17	0.241	1.000	[-0.06, 0.16]
Monetary Incentive × UK	-0.63	0.14	-4.62	< 0.001	< 0.001	[-0.99, -0.26]
I am satisfied with the amount of pay I received						
(Intercept)	6.03	0.06	93.73	< 0.001	< 0.001	[5.86, 6.21]
Monetary Incentive	0.01	0.07	0.07	0.941	1.000	[-0.18, 0.19]
UK	-0.35	0.07	-5.19	< 0.001	< 0.001	[-0.53, -0.17]

	b	SE	t	P	P_{Bonf}	99.3% CI
Female	0.01	0.05	0.31	0.753	1.000	[-0.11, 0.14]
Non-Binary	-0.41	0.31	-1.34	0.181	1.000	[-1.25, 0.42]
Education	-0.18	0.05	-3.24	0.001	0.008	[-0.32, -0.03]
Age	0.05	0.03	1.51	0.131	0.919	[-0.04, 0.13]
Monetary Incentive × UK	-0.09	0.10	-0.91	0.364	1.000	[-0.35, 0.17]

Results of linear regression models on the seven exploratory variables that participants completed after the image-rating task in Study 2a. Monetary Incentive is a binary variable for whether participants were in one of the monetary conditions. UK is a binary variable for whether participants were part of the UK sample. Female and Non-Binary are categorical variables for gender. Education is a binary variable specifying people who had either completed or were pursuing studies at the undergraduate level or higher at the time of the experiment. Age is a continuous variable and is scaled. P_{Bonf} represents p-values after Bonferroni corrections for multiple comparisons.

Supplementary Table 15a: **Exploratory Variables in Study 2b (Mexico and US Sample with Same Nominal Pay).**

	b	*SE*	*t*	*P*	P_{Bonf}	99.3% CI
			I enjoyed completing the task			
(Intercept)	5.56	0.08	74.10	< 0.001	< 0.001	[5.36, 5.76]
Monetary Incentive	0.25	0.08	2.96	0.003	0.022	[0.02, 0.47]
US	-0.47	0.07	-6.28	< 0.001	< 0.001	[-0.67, -0.27]
Female	0.26	0.06	4.61	< 0.001	< 0.001	[0.11, 0.41]
Non-Binary	-0.34	0.18	-1.87	0.062	0.434	[-0.84, 0.15]
Education	-0.25	0.06	-4.11	< 0.001	< 0.001	[-0.41, -0.09]
Age	0.15	0.03	4.73	< 0.001	< 0.001	[0.06, 0.24]
Monetary Incentive × US	-0.16	0.12	-1.40	0.163	1.000	[-0.47, 0.15]
			I am satisfied with how well I did on the task			
(Intercept)	6.31	0.05	116.09	< 0.001	< 0.001	[6.16, 6.45]
Monetary Incentive	0.08	0.06	1.32	0.186	1.000	[-0.08, 0.24]
US	-0.31	0.05	-5.70	< .001	< 0.001	[-0.45, -0.16]
Female	0.03	0.04	0.86	0.392	1.000	[-0.07, 0.14]
Non-Binary	-0.05	0.13	-0.36	0.720	1.000	[-0.41, 0.31]
Education	-0.01	0.04	-0.33	0.738	1.000	[-0.13, 0.10]
Age	0.06	0.02	2.64	0.008	0.059	[-0.00, 0.12]
Monetary Incentive × US	-0.11	0.08	-1.27	0.206	1.000	[-0.33, 0.12]
			I believe that I helped others by completing the task			
(Intercept)	5.67	0.08	70.56	< 0.001	< 0.001	[5.46, 5.89]
Monetary Incentive	0.04	0.09	0.46	0.645	1.000	[-0.20, 0.28]
US	-0.74	0.08	-9.28	< 0.001	< 0.001	[-0.96, -0.53]
Female	0.07	0.06	1.18	0.238	1.000	[-0.09, 0.23]
Non-Binary	-0.34	0.20	-1.75	0.080	0.560	[-0.87, 0.19]
Education	-0.06	0.07	-0.93	0.353	1.000	[-0.24, 0.12]
Age	-0.02	0.03	-0.48	0.629	1.000	[-0.11, 0.08]
Monetary Incentive × US	-0.12	0.12	-0.95	0.342	1.000	[-0.45, 0.22]
			Completing the task was boring			

	b	SE	t	P	P_{Bonf}	99.3% CI
(Intercept)	2.35	0.09	26.79	< 0.001	< 0.001	[2.11, 2.59]
Monetary Incentive	-0.12	0.10	-1.24	0.215	1.000	[-0.38, 0.14]
US	1.23	0.09	14.11	< 0.001	< 0.001	[0.99, 1.46]
Female	-0.29	0.07	-4.41	< 0.001	< 0.001	[-0.46, -0.11]
Non-Binary	0.27	0.21	1.27	0.204	1.000	[-0.31, 0.85]
Education	0.32	0.07	4.47	< 0.001	< 0.001	[0.13, 0.51]
Age	-0.28	0.04	-7.40	< 0.001	< 0.001	[-0.38, -0.18]
Monetary Incentive × US	0.08	0.14	0.61	0.540	1.000	[-0.28, 0.45]
I only completed the task for money						
(Intercept)	3.93	0.09	42.92	< 0.001	< 0.001	[3.68, 4.17]
Monetary Incentive	0.37	0.10	3.67	< 0.001	0.002	[0.10, 0.64]
US	1.25	0.09	13.79	< 0.001	< 0.001	[1.01, 1.50]
Female	-0.34	0.07	-4.95	< 0.001	< 0.001	[-0.52, -0.15]
Non-Binary	-0.37	0.22	-1.63	0.103	0.724	[-0.97, 0.24]
Education	0.14	0.07	1.95	0.051	0.360	[-0.06, 0.34]
Age	-0.19	0.04	-4.92	< 0.001	< 0.001	[-0.30, -0.09]
Monetary Incentive × US	-0.21	0.14	-1.50	0.135	0.942	[-0.59, 0.17]
I could have assessed more pictures if I'd tried harder						
(Intercept)	4.87	0.10	49.34	< 0.001	< 0.001	[4.61, 5.14]
Monetary Incentive	-0.07	0.11	-0.60	0.548	1.000	[-0.36, 0.23]
US	0.08	0.10	0.85	0.397	1.000	[-0.18, 0.35]
Female	-0.18	0.07	-2.39	0.017	0.118	[-0.37, 0.02]
Non-Binary	-0.79	0.24	-3.27	0.001	0.008	[-1.44, -0.14]
Education	0.10	0.08	1.24	0.217	1.000	[-0.12, 0.31]
Age	0.03	0.04	0.65	0.516	1.000	[-0.09, 0.14]
Monetary Incentive × US	-0.20	0.15	-1.30	0.194	1.000	[-0.61, 0.21]
I am satisfied with the amount of pay I received						
(Intercept)	5.69	0.06	88.92	< 0.001	< 0.001	[5.52, 5.86]
Monetary Incentive	0.13	0.07	1.79	0.074	0.518	[-0.06, 0.32]
US	0.23	0.06	3.55	< 0.001	0.003	[0.05, 0.40]

	b	SE	t	P	P_{Bonf}	99.3% CI
Female	0.02	0.05	0.43	0.670	1.000	[-0.11, 0.15]
Non-Binary	-0.42	0.16	-2.67	0.008	0.053	[-0.84, 0.00]
Education	-0.01	0.05	-0.28	0.779	1.000	[-0.15, 0.13]
Age	0.02	0.03	0.78	0.437	1.000	[-0.05, 0.09]
Monetary Incentive × US	-0.44	0.10	-4.50	< 0.001	< 0.001	[-0.71, -0.18]

Results of linear regression models on the seven exploratory variables that participants completed after the image-rating task in Study 2b for the comparison between samples in Mexico and the US (same nominal pay as in Mexico). Monetary Incentive is a binary variable for participants assigned to the monetary condition. US is a binary variable for participants in the sample from the US. Female and Non-Binary are categorical variables for gender. Education is a binary variable specifying people who had either completed or were pursuing studies at the undergraduate level or higher at the time of the experiment. Age is a continuous variable and is scaled. P_{Bonf} represents p-values after Bonferroni corrections for multiple comparisons.

Supplementary Table 15b: **Exploratory Variables in Study 2b (Mexico and US Sample with the Same Subjective Pay).**

	b	*SE*	*t*	*P*	P_{Bonf}	99.3% CI
I enjoyed completing the task						
(Intercept)	5.47	0.07	75.00	< 0.001	< 0.001	[5.28, 5.67]
Monetary Incentive	0.25	0.08	3.01	0.003	0.018	[0.03, 0.47]
US	-0.40	0.07	-5.58	< 0.001	< 0.001	[-0.60, -0.21]
Female	0.30	0.06	5.45	< 0.001	< 0.001	[0.15, 0.45]
Non-Binary	-0.12	0.18	-0.68	0.499	1.000	[-0.61, 0.36]
Education	-0.18	0.06	-3.08	0.002	0.015	[-0.34, -0.02]
Age	0.13	0.03	4.24	< 0.001	< 0.001	[0.05, 0.21]
Monetary Incentive × US	0.01	0.11	0.07	0.947	1.000	[-0.30, 0.31]
I am satisfied with how well I did on the task						
(Intercept)	6.24	0.05	116.35	< 0.001	< 0.001	[6.10, 6.39]
Monetary Incentive	0.08	0.06	1.34	0.182	1.000	[-0.08, 0.24]
US	-0.27	0.05	-5.04	< 0.001	< 0.001	[-0.41, -0.12]
Female	0.05	0.04	1.15	0.250	1.000	[-0.06, 0.16]
Non-Binary	0.03	0.13	0.25	0.804	1.000	[-0.32, 0.39]
Education	0.02	0.04	0.49	0.626	1.000	[-0.10, 0.14]
Age	0.02	0.02	0.86	0.389	1.000	[-0.04, 0.08]
Monetary Incentive × US	-0.01	0.08	-0.15	0.878	1.000	[-0.24, 0.21]
I believe that I helped others by completing the task						
(Intercept)	5.66	0.08	74.87	< 0.001	< 0.001	[5.46, 5.86]
Monetary Incentive	0.04	0.08	0.49	0.621	1.000	[-0.19, 0.27]
US	-0.67	0.07	-8.88	< 0.001	< 0.001	[-0.87, -0.46]
Female	0.09	0.06	1.52	0.128	0.895	[-0.07, 0.24]
Non-Binary	-0.26	0.19	-1.41	0.158	1.000	[-0.76, 0.24]
Education	-0.08	0.06	-1.33	0.183	1.000	[-0.25, 0.08]
Age	-0.04	0.03	-1.33	0.184	1.000	[-0.13, 0.04]
Monetary Incentive × US	0.03	0.12	0.30	0.766	1.000	[-0.28, 0.35]
Completing the task was boring						

	b	SE	t	P	P_{Bonf}	99.3% CI
(Intercept)	2.39	0.09	27.94	< 0.001	< 0.001	[2.16, 2.62]
Monetary Incentive	-0.12	0.10	-1.23	0.219	1.000	[-0.38, 0.14]
US	1.05	0.08	12.35	< 0.001	< 0.001	[0.82, 1.28]
Female	-0.27	0.06	-4.14	< 0.001	< 0.001	[-0.44, -0.09]
Non-Binary	0.02	0.21	0.09	0.931	1.000	[-0.55, 0.59]
Education	0.32	0.07	4.57	< 0.001	< 0.001	[0.13, 0.51]
Age	-0.20	0.04	-5.44	< 0.001	< 0.001	[-0.30, -0.10]
Monetary Incentive × US	-0.06	0.13	-0.45	0.653	1.000	[-0.42, 0.30]
I only completed the task for money						
(Intercept)	3.97	0.09	42.84	< 0.001	< 0.001	[3.72, 4.22]
Monetary Incentive	0.37	0.10	3.60	< 0.001	0.002	[0.09, 0.65]
US	1.12	0.09	12.20	< 0.001	< 0.001	[0.87, 1.37]
Female	-0.33	0.07	-4.72	< 0.001	< 0.001	[-0.52, -0.14]
Non-Binary	-0.62	0.23	-2.71	0.007	0.047	[-1.23, -0.00]
Education	0.14	0.08	1.85	0.065	0.455	[-0.06, 0.34]
Age	-0.13	0.04	-3.36	0.001	0.006	[-0.24, -0.03]
Monetary Incentive × US	-0.48	0.14	-3.33	0.001	0.006	[-0.87, -0.09]
I could have assessed more pictures if I'd tried harder						
(Intercept)	4.84	0.10	49.63	< 0.001	< 0.001	[4.58, 5.11]
Monetary Incentive	-0.06	0.11	-0.55	0.584	1.000	[-0.35, 0.23]
US	0.08	0.10	0.82	0.412	1.000	[-0.18, 0.34]
Female	-0.08	0.07	-1.03	0.305	1.000	[-0.27, 0.12]
Non-Binary	-0.78	0.24	-3.26	< 0.001	0.008	[-1.43, -0.14]
Education	0.11	0.08	1.32	0.186	1.000	[-0.11, 0.32]
Age	0.07	0.04	1.69	0.090	0.632	[-0.04, 0.18]
Monetary Incentive × US	-0.40	0.15	-2.65	0.008	0.056	[-0.81, 0.01]
I am satisfied with the amount of pay I received						
(Intercept)	5.70	0.06	91.98	< 0.001	< 0.001	[5.53, 5.86]
Monetary Incentive	0.13	0.07	1.86	0.064	0.446	[-0.06, 0.32]
US	0.27	0.06	4.37	< 0.001	< 0.001	[0.10, 0.43]

	b	*SE*	*t*	*P*	P_{Bonf}	99.3% CI
Female	0.10	0.05	2.07	0.039	0.272	[-0.03, 0.22]
Non-Binary	-0.16	0.15	-1.08	0.282	1.000	[-0.58, 0.25]
Education	-0.08	0.05	-1.60	0.111	0.774	[-0.22, 0.06]
Age	0.02	0.03	0.82	0.411	1.000	[-0.05, 0.09]
Monetary Incentive × US	-0.43	0.10	-4.47	< 0.001	< 0.001	[-0.69, -0.17]

Results of linear regression models on the seven exploratory variables that participants completed after the image-rating task in Study 2b for the comparison between samples in Mexico and the US (same subjective pay as in Mexico). Monetary Incentive is a binary variable for participants assigned to the monetary condition. US is a binary variable for participants in the sample from the US. Female and Non-Binary are categorical variables for gender. Education is a binary variable specifying people who had either completed or were pursuing studies at the undergraduate level or higher at the time of the experiment. Age is a continuous variable and is scaled. P_{Bonf} represents *p*-values after Bonferroni corrections for multiple comparisons.

Supplementary Table 16: **Exploratory Variables in Study 4.**

	b	*SE*	*t*	*P*	P_{Bonf}	99.3% CI
			I enjoyed completing the task			
(Intercept)	5.85	0.06	98.02	< 0.001	< 0.001	[5.69, 6.01]
Monetary Incentive	0.29	0.08	3.41	0.001	0.005	[0.06, 0.51]
English	0.21	0.08	2.44	0.015	0.104	[-0.02, 0.43]
Monetary Incentive × English	-0.17	0.12	-1.38	0.167	1.000	[-0.49, 0.16]
			I am satisfied with how well I did on the task			
(Intercept)	6.14	0.05	122.27	< 0.001	< 0.001	[6.00, 6.27]
Monetary Incentive	0.13	0.07	1.89	0.059	0.410	[-0.06, 0.33]
English	-0.01	0.07	-0.21	0.833	1.000	[-0.21, 0.18]
Monetary Incentive × English	-0.20	0.10	-1.98	0.048	0.337	[-0.47, 0.07]
			I believe that I helped others by completing the task			
(Intercept)	5.70	0.06	91.22	< 0.001	< 0.001	[5.53, 5.87]
Monetary Incentive	0.12	0.09	1.34	0.180	1.000	[-0.12, 0.36]
English	0.14	0.09	1.57	0.117	0.822	[-0.10, 0.38]
Monetary Incentive × English	-0.15	0.13	-1.21	0.226	1.000	[-0.49, 0.19]
			Completing the task was boring			
(Intercept)	2.69	0.07	36.23	< 0.001	< 0.001	[2.49, 2.89]
Monetary Incentive	-0.17	0.11	-1.64	0.100	0.703	[-0.46, 0.11]
English	0.15	0.11	1.41	0.160	1.000	[-0.14, 0.43]
Monetary Incentive × English	0.13	0.15	0.84	0.402	1.000	[-0.28, 0.53]
			I only completed the task for money			
(Intercept)	3.22	0.08	39.62	< .001	< 0.001	[3.00, 3.44]
Monetary Incentive	0.02	0.11	0.21	0.831	1.000	[-0.29, 0.33]
English	0.40	0.12	3.49	0.001	0.004	[0.09, 0.71]

	b	SE	t	P	P_{Bonf}	99.3% CI
Monetary Incentive × English	0.21	0.16	1.30	0.195	1.000	[-0.23, 0.65]
I could have assessed more pictures if I'd tried harder						
(Intercept)	4.97	0.07	67.40	< 0.001	< 0.001	[4.77, 5.17]
Monetary Incentive	0.13	0.10	1.30	0.195	1.000	[-0.15, 0.42]
English	0.22	0.10	2.11	0.035	0.242	[-0.06, 0.50]
Monetary Incentive × English	0.07	0.15	0.50	0.617	1.000	[-0.33, 0.47]
I am satisfied with the amount of pay I received						
(Intercept)	5.68	0.06	99.36	< 0.001	< 0.001	[5.53, 5.83]
Monetary Incentive	0.03	0.08	0.43	0.670	1.000	[-0.18, 0.25]
English	0.00	0.08	0.01	0.980	1.000	[-0.22, 0.22]
Monetary Incentive × English	0.00	0.11	-0.03	0.980	1.000	[-0.31, 0.31]

Results of linear regression models on the seven exploratory variables that participants completed after the image-rating task in Study 4. Monetary Incentive is a binary variable for being assigned to the monetary condition. English is a binary variable for those who took the survey in English (as opposed to in Hindi). P_{Bonf} represents p-values after Bonferroni corrections for multiple comparisons.

Fig. S3: **Exploratory Variables in Study 4, by Language and Incentive Type.**

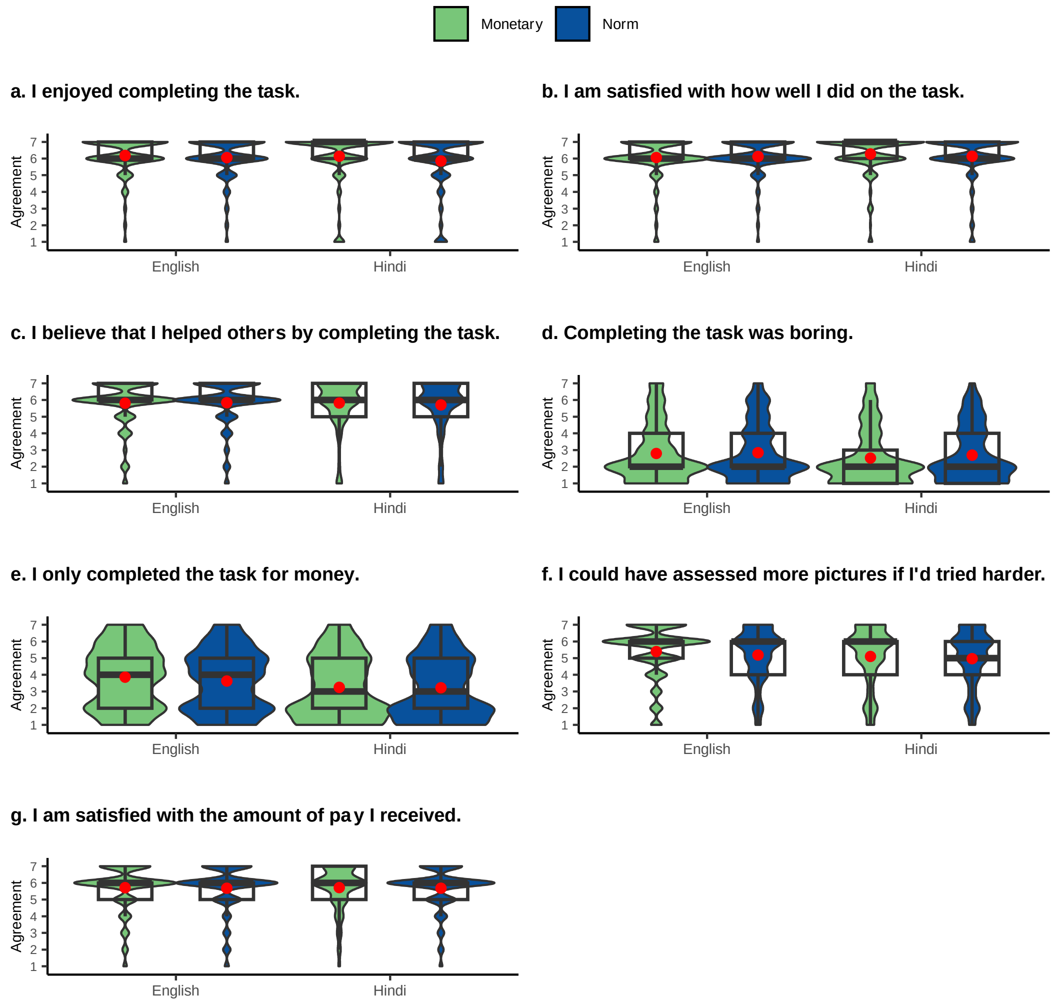

Effects of the monetary condition (green) and the norm condition (blue), by assigned language (English or Hindi), on the seven exploratory variables that participants in India (*N* = 2,065 participants recruited on Facebook) completed after the image-rating task in Study 4. The graph displays the central tendency and distribution of effort by incentive condition. The black line within each box represents a median, and the red dot shows a mean. Upper and lower bounds show the third and the first quartile, respectively. The whiskers represent the 1.5 times the interquartile range, with black points showing observations outside of this range. The width of each violin corresponds to the frequency of observations at any given number of images rated on the y-axis. Supplementary Table 16 reports the results of the statistical tests.

ADDITIONAL DISCUSSION

Conceptualizing the Task As "Work" Versus "Non-Work"

A possibility complementary to our explanation based on exchange norms and psychological contracts comes from previous research that shows that the same activity can be framed as "work" or as "non-work" (e.g., "leisure") [14,15]. Categorizing an activity as "work" has been found to shift the motivational focus to more extrinsic factors, such as money [16–18]. It is possible that WEIRD (as compared to non-WEIRD) participants in our studies were more likely to frame the image-rating task as "work," which could have made them more likely to adopt the rules of market exchange and thus prioritize monetary rewards. If this is the case, our findings are particularly counterintuitive considering that non-WEIRD workers on crowdsourcing platforms report that earning money is a more important motivator for completing tasks on these platforms and that they rely on crowdsourcing income more than their WEIRD peers [19–21].

Is The Effect Driven by Money or by Psychological Interventions?

Does the cultural variation in the money advantage show that money is more effective in WEIRD cultures, psychological interventions are more effective in non-WEIRD cultures, or both?

First, the answer seems to depend on each individual pair of countries. For example, in South Africa and the US, participants seemed equally incentivized by money, and the effect seemed to come from non-monetary incentives being more effective in South Africa.

On the other hand, participants in China did not seem to be more motivated than people in the UK by psychological interventions. Instead, money being more motivating in the UK than in China seemed to drive the interaction.

Similarly, in Study 4, money seemed to be driving the effect of the language frames on effort. Post-hoc tests (with Bonferroni corrections) found that money was significantly more motivating in English than in Hindi, two-sided Welch's $t(1030.41) = 3.94$, $P < 0.001$, $P_{Bonf} < 0.001$, $Mean_{\text{difference}} = 8.94$, 95% CI 4.48 to 13.40, 97.5% CI 3.84 to 14.04, $d = 0.24$. However, language did not significantly change how motivating the social norm was, two-sided Welch's $t(1028.98) = -0.760$, $P = 0.447$, $P_{Bonf} = 0.894$, $Mean_{\text{difference}} = -1.74$, $d = -0.05$, 95% CI -6.25 to 2.76, 97.5% CI -6.90 to 3.41.

Second, we think it is hard to make absolute claims about the motivating force of money. Consider the finding by Gneezy and colleagues who showed that monetary incentives improved students' performance on a math test in the US but not in China [22]. Do their results show that in the US money is more motivating? In both countries, the study had to compare the effectiveness of money to some baseline level of motivation that was already there (such as recognition from parents). Perhaps students in China were more motivated to perform well without money, and so

adding money to the mix of motivators could only raise their effort a little. This makes it difficult to conclude that money—and nothing else—is what produced the cultural difference.

In our studies, the “control” condition similarly cannot be a true blank slate. People in different cultures responded differently to the base-pay condition, and these differences represent meaningful cultural differences in motivation and mental frames. Fortunately, this is also practical. When deciding whether to implement a monetary incentive, what managers and policy-makers usually want to assess is whether it will increase effort compared to other less costly interventions.

REFERENCES FOR SUPPLEMENTARY MATERIALS

1. Becker, G. M., DeGroot, M. H. & Marschak, J. Measuring utility by a single-response sequential method. *Behavioral Science* **9**, 226–232 (1964).
2. DellaVigna, S. & Pope, D. What motivates effort? Evidence and expert forecasts. *The Review of Economic Studies* **85**, 1029–1069 (2018).
3. Jose and Roxanne. Flowers. Flickr. 2010. https://www.flickr.com/photos/nyc_xmas/4715530598/ (accessed October 21, 2023).
4. MasterPhoto-DK. Building 1. Flickr. 2007. https://www.flickr.com/photos/euromagic/436810411/ (accessed October 21, 2023).
5. Morschheuser, B. & Hamari, J. The gamification of work: Lessons from crowdsourcing. *Journal of Management Inquiry* **28**, 145–148 (2019).
6. OECD. Purchasing power parities (PPP): Total, National currency units/US dollar, 2000 – 2022. (2022) doi:10.1787/1290ee5a-en.
7. Bandiera, O., Dahlstrand-Rudin, A. & Fischer, G. Incentives and Culture: Evidence from a Multi-Country Field Experiment. *Working Paper* (2020) doi:https://doi.org/10.1257/rct.4685-2.1.
8. World Bank. Adjusted net national income per capita (current US$). https://data.worldbank.org/indicator/NY.ADJ.NNTY.PC.CD (2020).
9. Thomson, R. *et al.* Relational mobility predicts social behaviors in 39 countries and is tied to historical farming and threat. *Proceedings of the National Academy of Sciences* **115**, 7521–7526 (2018).
10. Bartram, D. The relationship between ipsatized and normative measures of personality. *Journal of Occupational and Organizational Psychology* **69**, 25–39 (1996).

11. Jackson, J. C., Gelfand, M. & Ember, C. R. A global analysis of cultural tightness in non-industrial societies. *Proceedings of the Royal Society B* **287**, 20201036 (2020).

12. Gelfand, M. J. *et al.* Differences between tight and loose cultures: A 33-nation study. *Science* **332**, 1100–1104 (2011).

13. Singelis, T. M. The Measurement of Independent and Interdependent Self-Construals. *Personality and Social Psychology Bulletin* **20**, 580–591 (1994).

14. Etkin, J. & Memmi, S. A. Goal conflict encourages work and discourages leisure. *Journal of Consumer Research* **47**, 716–736 (2021).

15. Fishbach, A. & Choi, J. When thinking about goals undermines goal pursuit. *Organizational Behavior and Human Decision Processes* **118**, 99–107 (2012).

16. Laran, J. & Janiszewski, C. Work or fun? How task construal and completion influence regulatory behavior. *Journal of Consumer Research* **37**, 967–983 (2011).

17. Tang, T. L. & Baumeister, R. F. Effects of personal values, perceived surveillance, and task labels on task preference: The ideology of turning play into work. *Journal of Applied Psychology* **69**, 99–105 (1984).

18. Glynn, M. A. Effects of work task cues and play task cues on information processing, judgment, and motivation. *Journal of Applied Psychology* **79**, 34–45 (1994).

19. Rogstadius, J. *et al.* An assessment of intrinsic and extrinsic motivation on task performance in crowdsourcing markets. in *Proceedings of the International AAAI Conference on Web and Social Media* vol. 5 321–328 (2011).

20. Ipeirotis, P. G. Demographics of Mechanical Turk. *Microeconomics: Search; Learning; Information Costs & Specific Knowledge; Expectation & Speculation eJournal* (2010).

21. Jiang, L., Wagner, C. & Nardi, B. Not just in it for the money: a qualitative investigation of workers' perceived benefits of micro-task crowdsourcing. in *2015 48th Hawaii International Conference on System Sciences* 773–782 (IEEE, 2015). doi:10.1109/HICSS.2015.98.

22. Gneezy, U. *et al.* Measuring success in education: the role of effort on the test itself. *American Economic Review: Insights* **1**, 291–308 (2019).
