# Supplementary material for: The motivating effect of monetary over psychological incentives is stronger in WEIRD cultures
Source: Nat Hum Behav. 2024 Jan 8;8(3):456–70. doi: 10.1038/s41562-023-01769-5 (PMC10963269; doi:10.1038/s41562-023-01769-5)
Supplement: Supplementary file 2 — Reporting Summary [file 41562_2023_1769_MOESM2_ESM.pdf]

nature portfolio

Corresponding author(s): Danila Medvedev

Last updated by author(s): 10/21/2023

Reporting Summary

Nature Portfolio wishes to improve the reproducibility of the work that we publish. This form provides structure for consistency and transparency in reporting. For further information on Nature Portfolio policies, see our Editorial Policies and the Editorial Policy Checklist.

Statistics

For all statistical analyses, confirm that the following items are present in the figure legend, table legend, main text, or Methods section.

n/a	Confirmed	
☐	☒	The exact sample size (*n*) for each experimental group/condition, given as a discrete number and unit of measurement
☐	☒	A statement on whether measurements were taken from distinct samples or whether the same sample was measured repeatedly
☐	☒	The statistical test(s) used AND whether they are one- or two-sided<br>*Only common tests should be described solely by name; describe more complex techniques in the Methods section.*
☐	☒	A description of all covariates tested
☐	☒	A description of any assumptions or corrections, such as tests of normality and adjustment for multiple comparisons
☐	☒	A full description of the statistical parameters including central tendency (e.g. means) or other basic estimates (e.g. regression coefficient) AND variation (e.g. standard deviation) or associated estimates of uncertainty (e.g. confidence intervals)
☐	☒	For null hypothesis testing, the test statistic (e.g. *F*, *t*, *r*) with confidence intervals, effect sizes, degrees of freedom and *P* value noted<br>*Give P values as exact values whenever suitable.*
☒	☐	For Bayesian analysis, information on the choice of priors and Markov chain Monte Carlo settings
☒	☐	For hierarchical and complex designs, identification of the appropriate level for tests and full reporting of outcomes
☐	☒	Estimates of effect sizes (e.g. Cohen's *d*, Pearson's *r*), indicating how they were calculated

Our web collection on statistics for biologists contains articles on many of the points above.

Software and code

Policy information about availability of computer code

Data collection	Qualtrics
Data analysis	All the code is available on OSF (https://osf.io/8yu95/). All analyses were performed in RStudio with the following packages: Hmisc, tidyverse, rstatix, apaTables, ggrepel, knitr, ggpubr, and readxl.<br>RStudio Version 2023.09.0+463. R version 4.3.1.

For manuscripts utilizing custom algorithms or software that are central to the research but not yet described in published literature, software must be made available to editors and reviewers. We strongly encourage code deposition in a community repository (e.g. GitHub). See the Nature Portfolio guidelines for submitting code & software for further information.

Data

Policy information about availability of data

All manuscripts must include a data availability statement. This statement should provide the following information, where applicable:

- Accession codes, unique identifiers, or web links for publicly available datasets
- A description of any restrictions on data availability
- For clinical datasets or third party data, please ensure that the statement adheres to our policy

All de-identified raw data is available on OSF (https://osf.io/8yu95/).

Human research participants

Policy information about studies involving human research participants and Sex and Gender in Research.

Reporting on sex and gender	Study 1 India: 768 participants (247 female, MeanAgeCategory = 2.41, SDAgeCategory= 1.02).<br>Study 1 US: 5,526 participants (3,196 female, MeanAgeCategory = 2.62, SDAgeCategory = 1.29).<br>Study 2A China: 1,086 participants (626 female, MeanAge = 23.31, SDAge = 5.78).<br>Study 2A UK: 1,067 participants (544 female, 12 non-binary, MeanAge = 40.04, SDAge = 13.60).<br>Study 2B, Mexico: 1,053 participants (536 female, 26 non-binary, MeanAge = 24.51, SDAge = 5.43).<br>Study 2B US sample of the same nominal pay: 1,098 participants (652 female, 24 non-binary, MeanAge = 37.28, SDAge = 13.77).<br>Study 2B US sample of the same subjective pay: 1,122 participants (674 female, 25 non-binary, MeanAge = 36.13, SDAge = 13.31).<br>Study 2C South Africa: 649 participants (316 female, 6 non-binary, MeanAge = 28.29, SDAge = 7.45).<br>Study 2C US, sample with the same nominal pay: 662 participants (318 female, 20 non-binary, MeanAge = 36.91, SDAge = 12.62).<br>Study 2C US, sample with the same subjective pay: 662 participants (323 female, 17 non-binary, MeanAge = 36.27, SDAge = 12.52).<br>Study 3A India: 352 participants (83 female, MeanAge = 35.79, SDAge = 8.73).<br>Study 3A US: 382 participants (197 female, 14 non-binary, MeanAge = 37.76, SDAge = 13.99).<br>Supplemental Study 3B US: 537 participants (263 female, 7 non-binary, MeanAge = 41.04, SDAge = 12.39).<br>Study 4 India: 2,065 participants (286 female, 2 non-binary, MeanAge = 24.87, SDAge = 4.90).
Population characteristics	See the “Behavioural & social sciences study design” section.
Recruitment	We recruited data on MTurk and Prolific, filtering the participants by the country in which lived (e.g., India and the US respectively, for our samples in India and the US). In the sample in China, in Study 2b, we recruited participants on social media through links on student networks (QQChat) sent through a collaborator at Hubei University, China. In Study 4, we recruited participants through a partnership Chicago Booth Center for Decision Research has with Ashoka University in Haryana, India. The link was posted through advertisements on the Facebook group “QMaths.” This group has over 280,000 members interested in preparing for competitive exams for jobs in sectors ranging from banking to railways. We selected this Facebook group because 1) Ashoka had an ongoing relationship with one of the moderators of the group and 2) the members would generally be proficient in English and Hindi. In both of these samples, we also filtered participants based on the country in which they lived.
Ethics oversight	All studies were carried out in accordance with all the ethical regulations and were approved by the Institutional Review Board (IRB15-1623 and IRB20-1056) at the University of Chicago. Informed consent was obtained from study participants consistent with the IRB protocol.

Note that full information on the approval of the study protocol must also be provided in the manuscript.

Field-specific reporting

Please select the one below that is the best fit for your research. If you are not sure, read the appropriate sections before making your selection.

☐ Life sciences ☒ Behavioural & social sciences ☐ Ecological, evolutionary & environmental sciences

For a reference copy of the document with all sections, see nature.com/documents/nr-reporting-summary-flat.pdf

Behavioural & social sciences study design

All studies must disclose on these points even when the disclosure is negative.

Study description	Quantitative studies that involve participants completing a behavioral task, effort on which serves as the primary dependent variable.
Research sample	See the “Human research participants” section. Our samples are not representative of all workers in each of the studied countries.
Sampling strategy	For Study 1, we analyzed all the data from participants from a previous study in the conditions that we categorized as either monetary or non-monetary (see Supplementary Table 9a and 9b).<br>For Studies 2a and 2b, our goal was to collect approximately 350 people per condition post-exclusions, which we believed was the maximum number participants we could realistically recruit in Mexico and China. In Study 2c, we pre-registered a total of 665 participants per to have 95% power to detect an effect size of f = 0.14 for the interaction between incentive type (not individual incentive conditions) and culture.<br>Study 3a: We pre-registered 352 participants per country after exclusions (i.e., people who did not rate a single image or those who did not complete the full study and receive payment) to have 80% power to observe a small to medium-sized effect (d = 0.30).<br>Study 3b: We pre-registered a total of 540 US participants to have 90% power to detect an effect of f = 0.14.<br>In Study 4, we wanted to recruit 500 participants per cell post-exclusions. In Study 4, we also only included checks to ensure that

	participants were proficient in both languages. One week before taking the survey for Study 4, participants completed another survey for which they had to report being “very good” or “fluent” speakers of both English and Hindi.
Data collection	Participants completed the study using Qualtrics software on their computers. We did not record whether participants were alone or whether there were other people in their immediate surroundings.
Timing	Study 1: 5/15/2015 - 6/5/2015.<br>Study 2A: The final sample in China consisted of participants recruited from 02/2022 to 04/2022 through three distinct platforms (WeChat: 02/2022 -- 03/2022; University Students: 03/2022; QQChat 03/2022 -- 04/2022); UK: 02/2022 (20% of the sample) and 09/2022 (80% of the sample)--we piloted the study in the UK early on but decided to add a full sample from an additional individualistic country to Study 2 later on in the process, after which we collected more response aiming for 350 participants per cell, analogous to other samples in Studies 2a and 2b.<br>Study 2B: US (Same Nominal Pay): 02/2022; US (Same Subjective Pay): 02/2022; Mexico: 02/2022;<br>Study 2C: US (Same Nominal Pay): 2/6/2023 - 2/7/2023; US (Same Subjective Pay): 2/6/2023 - 2/7/2023; South Africa: 2/6/2023 - 2/7/2023;<br>Study 3A: India: 08/2022; US: 08/2022<br>Supplementary Study 3B: US: 3/31/2023<br>Study 4: India: 11/2022 -- 12/2022
Data exclusions	Exclusion details for all studies are available in section “Exclusion Data and Criteria for Studies 2–4” of the Supplementary Materials. We excluded those who reported not living in the country from which we were recruiting for specific sample, reported being younger than 18 years old, or did not provide consent to participate in the study. Filtering was based either on the internal questions administered by MTurk and Prolific and/or through a question in our Qualtrics Questionnaire. We then followed the following criteria: Among the participants who passed the attention checks, the only criteria for inclusion were that participants should (1) receive the payment code, which meant they got to the end of the survey, (2) rate at least one image, and (3) not complete the same survey more than once. Completing the task could be achieved in one of the two ways: (1) completing the whole 10 minutes of the task and timing out or (2) choosing to quit the task at one of the screens that appeared after every 10 image-ratings and asked whether participants wanted to continue to rate images. For criterion (3), we used Worker IDs or Prolific ID to identify duplicate submissions for the same survey (in Studies 2a and 4 in China and India, this was done through phone numbers and/or email addresses that our collaborators in China and India recorded to compensate participants).<br>In Study 4, we implemented additional checks and exclusions, because we were collecting data from participants on social media. We excluded 825 submissions as they were identified as “Spam” by the (internal) Qualtrics quality-control mechanism. We also removed 2,386 submissions, which were identified by either duplicate phone numbers or email addresses as second or further attempts by the same participant. This number is higher than in the other studies, because participants were recruited on Facebook and nothing prevented participants from using the survey link again, if they wanted to do so. We then implemented the checks from the previous paragraph.
Non-participation	See “Data Exclusions section.”
Randomization	All participants were randomly assigned to conditions through Qualtrics.

Reporting for specific materials, systems and methods

We require information from authors about some types of materials, experimental systems and methods used in many studies. Here, indicate whether each material, system or method listed is relevant to your study. If you are not sure if a list item applies to your research, read the appropriate section before selecting a response.

Materials & experimental systems

n/a	Involved in the study
☒	☐ Antibodies
☒	☐ Eukaryotic cell lines
☒	☐ Palaeontology and archaeology
☒	☐ Animals and other organisms
☒	☐ Clinical data
☒	☐ Dual use research of concern

Methods

n/a	Involved in the study
☒	☐ ChIP-seq
☒	☐ Flow cytometry
☒	☐ MRI-based neuroimaging
